# Polymorph Identification for Flexible Molecules: Linear Regression Analysis of Experimental and Calculated Solution- and Solid-State NMR Data

**DOI:** 10.1021/acs.jpca.3c07732

**Published:** 2024-03-01

**Authors:** Mohammed Rahman, Hugh R. W. Dannatt, Charles D. Blundell, Leslie P. Hughes, Helen Blade, Jake Carson, Ben P. Tatman, Steven T. Johnston, Steven P. Brown

**Affiliations:** † Department of Physics, University of Warwick, Coventry CV4 7AL, U.K.; ‡ Department of Chemistry, University of Warwick, Coventry CV4 7AL, U.K.; § C4X Discovery, Manchester M1 3LD, U.K.; ∥ Oral Product Development, Pharmaceutical Technology & Development, Operations, AstraZeneca, Macclesfield SK10 2NA, U.K.; ⊥ Mathematics Institute at Warwick, University of Warwick, Coventry CV4 7AL, U.K.

## Abstract

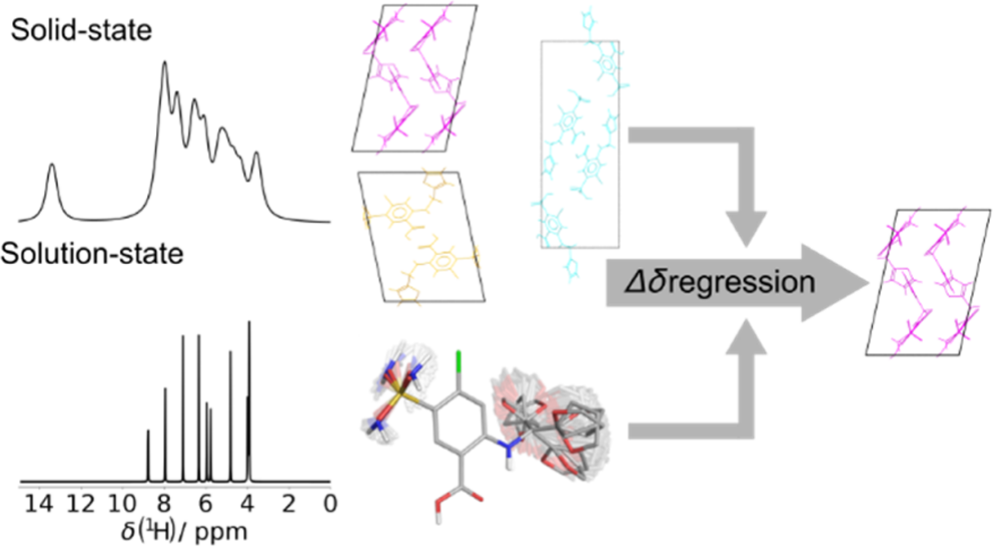

The Δδ regression approach of Blade et al. [J. Phys. Chem. A2020, 124( (43), ), 8959–897732946236 10.1021/acs.jpca.0c07000] for accurately discriminating between solid forms using a combination of experimental solution- and solid-state NMR data with density functional theory (DFT) calculation is here extended to molecules with multiple conformational degrees of freedom, using furosemide polymorphs as an exemplar. As before, the differences in measured ^1^H and ^13^C chemical shifts between solution-state NMR and solid-state magic-angle spinning (MAS) NMR (Δδ_experimental_) are compared to those determined by gauge-including projector augmented wave (GIPAW) calculations (Δδ_calculated_) by regression analysis and a *t*-test, allowing the correct furosemide polymorph to be precisely identified. Monte Carlo random sampling is used to calculate solution-state NMR chemical shifts, reducing computation times by avoiding the need to systematically sample the multidimensional conformational landscape that furosemide occupies in solution. The solvent conditions should be chosen to match the molecule’s charge state between the solution and solid states. The Δδ regression approach indicates whether or not correlations between Δδ_experimental_ and Δδ_calculated_ are statistically significant; the approach is differently sensitive to the popular root mean squared error (RMSE) method, being shown to exhibit a much greater dynamic range. An alternative method for estimating solution-state NMR chemical shifts by approximating the measured solution-state dynamic 3D behavior with an ensemble of 54 furosemide crystal structures (polymorphs and cocrystals) from the Cambridge Structural Database (CSD) was also successful in this case, suggesting new avenues for this method that may overcome its current dependency on the prior determination of solution dynamic 3D structures.

## 1 Introduction

Achieving and controlling the desired physicochemical properties of an active pharmaceutical agent (API) is often a significant obstacle in the development process for the pharmaceutical industry.^1^ While a stable solid crystalline form provides one of the best means to achieve these goals, success is frequently hampered by the occurrence of other polymorphs.^2^ The intricate energy landscape of both crystallization and crystals means that polymorphs often have closely positioned local energy-minima, and novel polymorphs can consequently continue to emerge throughout the development process.^3−6^ Distinguishing, understanding, and characterizing these forms allows the emergence of unwanted forms to be somewhat derisked, and so various techniques are typically applied to do so, especially X-ray diffraction (XRD) and solid-state nuclear magnetic resonance (NMR) in conjunction with density functional theory (DFT) calculations.^7−9^ Differences in crystal packing interaction networks provide a means to distinguish similar crystal structures, notably by probing local environments derived from NMR chemical shifts.^10−13^ Solid-state NMR provides valuable insight into intermolecular interactions,^14−19^ which can be incorporated into crystal structure prediction (CSP)^20,21^ and machine learning (ML) frameworks,^22−25^ providing, for example, geometry constraints for improved prediction of crystal structures.^26,27^

The integration of solid-state NMR data and *ab initio* calculations of NMR parameters in NMR crystallography presents a valuable approach for determining crystal structures.^8−11,28−44^ Requiring a good starting structural model that is geometry optimized before the calculation of NMR parameters, NMR crystallography is now widely employed in academia and increasingly in industry, particularly to refine and improve the quality of structures derived from both single-crystal and powder X-ray diffraction data.^9−11,26−28,36,37,45^ NMR crystallography can also be used to determine crystal structures *de novo* without X-ray diffraction data by finding the model from a CSP^20,21,46−57^ campaign whose calculated properties are most consistent with the experimental NMR data.^7,15−18,26,37,52,58−63^ Chemical shifts for proposed model structures are calculated, usually using the gauge-including projector augmented wave (GIPAW) method,^64−66^ and compared directly with experimentally measured solid-state NMR chemical shifts, with only the correct model expected to pass the given thresholds of agreement for the root mean squared error (RMSE).^11,34,37,67^

Providing structural models for NMR crystallography by CSP is, however, computationally expensive because of the number of possible ways molecules can be packed together.^52^ Conformational polymorphism, where a different torsion angle value exists for a flexible part of a molecular component, adds additional complexity, requiring separate calculation runs for each putative conformation.^48,50,51^ When several rotatable bonds are present, an extremely large set of likely conformations is often generated, with an even greater set of structural models as each conformation’s packing is explored.^68,69^ Yet more structural models are generated by CSP when the number of molecules in the asymmetric unit cell is greater than one.^52,68^

Previously, we developed a novel approach for quantitatively assessing proposed crystal structural models using a combination of solid-state magic-angle spinning (MAS) NMR data with solution-state conformational and chemical shift data.^70^ We showed that for tolfenamic acid, correlating measured differences in chemical shifts between solution and solid states (Δδ_experimental_, eq 1; i.e., the observed change in chemical shift due to crystallization) against their calculated chemical shift differences (Δδ_calculated_, eq 2) allowed us to precisely differentiate and accurately identify the correct structural model from a pool of comparable conformational and packing polymorphs.^70^ A strong correlation is only achieved between Δδ_experimental_ and Δδ_calculated_ when the crystal structural model is correct, and since the solution-state conformational behavior and chemical shifts are easily measured, this approach has clear potential for solving crystal structures *de novo* from CSP crystal structural models, complementing the established RMSE approach.^37^
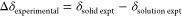
1
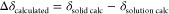
2

The two aspects of conformational selection and molecular packing upon crystallization from solution both contribute to the observed differences in chemical shift between the solution state (which adopts an ensemble of conformations surrounded by diffusely arranged solvent molecules) and the solid state (which adopts one or a few discrete conformations packed against other molecules in precise 3D-arrangements).^71^ Some chemical shifts can be much more sensitive to local conformation than packing interactions, meaning that there is potential to predict conformations that can and cannot satisfy these experimental data points before packing arrangements are attempted.^71^ In the case of tolfenamic acid, we were able to use this principle to correctly calculate narrow ranges of possible conformations present in the solid state that could satisfy the NMR data for two conformational polymorphs in the absence of any packing model.^70^ In this manner, our approach could also be used before a CSP campaign to reduce the conformational searching burden by giving a small, focused set of conformations to propose packing arrangements for, i.e., further facilitating *de novo* determination of crystal structures by NMR crystallography.

**Figure 1:**
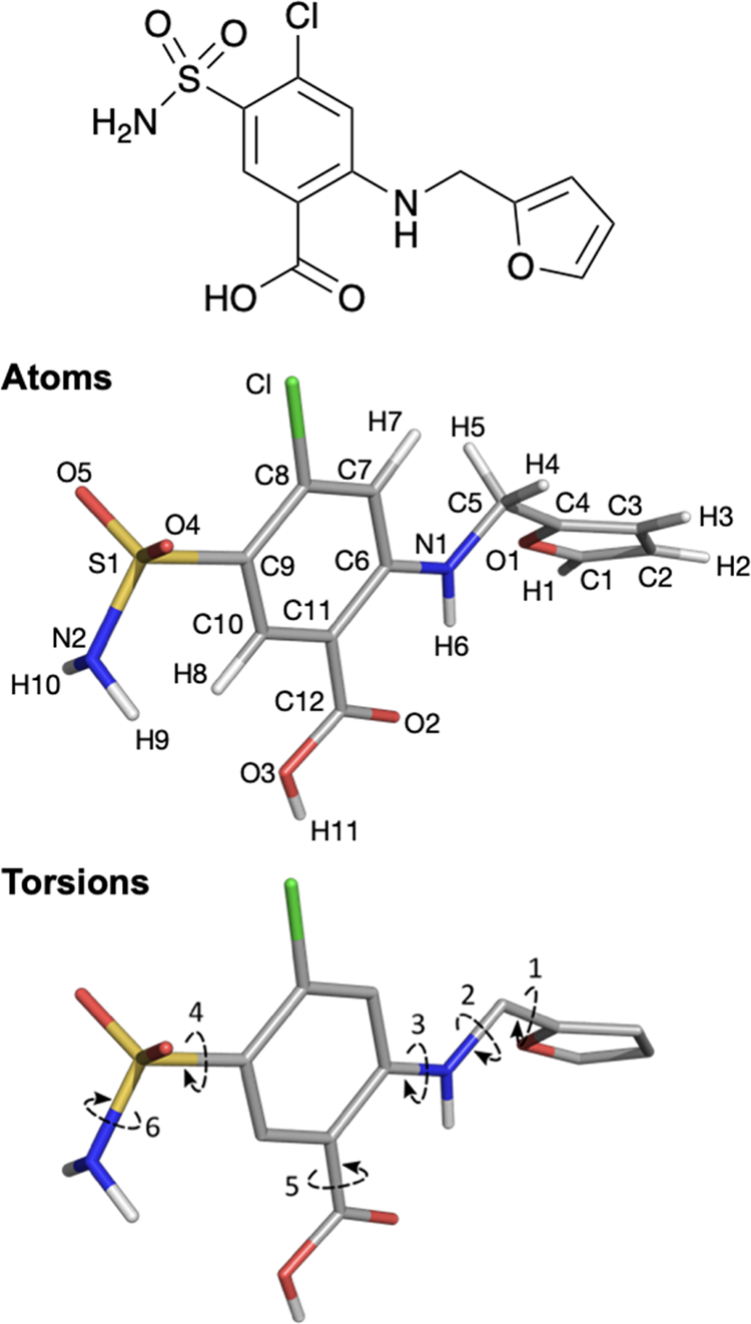
(Top) Two-dimensional (2D) structure of furosemide. (Middle) A conformation of furosemide showing atom nomenclature and torsional degrees of freedom. (Bottom) Torsion definitions are (1) O1–C4–C5–N1, (2) C4–C5–N1–C6, (3) C5–N1–C6–C11, (4) C8–C9–S1–N2, (5) C6–C11–C12–O3, and (6) C9–S1–N2–H9. Atoms are colored by element (carbon gray), and nonpolar hydrogens have been omitted for clarity.

The applicability of the previous work was reduced, however, by being demonstrated on tolfenamic acid,^70^ a compound with only one conformational degree of freedom. Addressing this, we here adapt and apply the approach to furosemide, a molecule with six rotatable bonds (Figure 1), to better exemplify molecules with conformational diversity more typical of those tackled in CSP campaigns.

Crystal structures for furosemide have been determined by X-ray diffraction in three different polymorphs at a range of temperatures (Cambridge Structural Database, CSD, entries are summarized in Table 1).^72−77^ Form I has predominantly been reported with *Z* = 4 and *Z*′ = 2, i.e., with two different conformations in its asymmetric unit. For ease of discussion, the two distinct conformations present in Form I will be referred to as Molecules A and B; these are easily distinguished by their torsion 1 values of +68 and −58°, respectively (see Figure S1). Both structures of Form II have *Z* = 4 and *Z*′ = 1, with FURSEM15 exhibiting conformational disorder at room temperature as the furan ring adopts an additional conformation of approximately 25% occupancy that is not seen at 100 K; there are no coordinates provided for the hydrogen atoms on the disordered furan ring. Form III has *Z* = 2 and *Z*′ = 1. All forms have furosemide in its uncharged state, with dimer pairs formed by reciprocal hydrogen bonding between carboxylic acid groups of adjacent molecules. Nevertheless, the dimer packing is very different between the forms, with the crystal packing similarity tool^27^ within Mercury reporting that they have only 1 or 2 of 15 molecules in common locations between forms (see Table S1). The conformations between the forms also differ, with the differences being principally located at torsions 1, 2, and 4 (see Figures S1 and S2).

**Table 1:** Crystal Structure Information for Furosemide Polymorphs in the CSD

Form	CSD entry ID	space group	*Z*^e^	*Z*′^f^	temperature (K)	date	reference
Form I	FURSEM13	*P*1	4	2	100	2010	(75)
	FURSEM18	*P*1	4	2	120	2016	(72)
	FURSEM03	*P*1	4	2	173	2007	(73)
	FURSEM01	*P*1	4	2	295	1978	(76)
	FURSEM17^a^	*P*1	4	2	293	2012	(77)
	FURSEM02^b^	*P*1	2	1	295	1983	(78)
	FURSEM^c^	*P*1	2	1	295	1976	(74)
Form II	FURSEM14	*P*21/*n*	4	1	100	2010	(75)
	FURSEM15^d^	*P*21/*c*	4	1	293	2010	(75)
Form III	FURSEM16	*P*1	2	1	100	2010	(75)

a This structure has a carboxylic acid hydrogen atom in an incorrect position on one of the molecules (see the text and Figure S3).

b There are no hydrogen atoms available for this structure, so they were added using Mercury; see Section 2.7.

c There are no 3D coordinates available for this structure, so it was not included further in this study.

d This structure is modeled with disorder over two distinct conformations of the furan ring.

e Refers to the number of molecules in the unit cell.

f Refers to the number of symmetry-independent molecules in the crystal structure, i.e., the number of molecules in the asymmetric unit.

Three Form I CSD entries (FURSEM17, FURSEM02, FURSEM)^74,76,77^ have several difficulties compared to the others (denoted by the dashed line in Table 1 and subsequent tables). FURSEM has no 3D coordinates available and so could not be used further in this study.^74^ FURSEM02 has been solved in the same space group as all of the other Form I structures but has been modeled with *Z* = 2 and *Z*′ = 1, in marked contrast to all of the other Form I structures, which have *Z* = 4 and *Z*′ = 2; it also has no hydrogen atoms in its coordinates.^76^ FURSEM17 is nearly identical to FURSEM01, but visual inspection reveals that the carboxylic acid hydrogen in one of the molecules does not form the expected reciprocal carboxylic hydrogen-bonded dimer arrangement (see Figure S3).^77^ A computational study has demonstrated that the lattice energy for the optimized FURSEM17 structure is ∼25 kJ/mol greater than that of FURSEM01,^72^ corroborating the visual result that this hydrogen has been incorrectly placed during structure determination. The incorrect placement of this hydrogen has consequent effects on the sulfonamide nitrogen sp^2^/sp^3^ geometry, which forms hydrogen bonds to the carboxylic acid group.

Using the published crystal structures, we here show that, with an adaption of our approach to reflect the more complex conformational behavior of furosemide in solution, we are able to discriminate between the polymorphs of furosemide using only NMR data and computation. The inconsistencies in the published crystal structures are also readily identifiable, even those as apparently subtle as that of FURSEM17. The approach is differently sensitive to the popular RMSE method, exhibiting a much greater dynamic range. Moreover, the approach is observed to operate equally well with fixed or variable unit cell parameters during geometry optimization. Some care should be taken in the choice of solvent used to measure solution-state data, especially that the molecule’s charge state is the same between solution and solid states. We also show that, in the absence of an experimentally determined solution conformational ensemble (here termed a “solution dynamic 3D structure”), the solution conformational behavior can, at least in this case, be adequately approximated from a sufficiently large collection of furosemide crystal structures (also comprising neutral solvates and cocrystals from the CSD), suggesting potential avenues for widening the approach’s availability to researchers not able to readily access solution dynamic 3D structures.

## 2 Methods

A detailed workflow for the approach is given in Figure S4. Specific details relating to this study are as follows.

### 2.1 Software

Statistical analysis was performed using the Python 3.8 programming language with the pandas V1.0.5, NumPy V1.18.5, and SciPy V1.5.0 libraries; the source code is available on GitHub (https://github.com/MKRahman97/NMR-Scoring-Function) and includes a graphical user interface that aids performing the calculations (see the user guide at the end of the SI). Matplotlib V3.2.2 was used to produce graphs. Mercury^79^ (CSD database V5.42, November 2020 data library) was used for the 3D visualization and comparison of crystal structures. Structural figures were created using PyMol V2.3.5.

### 2.2 Materials and Sample Preparation for Solution-State NMR

Furosemide, reference compounds, and all solvents were purchased from Sigma-Aldrich (Gillingham, U.K.). All samples contained dilute DSS-*d*_6_ (0.3 mM). All solution NMR spectra were acquired at 25 °C unless specified otherwise.

From its uncharged solid form as supplied, furosemide was found to be insoluble in CDCl_3_. A stock solution of 100 mM furosemide in pure DMSO-*d*_6_ was therefore made. From this, three 600 μL solution NMR samples were made for chemical shift measurements: 10 mM furosemide in 100% DMSO-*d*_6_ and two largely aqueous samples of 2.5 mM furosemide in an 80:20 (v/v) mixture of D_2_O/DMSO-*d*_6_. Due to the low solubility of furosemide in water, samples with higher furosemide concentration or higher proportion of water could not be made without precipitation, and a compromise had to be made to have a high enough compound concentration in a largely aqueous environment to still permit accurate measurement of ^13^C chemical shift data at ^13^C natural abundance. By following the titration of the chemical shifts with decreasing pH for a 1 mM 80:20 H_2_O/DMSO-*d*_6_ sample, the p*K*_a_ value of the carboxylic acid moiety in this solvent mixture was determined as 4.11 ± 0.05 (see Figure S5). The two 80:20 D_2_O/DMSO-*d*_6_ samples were then pH-adjusted using dilute DCl and NaOD to yield one of the uncharged species, i.e., the carboxylic acid, at pH 2.11 and one of the carboxylate anion at pH 6.77.

The concentration dependence of the ^1^H chemical shifts was investigated by creating further samples in pure DMSO-*d*_6_ from the stock solution with concentrations ranging from 100 μM to 50 mM. The absence of a concentration-dependent variation of chemical shifts indicates no substantial dimerization of furosemide in DMSO-*d*_6_ solutions at the concentrations studied (see Figure S6).

### 2.3 Measurement of Solution-State NMR Chemical Shifts (δ_solution expt_) and Dynamic 3D Structure

Solution-state NMR experiments were performed on a Bruker Avance III spectrometer operating at a ^1^H Larmor frequency of 500.13 MHz using a 5 mm QXI ^1^H/^13^C/^15^N/^19^F/^2^H probe.

Chemical shifts were measured for the uncharged form of furosemide in pure DMSO-*d*_6_ and for both charged (pH 6.77) and uncharged (pH 2.11) forms in a largely aqueous environment (80:20 v/v D_2_O/DMSO-*d*_6_). ^1^H–^13^C-HSQC, ^1^H–^13^C-HMBC, and ^1^H–^15^N-HSQC NMR spectra were used to assign all ^1^H, ^13^C, and ^15^N nuclei in the three conditions (portions of spectra and acquisition parameters are given in Figure S7 and Table S2, respectively). ^1^H chemical shifts were referenced relative to internal *d*_6_-DSS. ^13^C chemical shifts were referenced indirectly using the Ξ factors for *d*_6_-DSS and TMS (25.145020, 25.144953, respectively).^80^

To re-reference ^1^H chemical shifts measured from *d*_6_-DSS in DMSO-*d*_6_ to absolute (i.e., TMS in CDCl_3_), a first correction of −0.0285 ppm was applied to adjust the values for referencing relative to TMS in DMSO-*d*_6_, which was measured directly from a DMSO-*d*_6_ sample containing both TMS and *d*_6_-DSS. A second correction of +0.074 ppm was then applied to adjust for the difference between TMS in DMSO-*d*_6_ compared to CDCl_3_ (as per the procedure described by Hoffman et al.^81^): ^13^C chemical shifts measured relative to *d*_6_-DSS in DMSO-*d*_6_ had the same adjustments, with an additional −2.6194 ppm, to account for the difference in Ξ ratio between DSS and TMS.^81^

To reference ^1^H chemical shifts measured from *d*_6_-DSS in 80:20 D_2_O/DMSO-*d*_6_ to absolute, a first correction of +0.0138 ppm was applied to adjust the values for referencing relative to TMS in 80:20 D_2_O/DMSO-*d*_6_, which was measured directly from a 80:20 D_2_O/DMSO-*d*_6_ sample containing both TMS and *d*_6_-DSS. A second correction of −0.0596 ppm was then applied to adjust for the difference between TMS in 80:20 D_2_O/DMSO-*d*_6_ compared to CDCl_3_, which itself was estimated by taking the proportionate combination of the values for 100% DMSO-*d*_6_ (0.074 ppm) and 100% D_2_O (−0.093 ppm) reported in Hoffman et al.^81^^13^C chemical shifts measured relative to *d*_6_-DSS in 80:20 D_2_O/DMSO-*d*_6_ had the same adjustments, with an additional −2.6194 ppm to account for the difference in Ξ ratio between DSS and TMS.^81^

The dynamic 3D structure of furosemide in its uncharged state in pure DMSO-*d*_6_ was determined according to the method of Blundell et al.^82^ using a combination of ^3^*J*_HH_ values and distance restraints from EASY-ROESY spectra.^83^ Briefly, a population distribution function is defined for each rotatable bond, whereby each torsion has one or more modes (macrostates) that are each characterized as having a population (π), a mean position (μ, the conformer conformation), and an extent of Gaussian libration about the mean position (σ, for the generation of microstates, i.e., discrete conformations). The values of these parameters are iteratively and exhaustively varied until the best fit to all of the structural restraints is obtained.

The representation of the dynamic 3D structure shown below in Section 3.1 was generated by overlaying all of the conformers (i.e., the conformations in which all of the mean positions of all of the modes are permuted together, bright conformations) onto an ensemble of conformations representing the Gaussian libration about those mean positions (faded conformations), which themselves were generated by randomly sampling the population distribution for each and every torsion simultaneously and applying those values to a prior DFT-optimized starting conformer (see Section 2.5).

### 2.4 Source of Solid-State NMR Chemical Shifts (δ_solid expt_)

Experimental ^13^C and ^1^H chemical shifts for furosemide Form I were taken from Widdifield et al.,^72^ who have provided full assignment for both ^13^C and ^1^H nuclei based on recording ^1^H–^13^C CP-HETCOR MAS NMR spectra. To the best of our knowledge, there are currently no published chemical shift values for furosemide Forms II and III.

### 2.5 DFT Methodology

DFT calculations were performed using CASTEP^84^ academic release version 17.21. All calculations (both geometry optimization and NMR chemical shielding calculations) used the Perdew–Burke–Ernzerhof (PBE)^85^ exchange–correlation functional, a plane-wave basis set with ultrasoft pseudopotentials^86^ and a plane-wave cutoff energy of 700 eV.^87,88^ Integrals over the Brillouin zone were taken using a Monkhorst–Pack grid^89^ with a minimum sample spacing of 0.1 × 2π/Å. Unit cell dimensions and angles were fixed during the geometry optimization unless stated otherwise; when unit cell parameters were allowed to vary during geometry optimization, DFT-D dispersion correction was implemented according to the approach of Tkatchenko and Scheffler.^90^ (In these cases, CASTEP version 20.11 was used to overcome a known software bug with retaining symmetry, but the pseudopotentials from version 17.21 were used to ensure consistency of results.) NMR chemical shielding calculations were carried out on the geometry-optimized structures using the GIPAW^64,65^ method to determine the shielding tensor for each nucleus in the crystal structure. Calculations were performed using the University of Warwick Scientific Computing Research Technology Platform (SCRTP) High-Performance Computing clusters. For each structure calculation, two cores of a 14-core Intel Xeon E5-2680 CPU were used, with the geometry optimization taking 3 h on the starting conformer generated structure and magnetic resonance calculations taking an average of 15 min per structure. This indicates that to perform calculations with 1000 conformers to enable the analysis of the dynamic 3D ensemble would take about 506 core hours total.

We emphasize that our approach relies on chemical shielding differences (Δδ) and thereby avoids the issue of referencing the calculated values.^62,91−93^ However, to allow for comparison to the RMSE method,^37^ calculated NMR chemical shieldings, *σ*_calc_, were converted into isotropic chemical shifts (δ_iso,calc_) using eq 3, where σ_ref_ is the reference shielding (30.0 ppm for ^1^H and 169.9 ppm for ^13^C),^33,70^ and the gradient (*m*) was set to minus one; this is equivalent to taking the sum of the experimental chemical shifts and the GIPAW calculated absolute isotropic chemical shieldings.^91^
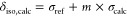
3

### 2.6 Calculation of Solution-State NMR Chemical Shifts (δ_solution calc_) from the Solution Dynamic 3D Structure

The method for determining the values of the parameters for the torsional behavior in a dynamic 3D structure assumes an underlying base 3D geometry of fixed bond lengths, bond angles, improper dihedrals, and reasonable torsion values upon which the dynamic 3D information is then layered during the dynamic 3D structure solving process. For an uncharged molecule of furosemide, this “base conformation” was generated using the Mercury conformer generator,^94^ which uses generalized but contextualized searching of the CSD database to assign bond lengths, bond angles, and improper dihedral angle values directly from appropriate experimental data. As described previously,^70^ to perform self-consistent DFT calculations of NMR chemical shifts for the solution state using CASTEP, this Mercury-generated base conformation needs to first be geometry-optimized in CASTEP before the dynamic 3D information is layered back on top of it. This optimization was achieved by isolating the molecule from its neighbors by placing the template conformation in a periodic repeating unit cell of 10 Å in all dimensions (an “isolated box”) and then performing CASTEP DFT optimization.^87,95,96^ The Mercury-generated base conformation and its subsequently geometry-optimized shape barely differ, with a heavy-atom RMSD of 0.053 Å and an all-atom RMSD of 0.054 Å (see overlay in Figure S8). The largest change was observed in the sulfonamide moiety, with the nitrogen developing a slightly more sp^3^ character upon optimization.

Previously,^70^ at this point, we had calculated the solution-state NMR chemical shift (δ_solution calc_) by (1) systematically and exhaustively creating conformations by rotating each torsion upon the base geometry at 15° intervals, (2) calculating ^1^H and ^13^C NMR chemical shifts for each conformation using the GIPAW method in CASTEP for an “isolated box”,^87^ and then (3) calculating the overall solution-state value of each ^1^H and ^13^C chemical shift by combining the contribution of each conformation to its observed chemical shift, according to its calculated occupancy as per the measured solution dynamic 3D structure. While this approach was suitable for tolfenamic acid because it only had 1 rotatable bond, it was unfeasible for furosemide because its 6 rotatable bonds would have required isolated box CASTEP calculations on approximately 100 million conformations (24^6^). Instead, we modified this part of the approach for calculating the solution-state chemical shift as per the workflow in Figure S4 by (1) generating, by Monte Carlo random sampling,^97^ a random ensemble of conformations at all torsions simultaneously by sampling from their experimentally determined Gaussian probability distributions, (2) serially performing “isolated box” GIPAW calculations in CASTEP on each conformation, (3) keeping a running average of each ^1^H and ^13^C chemical shift as conformations were included in the ensemble, and (4) repeatedly adding random conformations until each and every chemical shift value had converged to a value with an error comparable to that of experimental measurement (^13^C ± 0.1 ppm and ^1^H ± 0.2 ppm).^98,99^

Convergence of the chemical shift with increasing ensemble size was tested using ensemble sizes in the range of 10–1000. Convergence for the independent and identically distributed (IID) conformations can be treated by the central limit theorem (CLT),  with a mean (μ), standard deviation (σ), and sample size (*n*).^100^ Standard errors and confidence intervals were also estimated by the method of bootstrapping with replacement for a sample of size *N* = 1000, which was repeated *M* = 1000 times.^101^

Calculation of the solution-state NMR chemical shifts (δ_solution calc_) for furosemide in the charged state was performed in the same manner, except that the base conformation had the carboxylic acid hydrogen removed before CASTEP DFT geometry optimization.

The δ_solution calc_ value for the substitute ensemble of furosemide conformations from the CSD for the solution dynamic 3D structure was also calculated in the same manner (see Section 2.9).

### 2.7 Calculation of Solid-State NMR Chemical Shifts (δ_solid calc_)

To calculate solid-state chemical shift values (δ_solid calc_) for the crystal structures in Table 1, each crystal structure from the Cambridge Structural Database was subjected to DFT geometry optimization with fixed unit cell dimensions and then chemical shift calculations using the GIPAW method in CASTEP (as detailed in Section 2.5). DFT-D geometry optimization with variable unit cell dimensions was used only for the results in Section 3.7.

The crystal structure of FURSEM02 has no coordinates for hydrogen atoms, which needed to be added before DFT calculations could be performed. Hydrogens were therefore added using the automatic tool in Mercury, which incorrectly placed the carboxylic acid group hydrogen on O2 (since the C12–O2 bond length in the carboxylic acid group at 1.263 Å is fractionally shorter than the C12–O3 bond length at 1.274 Å, the hydrogen should be placed on O3 as the oxygen with least double bond character; refer to Figure 1 for atom nomenclature). Since this was moreover incompatible with the crystal structure’s obvious hydrogen-bonding network, the carboxylate hydrogen was manually moved to O3 in the correct configuration for hydrogen-bonding (as in the other Form I structures, refer to Figure S3).

### 2.8 Linear Regression of Δδ_calculated_ vs Δδ_experimental_ and *t*-Test to Identify the Correct Form

As per eq 1, the chemical shifts measured in solution (δ_solution expt_) were subtracted from those reported for solid Form I in a study by Widdifield et al.^72^ (δ_solid expt_) to give Δδ_experimental_ values for each nonexchangeable ^1^H and ^13^C nucleus. Likewise, as per eq 2, the calculated values in solution (δ_solution calc_) were subtracted from the calculated values for each crystal structure (δ_solid calc_) to give a corresponding set of Δδ_calculated_ values.

For each set of ^1^H and ^13^C nuclei, the Δδ_calculated_ and Δδ_experimental_ values were then plotted against each other (on the *y* and *x* axes, respectively), with the graph then fit to the simple linear equation *y* = *mx* + *c.* The coefficient of determination (*r*^2^) and Pearson Correlation Coefficient (*r*) were calculated. One-tailed *t*-tests were used to test the statistical significance of any positive correlation, with *p*-values determined by eqs 4 and 5
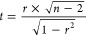
4
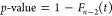
5where *n* is the sample size and *r* is the Pearson correlation coefficient,^102^ used to determine the *p*-value from the *t*-distribution tables with two degrees of freedom (*F*_*n*–2_). This test was run using a null hypothesis of *m* = 0 (no correlation) and the alternative hypothesis of *m* > 0 (positive correlation). *p*-Values less than 0.05 reject the null hypothesis at the 95% confidence interval and indicate a significant positive correlation, i.e., the calculated difference in chemical shift between the solution and solid states significantly agrees with the experimentally observed difference in chemical shift. No mathematical correction was implemented for multiple comparisons, and therefore, in the case that the null hypothesis is true in all cases, false positives (indicating significant findings where none exist) would be expected to occur in 5% of cases.

### 2.9 Use of an Ensemble of Crystal Conformations of Furosemide to Approximate δ_solution calc_

To explore whether a large collection of furosemide conformations from crystal structures could sufficiently mimic the solution dynamic 3D structure and thereby provide a substitute ensemble for calculating δ_solution calc_ in the absence of a measured dynamic 3D structure, the torsion angle values from all CSD structures containing coordinates of furosemide in its neutral state (i.e., pure polymorphs, neutral solvates, and cocrystals) with an *R*-factor <10% and no disorder were extracted from the database (see Table S30). To use these data to approximate a solution ensemble, the torsion values from each individual conformation were applied to the base conformation; these conformations were then collected into an ensemble whose chemical shifts were then calculated as described in Section 2.6. Note that, formally, this substitute ensemble reflects the conformational space that neutral furosemide occupies in the solid state, whereas the dynamic 3D structure ensemble reflects the conformational space that neutral furosemide actually occupies in solution.

## 3 Results and Discussion

The 2D molecular structure of furosemide, its atom numbering, and its torsion definitions are shown in Figure 1.

### 3.1 Measurement of Solution-State NMR Chemical Shifts (δ_solution expt_) and Dynamic 3D Structure

Chemical shifts are solvent and charge-state dependent and therefore an important consideration in measuring them was the choice of an appropriate deuterated solvent that would maintain furosemide in the same charge state as present in the crystal forms (i.e., neutral). While CDCl_3_ would have been the natural starting point (as previously in Blade et al.^70^), furosemide in its uncharged form is insufficiently soluble in CDCl_3_ to allow ready measurement of its solution-state chemical shifts and dynamic 3D structure. Pure DMSO-*d*_6_ was therefore used instead. ^1^H and ^13^C chemical shifts for neutral furosemide measured in dry DMSO-*d*_6_ at 25 °C and 10 mM relative to internal DSS-*d*_6_ are given in Table 2, constituting values for δ_solution expt_. There was no observed dependence of chemical shift with concentration up to 50 mM in DMSO-*d*_6_ (see Figure S6).

**Table 2:** Experimentally Measured and Calculated DFT GIPAW NMR Chemical Shifts for Furosemide in Solution and in Solid-State Forms

	experimentally measured δ (ppm)			calculated δ (ppm)															
	δ_solution expt_^a^	δ_solid expt_^b^		δ_solution calc_^c^	δ_solid calc_^d^														
		Form I, Molecule			Form I, Molecule											Form II			Form III
nucleus		A^e^	B^e^		13^f^ A^e^	18 A	03 A	01 A	13 B^e^	18 B	03 B	01 B	17 A	17 B	02	14	15 (75%)^g^	15 (25%)	16
C1	143.3^h^	141.5^h^	144.2	142.5	142.3	142.4	142.4	142.5	146.5	146.1	145.6	145.7	143.6	144.5	144.5	149.2	146.6	148.7	145.3
C2	111.1	112.1	110.5	108.5	113.0	113.0	112.5	112.3	109.8	110.0	109.9	110.3	112.8	110.4	108.6	113.1	115.0	113.7	113.1
C3	108.2	109.4	110.5	106.2	109.8	109.9	109.7	109.6	112.9	112.5	112.1	112.3	111.8	112.5	112.8	115.3	111.8	113.9	109.6
C4	151.9	155.2	150.6	152.8	155.3	155.4	155.4	155.4	152.5	152.1	152.1	152.3	153.1	151.5	150.8	147.3	147.0	147.5	156.3
C5	39.7	39.6	39.6	36.1	36.6	36.8	36.7	36.6	36.5	36.6	36.3	36.5	37.5	36.1	37.6	38.8	39.0	38.3	33.0
C6	152.9	155.2	154.0	149.8	151.6	151.7	151.7	151.7	150.6	150.8	150.7	150.7	151.3	150.9	151.3	148.3	148.7	148.6	151.0
C7	114.1	116.8	117.1	111.9	115.9	116.3	116.2	116.4	117.6	117.3	116.8	116.6	115.6	116.4	116.0	111.2	111.2	111.6	111.8
C8	136.7	138.0	138.0	143.0	142.5	141.9	141.9	142.0	141.4	141.2	141.3	141.5	144.3	140.5	142.5	146.3	145.9	146.3	142.7
C9	127.3	125.2	127.1	131.5	125.5	125.6	125.8	126.0	128.2	128.1	128.0	128.0	125.4	128.6	127.5	130.1	130.1	130.7	130.0
C10	133.8	136.6	135.4	133.0	136.3	136.3	136.0	136.0	134.6	134.6	134.4	134.4	135.0	131.3	134.6	133.8	134.3	133.8	134.7
C11	108.7	106.1	108.6	103.5	105.3	105.2	105.1	104.9	108.1	108.0	107.6	107.0	105.1	107.4	106.0	104.9	105.8	104.8	106.8
C12	169.2	172.2	172.2	169.0	172.8	173.0	173.3	173.5	173.0	173.3	173.4	173.6	172.3	169.2	174.0	174.9	175.0	175.2	176.0
H1	7.7	6.5	7.7	7.1	6.4	6.4	6.4	6.4	7.9	7.9	7.8	7.8	6.4	7.3	7.2	7.8	7.9	7.9	7.2
H2	6.5	6.4	6.0	6.0	6.6	6.6	6.4	6.4	6.1	6.1	6.0	6.0	6.6	6.1	5.2	6.7	7.5	6.7	6.3
H3	6.4	5.7	6.0	5.8	5.2	5.2	5.2	5.1	6.0	6.0	5.9	5.9	5.3	5.8	5.2	5.7	5.8	5.8	6.2
H4*^i^	4.7	4.7	4.3	3.9	4.1	4.2	4.2	4.2	3.9	3.9	3.9	4.0	4.6	3.8	4.3	3.8	3.6	3.8	3.4
H6	8.7	8.4	8.4	8.8	7.9	7.9	8.0	8.0	8.3	8.3	8.3	8.5	7.6	9.3	8.6	7.9	8.1	7.9	8.3
H7	7.1	7.9	6.2	6.4	7.3	7.2	7.2	7.2	5.0	5.0	5.0	5.0	6.6	5.0	6.8	4.4	4.9	4.5	5.6
H8	8.5	8.7	8.6	8.0	8.1	8.1	8.0	8.1	8.0	8.0	8.0	8.0	8.1	7.7	8.1	7.3	7.3	7.3	8.0
H9*^j^	7.4	6.5	6.7	4.0	6.4	6.5	6.5	6.4	7.0	7.1	7.1	7.1	5.5	5.4	6.8	7.0	6.7	6.7	6.2
H11	13.4	12.7	12.7	4.8	13.3	13.5	13.5	14.2	13.5	13.8	13.7	14.3	8.8	10.2	14.7	12.9	13.3	12.7	13.3

a Chemical shifts of furosemide in DMSO-*d*_6_ at 10 mM and 25 °C, referenced relative to absolute TMS in CDCl_3_ as follows: Direct (^1^H) and indirect (^13^C) referencing relative to internal *d*_6_-DSS, then corrections of ^1^H + 0.0455 and ^13^C – 2.6194 (see Sections 2.2 and 2.3). Raw values are given in Table S22.

b Data taken from Widdifield et al.^72^ (see Section 2.4).

c Solution-state NMR chemical shifts were calculated as described in Sections 2.5 and 2.6.

d Solid-state NMR chemical shifts were calculated as described in Sections 2.5 and 2.7, following geometry optimization of atomic positions with fixed unit cells.

e Form I has two molecules in the asymmetric unit, which can be readily distinguished by their torsion 1 values (A ≅ 68°, B ≅ −58°).

f CSD FURSEM entry ID (refer to Table 1).

g FURSEM15 has disorder around the furan ring, occupying two sites at 75 and 25% occupancy, respectively.

h Measurement errors are in ppm. In solution: ^1^H ± 0.001 and ^13^C ± 0.020;^82^ in the solid-state: ^1^H ± 0.2 and ^13^C ± 0.1.^98,99^

i H4 and H5 have identical chemical shifts in solution due to the absence of a chiral center in the molecule, manifesting as a single resonance, labeled H4*. The solid-state NMR chemical shifts are given as the mean of H4 and H5.

j The sulfonamide hydrogens (H9, H10) are in rapid exchange in solution and manifest in spectra as a single broadened resonance, labeled H9*. The solid-state NMR chemical shifts are given as the mean of H9 and H10.

**Figure 2:**
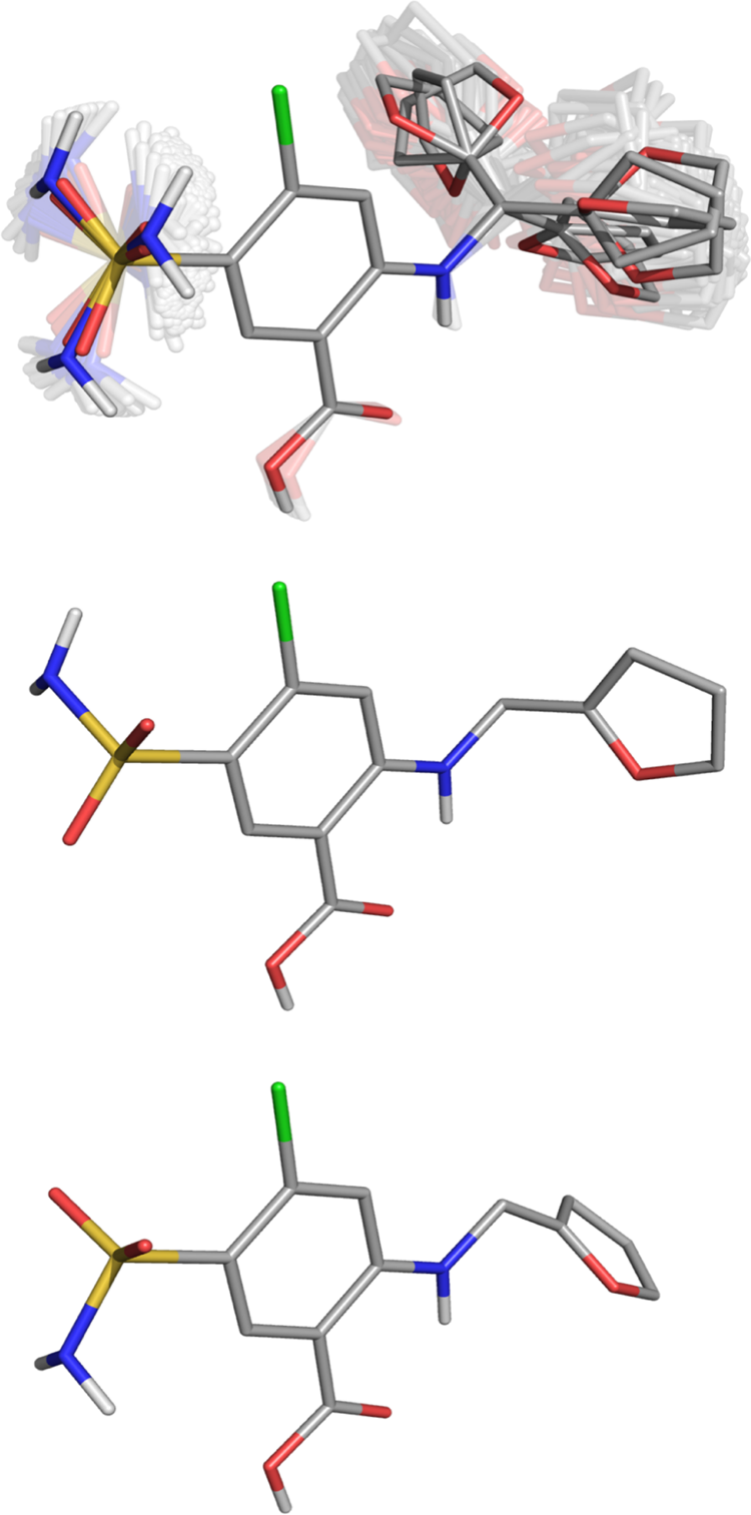
Solution dynamic 3D structure of furosemide in a conformational ensemble representation. (Top) All of the conformers (i.e., the mean positions of all of the modes permuted together; bright conformations) are overlaid together on the central aromatic ring as an ensemble of conformations representing the Gaussian libration about those mean positions (faded conformations). The Gaussian probability distributions determined for each torsion that underlie this representation are shown in Figure 3. (Middle, Bottom) Two different single conformation conformers from the dynamic solution 3D structure; the values for their individual torsions are shown in Figure 3 by dotted (middle) and dashed lines (bottom). Atoms are colored by element (carbon gray), and nonpolar hydrogens have been omitted for clarity.

**Figure 3:**
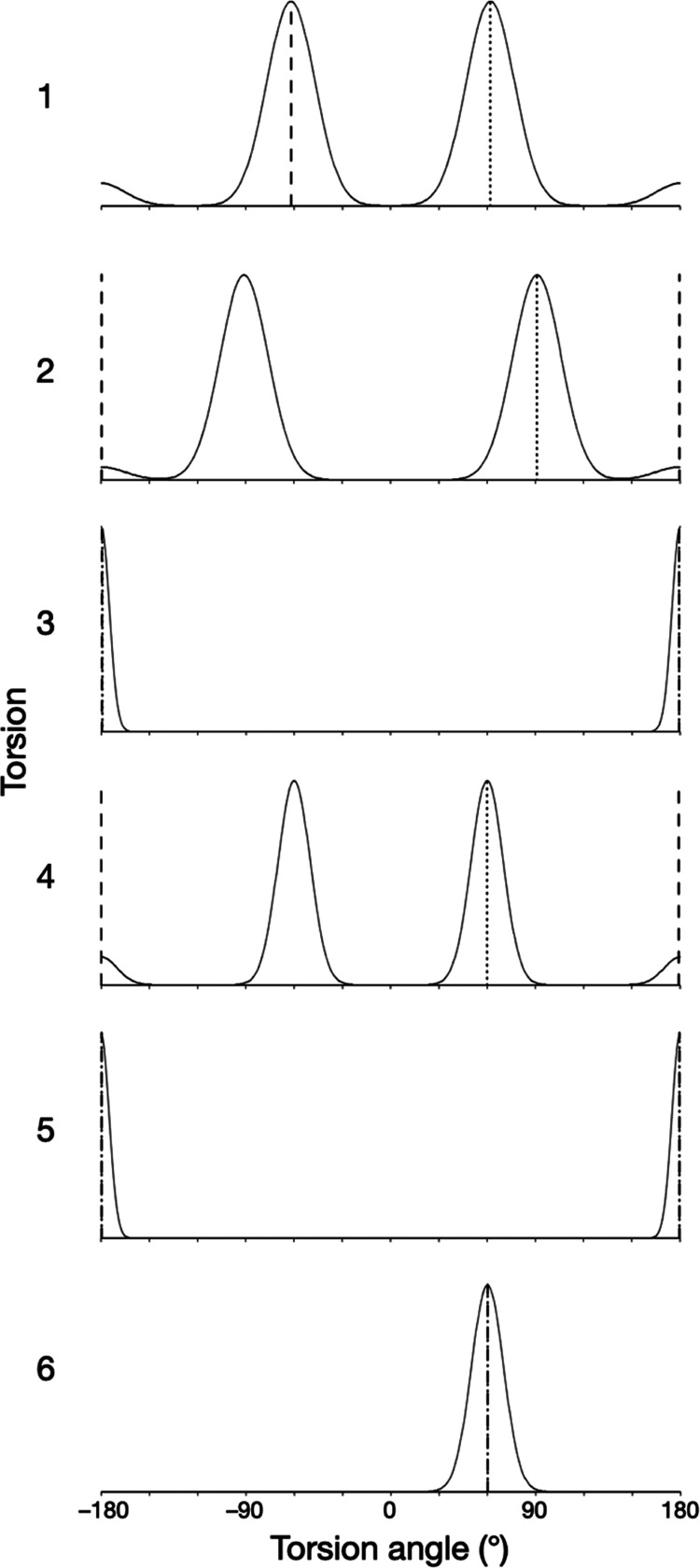
Solution dynamic 3D structure of furosemide in a torsion-population representation. For each torsion, the changing population with torsion angle (*x*-axis) is given relative to its maximum occupancy (*y*-axis). Each torsion has a dynamic behavior that is described as one or more modes that each have a Gaussian libration about their central (mean) values. The torsion values of the conformers shown in Figure 2 are indicated with dotted and dashed lines (middle and bottom, respectively). All conformational parameter values of the solution dynamic 3D structure are given in Table S3.

The dynamic 3D structure of furosemide was solved in its neutral state in pure DMSO-*d*_6_ at 25 °C according to the method of Blundell et al.^82^ Furosemide has 6 rotatable torsions (see Figure 1), which each has its own distinct conformationally dynamic behavior. Conformational population distributions for each torsion were determined as one or more modes of potentially differing occupancies (macrostates), each of which was modeled as Gaussian librations (microstates) about mean torsion values. All conformational parameter values for the determined solution dynamic 3D structure are given in Table S3, and different representations of these data are shown in Figures 2 and 3. In Figure 2, all of the conformers (i.e., the mean positions of all of the modes permuted together; bright conformations) are overlaid together onto an ensemble of conformations representing the Gaussian libration about those mean positions (faded conformations). The Gaussian probability distributions determined for each torsion that underlie this representation are shown in Figure 3.

Torsions 3 and 5 both adopt tightly distributed, unimodal behaviors in solution because of an intramolecular hydrogen bond between atoms H6 and O2. The aniline H6 temperature coefficient (Δδ_H6_/Δ*T* = −2.2 ppb/K; 10 mM, neutral state in pure DMSO-*d*_6_, see Figure S9) is noticeably suppressed compared to the value expected for unrestricted exchange with residual water (which has a measured temperature coefficient itself of −5.0 ppb/K), providing strong evidence that this hydrogen bond persists even in strongly competing solvents. (This hydrogen bond is also present in all furosemide polymorph structures.) The sulfonamide H9* temperature coefficient (Δδ_H9_/Δ*T* = −4.7 ppb/K) is similar to the value of the residual water (−5.0 ppb/K), indicating that it is not involved in intramolecular hydrogen bonding. The hybridization states of the two nitrogen atoms are quite different, with ^1^*J*_HN_ coupling constants of −94.1 Hz for H6 (i.e., mostly sp^2^ character and geometry) and −82.6 Hz for H9* (i.e., mostly sp^3^) in DMSO-*d*_6_ (see Figure S9).

Torsions 1, 2, and 4 give rise to most of the significant variation in shape that furosemide displays in solution. Torsion 4 has trimodal behavior. Torsions 1 and 2 each manifest essentially trimodal behaviors but have some codependence and, therefore, do not permute together to produce nine conformations: rather, their combined behavior produces only eight measurably populated shapes, with the *trans–trans*/180°–180° combination being absent in solution (or at least below the measurable limit). Overall, therefore, furosemide librates around 24 (3 × 8) conformers in solution.

The conformations furosemide adopts in the solution state are compared to those in its three polymorphs in Table S4 and Figures S10 and S11. Although all three forms differ from each other in crystal conformation, nevertheless, all solid-state conformations lie within the distribution of values observed in solution, i.e., although each form adopts a different conformation in the solid state, they all nevertheless exhibit conformations that are reasonably populated in solution.

### 3.2 Calculation of Solution-State NMR Chemical Shifts (δ_solution calc_) from the Solution Dynamic 3D Structure

Solution-state NMR chemical shifts were calculated from the solution dynamic 3D structure as follows. The presence of 6 rotatable bonds in furosemide meant that the method we used previously for tolfenamic acid^70^ (of systematically and exhaustively creating conformations and using their solution occupancy to calculate their contribution to the observed chemical shift) was impractical because it would have required DFT calculations on the order of 100 million conformations (15° increments gives 24^6^ ≅ 190 million). Instead, we modified the workflow to be (1) generating, by Monte Carlo random sampling, a random ensemble of conformations at all torsions simultaneously by sampling from their experimentally determined Gaussian distributions, (2) serially performing GIPAW “isolated box” calculations on each conformation, (3) keeping a running average of each chemical shift as conformations were included in the ensemble, and (4) repeatedly adding random conformations until each and every chemical shift value had converged to a value with an error comparable to that of experimental measurement (^13^C ± 0.1 ppm, ^1^H ± 0.2 ppm)^98,99^ (see Sections 2.5 and 2.6, and Figure S4 for workflow diagram). Values for δ_solution calc_ are given in Table 2.

In this case, an ensemble size of *N* = 1000 conformations gives standard deviations for the calculated chemical shifts that are less than that of experimental measurement for all nuclei except C3 (±0.11) and C7 (±0.16) (see underlined values in Table S5). Ensemble sizes of *N* = 500–1000 conformations are adequate for all calculated chemical shifts to converge within the error of these values (see bold values in Table S5), and indeed most ^13^C values and all ^1^H values had converged within the error of these final values after averaging from 100 randomly sampled conformations. Histograms of the calculated chemical shifts for the ensemble of *N* = 1000 conformations are shown for each and every nucleus in Figure S12. The distributions are complex, reflecting the complex dependence of each chemical shift upon molecular conformation as all of the torsions vary together. That is, different modes of each torsion, and combinations of modes across multiple torsions, can give rise to conformations with distinctive chemical shift values, which are visible as separate clusters of values in these histograms. Not surprisingly, when compared against a normal distribution with a quantile–quantile plot (Q–Q plot), nuclei with more complex, non-normal distributions of calculated chemical shift deviate substantially from the identity line (e.g., H9, C10) (see Figure S13A). This is in contrast to the simple case of tolfenamic acid described previously,^70^ which, having only one rotatable bond, gave a transparently interpretable pattern of chemical shift variation with the torsion angle value for each nucleus. In that case, it was straightforward to predict a well-defined range of possible torsion angle values (and attendant conformations) in each solid form from the experimental solid-state chemical shift values. In this case of furosemide, despite being more complex, these distinct regions within the chemical shift histograms nevertheless relate to particular conformations, leading to the possibility that, at least in some cases, a given set of solid-state chemical shifts could likewise be identified with defined conformers and conformations from the solution ensemble. It therefore seems probable that in this manner, conformations can be identified for input into CSP campaigns that are upfront more likely than others to satisfy the solid-state NMR chemical shifts, i.e., reducing the CSP campaign conformational search space when used as part of structure solution determination.

Given the non-normal profile of the chemical shift histograms, it was important to confirm that the random sampling of *N* = 1000 conformations from the Gaussian probability distributions was sufficiently large to have sampled the distributions well. To do this, the 1000 conformations were randomly sampled using bootstrapping with replacement *M* = 1000 times, given 1000 mean values for each nucleus. These mean values themselves are normally distributed, as can be seen by their close tracking with the identity lines in their Q–Q plots (Figure S13B), indicating that 1000 conformations are sufficient to sample the dynamic 3D structure well. The bootstrap confidence intervals are consistent with those obtained under the CLT (all within 0.1 ppm; see Table S5), indicating that enough conformations have indeed been sampled to come to truly converged values.

### 3.3 Experimental Measurement of Solid-State NMR Chemical Shifts (δ_solid expt_)

Experimental ^13^C and ^1^H chemical shifts for furosemide Form I were taken from Widdifield et al.,^72^ and are given in Table 2. The two molecules in the asymmetric unit of Form I give rise to two distinct sets of chemical shifts that are here treated and reported separately; noting that molecules A and B can be readily distinguished by their torsion 1 values (A ≅ 68°, B ≅ −58°), the assignments are based on ^1^H–^13^C-HETCOR spectra and GIPAW calculations.^72^

To the best of our knowledge, there are currently no published chemical shift values for furosemide Forms II and III.

### 3.4 Calculation of Solid-State NMR Chemical Shifts (δ_solid calc_)

There are both conformational and packing differences between the different forms of furosemide (refer to Introduction, Figures S1 and S2, and Table S1). There are also some lesser variations between the crystal structures within each form, most obviously the problematic placement of hydrogens involved in hydrogen bonds and concomitant effects on the sulfonamide nitrogen sp^2^/sp^3^ geometry as described in the introduction (see Figure S3) and the presence of disorder on the furan ring in Form II at 293 K (FURSEM15) compared to 100 K (FURSEM14). Additionally, noting the different temperatures at which the diffraction experiments were conducted, there is also the expected but more subtle and uneven expansion of unit cells as the temperature is increased (see Table S14B). For example, Form I unit cell parameters change from 100 K (FURSEM13) to 295 K (FURSEM01) as follows: *a* = 9.515 to 9.584 Å (+0.7%); *b* = 10.448 to 10.467 Å (+0.2%); *c* = 15.583 to 15.725 Å (+0.9%), α = 92.84 to 93.47° (+0.63°), β = 107.09 to 107.27° (+0.18°), γ = 116.75 to 115.04 (−1.71°), and volume = 1291.9 to 1332.8 Å^3^ (+3.2%). All of these differences add up to the variations in RMSD_15_ between crystal structures^27^ of the same form shown in Table S1B. For these reasons, chemical shifts were calculated and reported for each crystal structure separately.

Prior to the calculation of chemical shifts, each structure was subjected to DFT geometry optimization of atomic positions with fixed unit cell dimensions. Seeking to preserve the differences between the structures while enabling accurate chemical shift calculations, close attention was paid to any structural changes introduced by the geometry optimization step. Overlays of the asymmetric units of each furosemide form from structures recorded at 100 K before and after geometry optimization are given in Figure S14, showing that only hydrogens noticeably move position. Low RMSD_15_ values between prior and postoptimized coordinates are seen for all structures (see Table S6) except for FURSEM02 and FURSEM17, which is probably due to the problems associated with the carboxylic acid hydrogen noted above.

Torsion angle values for each rotatable bond in furosemide crystal structures before and after geometry optimization are shown in Table S7. Within Form I, the greatest changes are seen in the room-temperature structures FURSEM01, FURSEM17, and FURSEM02 for torsion 6 (refer to Figure 1 for nomenclature) because the sulfonamide geometry is corrected from an sp^2^ hybridization geometry to a more sp^3^-like state as the detail of the hydrogen-bond network around it is modified; this does not, however, change the overall molecular geometry significantly since it effectively corresponds to a partial rotation around torsion 6. The problematic Form I structure FURSEM17 has quite substantial geometry changes involving heavy atoms at torsions 2, 4, and 5 (up to 15°, which therefore changes the overall molecular shape) with large changes at torsion 6 (hybridization state modification and torsion rotation at the sulfonamide group); these adjustments are all consequent effects from the incorrect placement of the carboxylic acid hydrogen, which the DFT geometry optimization does not adjust. FURSEM02, being unlike the other Form I structures in having *Z* = 2 and *Z*′ = 1 (see Section 1), has a single molecule in the Molecule B conformation as assessed by its torsion values (refer to Table S7). Since Molecule A and Molecule B principally differ at torsions 1 and 2, and the Molecule A data is absent in this structural model, it is not surprising that FURSEM02 has its largest changes at torsions 1 and 2 under DFT geometry optimization of 10 and 15°, respectively. Within Form II, the minor disordered conformation at 293 K (25% occupancy, FURSEM15) has some adjustment at torsion 2 after geometry optimization. Form III (FURSEM16, 100 K) does not have any significant changes caused by geometry optimization. Overall, therefore, the DFT geometry optimization step only caused perturbations in structures with known defects.

Using these optimized structures, the solid-state NMR chemical shifts were calculated as detailed in Sections 2.5 and 2.7, providing the values for δ_solid calc_ given in Table 2.

### 3.5 Linear Regression of Δδ_calculated_ vs Δδ_experimental_ and *t*-Test to Identify the Correct Form

The differences in chemical shift between the solution and solid states were determined from the four sets of chemical shifts given in Table 2 according to eqs 1 and 2. For the experimentally measured chemical shift difference of Form I (Δδ_experimental_), separate sets of Δδ values were prepared for each of the two molecules in the asymmetric unit, subtracting the experimental solution-state chemical shifts (δ_solution expt_; see Section 3.1) from the values reported in Widdifield et al.^72^ for the solid-state chemical shifts (δ_solid expt_; see Section 3.3). For the Δδ_calculated_ values, the calculated solution-state chemical shifts (δ_solution calc_; see Section 3.2) were subtracted from the calculated solid-state chemical shifts for each of the crystal structures, again treating the two molecules in Form I crystal structures separately (δ_solid calc_; see Section 3.4). All Δδ_experimental_ and Δδ_calculated_ values for both ^1^H and ^13^C are given in Table 3. As noted previously,^70^ by effectively taking the difference of two calculated chemical shieldings, this methodology does not require absolute referencing of the chemical shifts to be performed.

**Table 3:** Comparison of the Experimentally Measured (Δδ_experimental_) and DFT GIPAW Calculated (Δδ_calculated_) Differences in NMR Chemical Shifts for Furosemide between in Solution and the Solid-State Forms^g^,^h^

	experimentally measured difference (ppm) Δδ_experimental_^a^		calculated difference (ppm) Δδ_calculated_^b^														
	Form I, Molecule^c^		Form I, Molecule											Form II			Form III
nucleus	A	B	13 A^d^	18 A	03 A	01 A	13 B	18 B	03 B	01 B	17 A	17 B	02	14	15 (75%)^e^	15 (25%)	16
C1	–1.75^f^	0.94	–0.18	–0.09	–0.09	0.01	4.00	3.55	3.12	3.24	1.05	1.96	1.99	6.66	4.13	6.18	2.79
C2	1.00	–0.56	4.52	4.51	4.07	3.85	1.37	1.53	1.39	1.79	4.28	1.93	0.13	4.65	6.52	5.27	4.66
C3	1.19	2.35	3.65	3.68	3.53	3.39	6.72	6.32	5.94	6.09	5.61	6.28	6.61	9.06	5.59	7.74	3.41
C4	3.33	–1.27	2.53	2.63	2.58	2.56	–0.33	–0.76	–0.76	–0.55	0.32	–1.28	–2.01	–5.52	–5.78	–5.30	3.51
C5	–0.06	–0.06	0.43	0.65	0.54	0.48	0.40	0.43	0.18	0.32	1.35	–0.02	1.46	2.66	2.82	2.20	–3.17
C6	2.29	1.09	1.76	1.88	1.87	1.92	0.84	1.01	0.86	0.92	1.49	1.09	1.51	–1.45	–1.09	–1.23	1.25
C7	2.69	2.99	3.99	4.39	4.23	4.42	5.68	5.35	4.87	4.67	3.68	4.46	4.06	–0.71	–0.73	–0.31	–0.09
C8	1.30	1.30	–0.54	–1.13	–1.08	–1.02	–1.63	–1.79	–1.65	–1.47	1.32	–2.48	–0.49	3.34	2.94	3.31	–0.32
C9	–2.11	–0.21	–6.02	–5.90	–5.73	–5.54	–3.32	–3.40	–3.52	–3.59	–6.14	–2.96	–4.04	–1.40	–1.40	–0.81	–1.52
C10	2.75	1.51	3.27	3.32	3.00	3.05	1.57	1.62	1.41	1.36	1.98	–1.70	1.60	0.76	1.26	0.82	1.70
C11	–2.63	–0.08	1.77	1.75	1.63	1.42	4.63	4.51	4.10	3.52	1.62	3.96	2.52	1.47	2.31	1.33	3.33
C12	3.09	3.09	3.77	4.01	4.27	4.56	3.97	4.28	4.44	4.65	3.29	0.25	5.01	5.88	6.02	6.18	7.03
H1	–1.20^f^	0.00	–0.70	–0.71	–0.74	–0.73	0.80	0.73	0.64	0.62	–0.71	0.19	0.07	0.70	0.74	0.79	0.08
H2	–0.09	–0.49	0.59	0.59	0.44	0.44	0.10	0.07	0.00	0.01	0.59	0.11	–0.78	0.76	1.48	0.75	0.37
H3	–0.74	–0.44	–0.59	–0.61	–0.61	–0.69	0.25	0.18	0.10	0.11	–0.48	0.04	–0.59	–0.09	–0.02	–0.02	0.41
H4*^g^	0.04	–0.36	0.22	0.25	0.24	0.32	–0.04	–0.04	–0.03	0.09	0.63	–0.16	–0.01	–0.08	–0.37	–0.14	–0.49
H6	–0.31	–0.31	–0.91	–0.94	–0.87	–0.82	–0.54	–0.52	–0.48	–0.36	–1.23	0.53	–0.22	–0.90	–0.73	–0.89	–0.47
H7	0.77	–0.93	0.90	0.87	0.83	0.83	–1.39	–1.41	–1.36	–1.35	0.26	–1.40	0.43	–1.94	–1.48	–1.87	–0.72
H8	0.23	0.13	0.07	0.07	0.05	0.13	–0.01	–0.01	–0.04	0.00	0.06	–0.31	0.11	–0.74	–0.67	–0.67	0.01
H9*^h^	–0.91	–0.71	2.39	2.48	2.51	2.42	3.04	3.07	3.08	3.08	1.48	1.41	–0.02	2.97	2.74	2.73	2.24
H11	–0.66	–0.66	8.46	8.65	8.68	9.34	8.65	8.94	8.90	9.50	3.98	5.38	9.86	8.05	8.44	7.82	8.49

a Difference between experimentally measured solid-state and solution-state NMR chemical shifts as per eq 1 (Δδ_experimental_ = δ_solid exp_ – δ_solution expt_).

b Difference between the GIPAW-calculated solid-state and solution-state NMR chemical shifts as per eq 2 (Δδ_calculated_ = δ_solid calc_ – δ_solution calc_).

c Form I has two molecules in the asymmetric unit, which can be readily distinguished by their torsion 1 values (A ≅ 68°, B ≅ −58°).

d CSD FURSEM entry ID (refer to Table 1).

e FURSEM15 has disorder around the furan ring, occupying two sites at 75 and 25% occupancy, respectively.

f Errors are ^1^H ± 0.2 ppm, ^13^C ± 0.1 ppm.^98,99^

g H4 and H5 have identical chemical shifts in solution due to the absence of a chiral center in the molecule, manifesting as a single resonance, labeled H4*. The solid-state NMR chemical shifts are given as the mean of H4 and H5.

h The sulfonamide hydrogens (H9, H10) are in rapid exchange in solution and manifest in spectra as a single broadened resonance, labeled H9*. The solid-state NMR chemical shifts are given as the mean of H9 and H10.

**Figure 4:**
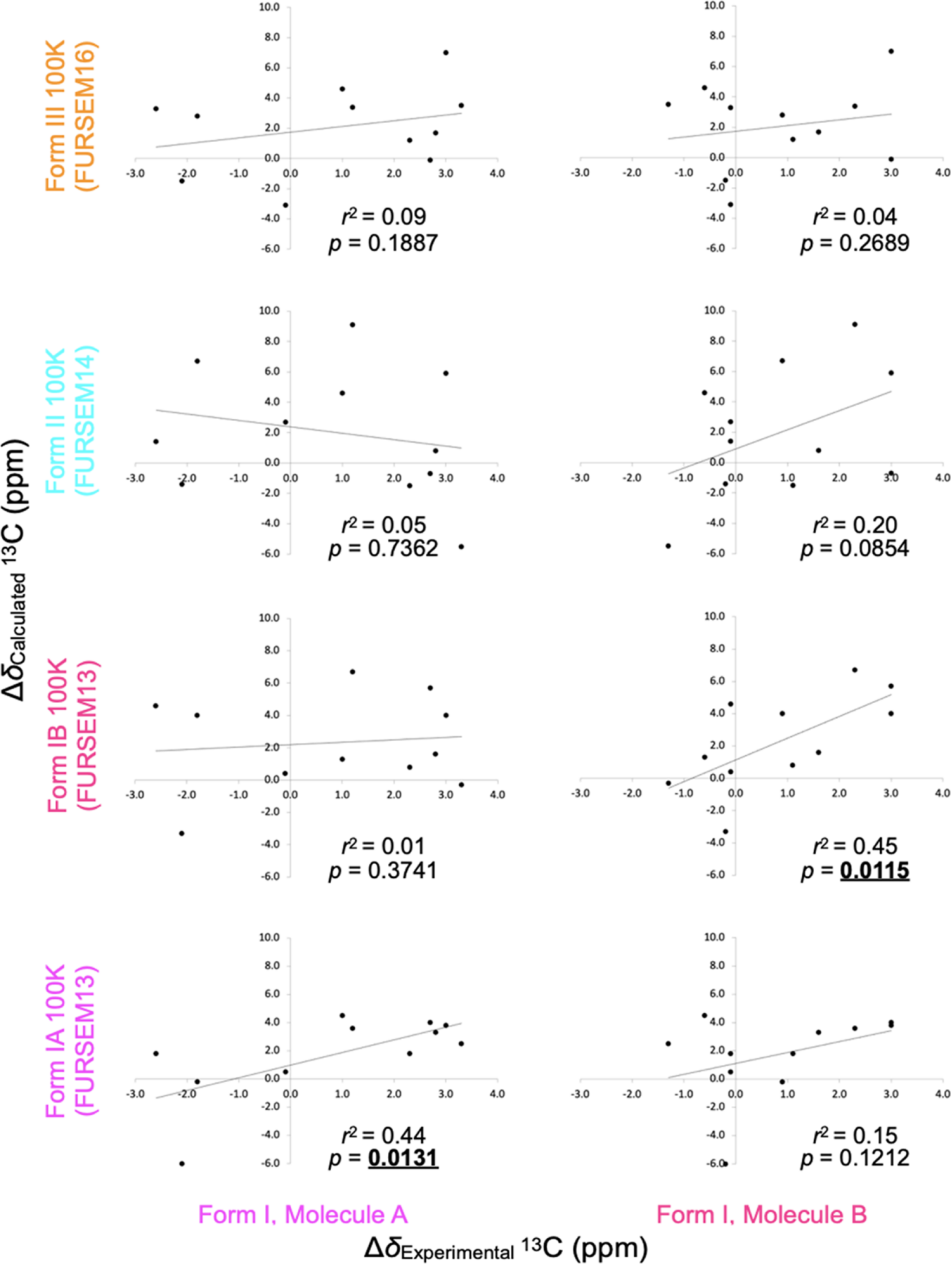
Graph of Δδ_calculated_^13^C data at 100 K for Forms I, II, and III against Δδ_experimental_ for Form I Molecules A and B of furosemide (plotted separately), showing that the approach clearly discriminates Form I molecules from all other forms and each other (Molecule A Δδ_calculated_ against Δδ_experimental_*r*^2^ = 0.44, *p* = 0.0131; Molecule B *r*^2^ = 0.45, *p* = 0.0115). See Tables 3 and 4 for data.

**Figure 5:**
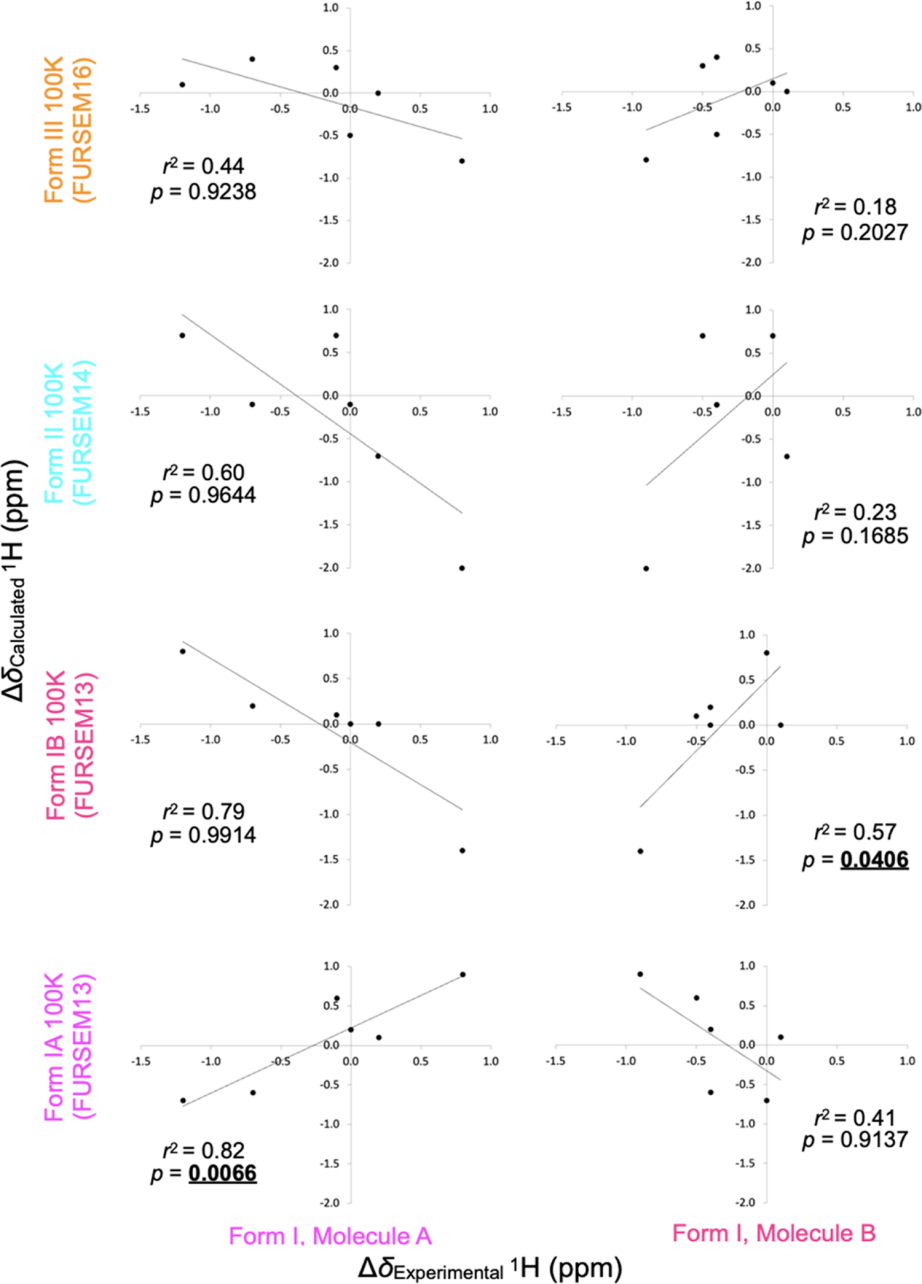
Graph of Δδ_calculated_^1^H data at 100 K for Forms I, II, and III against Δδ_experimental_ for Form I Molecules A and B of furosemide (plotted separately), showing that the approach clearly discriminates Form I molecules from all other forms and each other (Molecule A Δδ_calculated_ against Δδ_experimental_*r*^2^ = 0.82, *p* = 0.0066; Molecule B *r*^2^ = 0.57, *p* = 0.0406). See Tables 3 and 4 for data.

Linear regression was performed on all combinations of Δδ_calculated_ vs Δδ_experimental_ values as described in Section 2.8 to establish whether Form I molecules could be correctly distinguished from those of other forms and each other. All fit parameters are given in Table 4, and plots of Δδ_calculated_ values for crystal structures of Forms I, II, and III collected at 100 K against the Form I Δδ_experimental_ values are shown in Figures 4 and 5 (for ^13^C and ^1^H data, respectively). For each graph, the data was fitted to a simple linear y = *m*x + *c* equation, and the coefficient of the determination value (*r*^2^) was reported (Table 4, Figures 4 and 5). For Form I Molecule A, the ^13^C and ^1^H experimentally measured differences in chemical shift between the solution and the solid state agree well with the calculated differences for all diffraction structures that do not have problems, namely, excluding FURSEM17 and FURSEM02, i.e., for structures that were determined over a wide temperature range (*r*^2^ values of 0.44–0.51 for ^13^C and 0.82–0.87 for ^1^H, with positive gradient *m* values). In contrast, the correlation is poor for both ^13^C and ^1^H for all Forms II and III, and Form I Molecule B for all diffraction structures without defects (*r*^2^ values of 0.00–0.09 for ^13^C and 0.44–0.79 for ^1^H, with negative or weakly positive gradient *m* values). Similarly, the coefficient of determination value (*r*^2^) likewise immediately correctly identifies Form I Molecule B with its experimental data, and clearly distinguishes it from Forms II and III, and Form I Molecule A for all diffraction structures that do not have problems. That is, a simple comparison of *r*^2^ values alone apparently immediately identifies the correct form, although we note that the ^13^C coefficient of determination values are weaker (*r*^2^ approximately 0.5) compared to our previous work on tolfenamic acid^70^ (*r*^2^ approximately 0.8).

**Table 4:** Linear Regression Analysis Parameters and *p*-Values for Chemical Shift Differences between the Solution State and the Solid State for Combinations of Calculated (Forms I, II, and III) and Experimentally Measured (Form I, Molecule A, and Molecule B) Differences in Furosemide NMR Chemical Shift

Δδ_calculated_ for			Δδ_experimental_ for Form I							
			Molecule A^a^				Molecule B^a^			
Form	CSD entry ID	Molecule	*r*^2^^b^	*m*^b^	c^b^	*p*-value	*r*^2^^b^	*m*^b^	c^b^	*p*-value
^13^C										
Form I	13^c^	A	**0.44**^d^	**0.90**	**0.98**	**0.0131**^e^	0.15	0.78	1.08	0.1212
	18	A	**0.46**	**0.92**	**1.08**	**0.0111**	0.16	0.82	1.16	0.1081
	03	A	**0.48**	**0.90**	**1.01**	**0.0094**	0.19	0.85	1.05	0.0923
	01	A	**0.51**	**0.92**	**1.01**	**0.0069**	0.22	0.91	1.02	0.0736
									
	13	B	0.01	0.15	2.19	0.3741	**0.45**	**1.36**	**1.12**	**0.0115**
	18	B	0.02	0.17	2.07	0.3522	**0.49**	**1.37**	**1.00**	**0.0086**
	03	B	0.03	0.21	1.82	0.3144	**0.50**	**1.35**	**0.80**	**0.0074**
	01	B	0.04	0.26	1.81	0.2704	**0.50**	**1.34**	**0.85**	**0.0072**
									
	17	A	**0.22**	**0.64**	**1.11**	**0.0746**	0.24	1.01	0.79	0.0644
	17	B	0.00	–0.09	1.35	0.5793	**0.18**	**0.82**	**0.54**	**0.0942**
									
	02		0.06	0.35	1.41	0.2255	0.64	1.64	0.26	0.0016
Form II	14		0.05	–0.41	2.37	0.7362	0.20	1.29	0.85	0.0854
	15 (75%)^f^		0.04	–0.34	2.09	0.7186	0.13	0.94	0.95	0.1358
	15 (25%)		0.04	–0.36	2.33	0.7256	0.20	1.21	0.93	0.0835
Form III	16		0.09	0.39	1.74	0.1887	0.04	0.41	1.72	0.2689
^1^H										
Form I	13	A	**0.82**	**0.82**	**0.22**	**0.0066**	0.41	–1.06	–0.29	0.9137
	18	A	**0.82**	**0.81**	**0.21**	**0.0068**	0.39	–1.04	–0.29	0.9077
	03	A	**0.86**	**0.80**	**0.17**	**0.0038**	0.40	–1.01	–0.31	0.9109
	01	A	**0.87**	**0.84**	**0.19**	**0.0032**	0.32	–0.94	–0.28	0.8801
									
	13	B	0.79	–0.92	–0.20	0.9914	**0.57**	**1.44**	**0.45**	**0.0406**
	18	B	0.76	–0.88	–0.22	0.9884	**0.60**	**1.44**	**0.42**	**0.0350**
	03	B	0.74	–0.81	–0.25	0.9865	**0.62**	**1.37**	**0.36**	**0.0315**
	01	B	0.70	–0.79	–0.22	0.9813	**0.62**	**1.37**	**0.39**	**0.0314**
									
	17	A	**0.55**	**0.58**	**0.15**	**0.0449**	0.16	–0.57	–0.14	0.7815
	17	B	0.65	–0.68	–0.36	0.9740	**0.40**	**0.98**	**0.09**	**0.0876**
									
	02		0.17	0.29	–0.02	0.2103	0.00	–0.05	–0.08	0.5308
Form II	14		0.60	–1.11	–0.41	0.9644	0.23	1.26	0.21	0.1685
	15 (75%)		0.41	–0.96	–0.21	0.9157	0.09	0.81	0.23	0.2847
	15 (25%)		0.64	–1.12	–0.38	0.9714	0.25	1.29	0.26	0.1586
Form III	16		0.44	–0.43	–0.13	0.9238	0.18	0.51	0.12	0.2027

a Form I has two molecules in the asymmetric unit, which can be readily distinguished by their torsion 1 values (A ≅ 68°, B ≅ −58°). Fit parameters are given for Δδ_calculated_ vs Δδ_experimental_ data for either Molecule A or Molecule B, treated separately.

b Values are for the fit parameters corresponding to the measured experimental data after omitting the chemical shifts for the ^1^H atoms in exchange (H6, H9*, and H11) and the ^13^C atom adjacent to the chlorine (C8). Refer to Figures 4 and 5.

c CSD FURSEM entry ID (refer to Table 1).

d Values in **bold** indicate the fit parameters for the form corresponding to the measured experimental data, i.e., the ones the approach should identify (see Figures 4 and 5).

e *p*-Values are for the null hypothesis that *m* = 0 and the alternative hypothesis *m* > 0. Values underlined reject the null hypothesis at a one-tailed significance level of 0.050, suggesting a significant positive correlation between Δδ_experimental_ and Δδ_calculated_. The lower bound of the one-sided 95% confidence intervals for the correlation between Δδ_experimental_ and Δδ_calculated_ are given in Table S8.

f FURSEM15 has disorder around the furan ring, occupying two sites at 75 and 25% occupancy, respectively.

To establish whether the correlation between Δδ_calculated_ and Δδ_experimental_ is indeed able to discriminate between different crystal forms, a *t*-test was performed with the null hypothesis that *m* = 0 (no correlation) and the alternative hypothesis that *m* > 0 (positive correlation). A one-tailed *t-*test yields a range of *p*-values that extend from the lower bound (reported in Table S8) up to 1, and the null hypothesis is rejected if the *m* = 0 case lies outside the confidence, which is equivalent to *p* < 0.05. At a significance level of *p* = 0.05, the null hypothesis is always correctly rejected for the correct forms (see Tables 4 and S8 for the lower bound of the one-sided 95% confidence intervals). There are some small positive correlations for Δδ_calculated_ data for Forms II and III and Form I Molecule A against Form I Molecule B Δδ_experimental_ data, but their *p*-values are substantially higher than that of the correct Form I Molecule B Δδ_calculated_ data.

It is interesting that the *p*-values for the correctly identified Form I structures progressively improve for both ^13^C and ^1^H as the temperature of the diffraction study increases from 100 to 295 K. As noted above (Section 3.4), the unit cell expands differentially with temperature, and this is reflected in systematic trends for torsion angles 1 and 2 in both Molecules A and B as the temperature increases (refer to Table S7). While torsion 1 for Molecule A progressively approaches the mean value of its corresponding solution mode (mode 2, 62°; refer to Table S4), torsion 1 for Molecule B and torsion 2 for both Molecules A and B progressively depart from the mean values of their corresponding solution modes (mode 1, −62°; mode 1, −91°; mode 2, 91°, respectively). While these trends in torsion values with temperature would appear to be the likely cause of the trend in *p*-values seen with temperature, it is difficult to explain fully how this manifests itself through the regression analysis because there are no immediately obvious corresponding systematic changes with temperature for the calculated chemical shifts for any particular nuclei (refer to Table 2).

The FURSEM17 structure provides fortuitous insight into the sensitivity of the approach, given that it only differs in the placement of a single hydrogen atom in Molecule B (at least before DFT geometry optimization, see Table S7), albeit one that is involved in intermolecular hydrogen bonding (see Section 1 and Figure S3). Molecule A has *p*-values of 0.0746 (^13^C) and 0.0449 (^1^H), which are above and just within the significance threshold, respectively (Table 4). Clearly, the problem associated with Molecule B’s hydrogen atom has some consequent effects for correctly calculating Molecule A’s data, which is reasonable given its torsion angle adjustments seen upon geometry optimization (see Table S7). Nevertheless, the *p*-values for Molecule A are still far lower than the next closest wrong match (^13^C: Form III, *p* = 0.1887; ^1^H: Form II, FURSEM15–75%, *p* = 0.9157). As might be expected, however, Molecule B, which has the incorrect hydrogen, performs much worse, having *p*-values of 0.0942 (^13^C) and 0.0876 (^1^H), which are both above the significance threshold. Moreover, the *p*-values for Molecule B are worse than the next closest wrong matches in the case of ^13^C (^13^C: Form II, FURSEM14, *p* = 0.0854 and FURSEM15–25%, *p* = 0.0835), though they are slightly better in the case of ^1^H (^1^H: Form II, FURSEM15–25%, *p* = 0.1586).

The FURSEM02 structure deviates from the other Form I structures in having a single molecule in its asymmetric unit. As noted above, this molecule has a conformation that is very similar to the Molecule B conformation in the other Form I structures. Indeed, the ^13^C regression data clearly identifies that FURSEM02 has a Molecule B conformation in a suitable packing arrangement with a *p*-value of 0.0016, well below the significance threshold of *p* = 0.05, as compared to a packed Molecule A conformation where *p* = 0.2255 (see Table 4). However, the ^1^H data rank FURSEM02 as the least well-matching to the experimental data for Molecule B (*p* = 0.5308) and intermediate (*p* = 0.2103) between correct Form I Molecule A conformations (*p* < 0.05) and incorrect ones (*p* > 0.9). This clear disparity between a significant and correct ^13^C regression with neither significant nor correct regression for the ^1^H data is a clear indication that this FURSEM02 Form I structure is inconsistent with the solid-state NMR data for Form I, albeit it is at least partly right in many respects (namely, a Molecule B conformation and how it is packed).

Taking the FURSEM17 and FURSEM02 results together, these data suggest that the approach has the potential to identify proposed crystal structures from a CSP campaign that are grossly correct but have errors of detail. With this in mind, crystal structures that give better *p*-values than others could be starting points for iterative refinement of the structure under improving *p*-value, i.e., lending itself to an optimization approach if the initial CSP-generated crystal structure is grossly correct.

It is important to note that the chemical shift difference values for the ^1^H atoms in exchange (H6, H9*, and H11) were omitted from the ^1^H correlation graphs because, as found previously for tolfenamic acid,^70^ the solution chemical shift is strongly affected by solvent exchange in a manner that is not amenable to calculation. Linear regression parameters and *p*-values when all of these ^1^H Δδ values are included are given in Table S9 (with the lower bound of the one-sided 95% confidence intervals specified in Table S10), showing that, under these circumstances, the ^1^H data is no longer able to identify the correct form at all.

In the previous work, it was found that ^13^C nuclei adjacent to chlorine atoms were also problematic for chemical shift calculations and, in the case of tolfenamic acid,^70^ substantially affected the approach’s performance; this follows common practice because atoms bound to heavy atoms are indirectly, but measurably, influenced by relativistic effects.^31^ For consistency with this prior work, the Δδ values for C8 (i.e., the carbon adjacent to the chlorine, see Figure 1) were therefore also omitted in the linear regression and *t*-test analyses shown in Table 4. However, in this case, although the inclusion of C8 did slightly increase the *p*-values, it did not substantially affect the approach’s performance, with all of the correct forms still correctly rejecting the null hypothesis (see Tables S9 and S10).

Finally, linear regression and *t*-test analyses for Δδ_calculated_ against Δδ_experimental_ for Form I when the data from both Molecules A and B are combined together into one graph is given in Table S11. The *p*-values are even stronger in this case, with the trend of decreasing *p-*value with increasing temperature again observed. FURSEM17 passes the *p*-value threshold in both ^1^H and ^13^C, although it is again noticeably worse than all of the other Form I structures. Again, the inclusion of data from C8 is slightly deleterious to the ^13^C correlation in contrast to the inclusion of data for the exchangeable ^1^H nuclei, which causes the approach to fail (see Table S12).

In conclusion, these calculations show that our approach allows ready discrimination of the correct furosemide crystal structure from several other similar structures. The method therefore offers much promise, for example, as a novel general way of ranking trial structures in a CSP study. As noted previously for tolfenamic acid,^70^^13^C again seems to be more discriminating than ^1^H.

### 3.6 Analysis 1: Comparison with the RMSE Method

The *p*-values of this approach (Δδ regression) are compared with the root mean squared error (RMSE) method values in Table 5. The established RMSE method measures how well the experimental solid-state NMR chemical shifts are reproduced by DFT-calculated chemical shifts for the proposed structural model without any additional chemical shift or structural information from solution NMR. The RMSE approach has defined threshold values of 0.33 ppm for ^1^H and 1.9 ppm for ^13^C,^37^ with any structural model that gives lower numbers than the thresholds indicating a positive identification. These thresholds are not absolute values^31^ and are to be taken as ranges with standard deviations of approximately ±0.16 ppm for ^1^H and ±0.4 ppm for ^13^C.^37^

**Table 5:** Comparison of Approaches for Identifying the Correct Form from Solid-State NMR Chemical Shift Data

			Molecule A^a^		Molecule B^a^	
Form	CSD entry ID	Molecule	Δδ regression^b^ (*p*-value)	RMSE^c^ (ppm)	Δδ regression (*p*-value)	RMSE (ppm)
^13^C						
Form I	13^d^	A	**0.0131**^e^^,^^f^	**1.85**^e^^,^^g^	0.1212	2.43
	18	A	**0.0111**	**1.69**	0.1081	2.32
	03	A	**0.0094**	**1.70**	0.0923	2.27
	01	A	**0.0069**	**1.71**	0.0736	2.25
					
	13	B	0.3741	3.00	**0.0115**	**1.85**
	18	B	0.3522	2.88	**0.0086**	**1.69**
	03	B	0.3144	2.84	**0.0074**	**1.71**
	01	B	0.2704	2.80	**0.0072**	**1.77**
					
	17	A	**0.0746**	**2.56**	**0.0644**	**2.54**
	17	B	0.5793	3.10	**0.0942**	**2.15**
					
	02		0.2255	2.95	0.0016	1.95
Form II	14		0.7362	5.34	0.0854	4.37
	15 (75%)^h^		0.7186	4.83	0.1358	3.99
	15 (25%)		0.7256	5.17	0.0835	4.23
Form III	16		0.1887	3.16	0.2689	2.88
^1^H						
Form I	13^d^	A	**0.0066**^e^^,^^f^	**0.34**^e^^,^^g^	0.9134	0.71
	18	A	**0.0069**	**0.38**	0.9077	0.72
	03	A	**0.0038**	**0.37**	0.9109	0.70
	01	A	**0.0032**	**0.44**	0.8801	0.78
					
	13	B	0.9916	1.18	**0.0406**	**0.48**
	18	B	0.9884	1.19	**0.0350**	**0.49**
	03	B	0.9865	1.16	**0.0319**	**0.47**
	01	B	0.9813	1.20	**0.0314**	**0.52**
					
	17	A	**0.0449**	**0.52**	0.7815	0.61
	17	B	0.9740	1.12	**0.0876**	**0.80**
					
	02		0.2103	0.74	0.5308	0.61
Form II	14		0.9644	1.31	0.1685	0.75
	15 (75%)^h^		0.9157	1.29	0.2847	0.82
	15 (25%)		0.9714	1.27	0.1586	0.70
Form III	16		0.9238	0.90	0.2027	0.40

a Form I has two molecules in the asymmetric unit, which can be readily distinguished by their torsion 1 values (A ≅ 68°, B ≅ – 58°). Fit parameters are given for Δδ_calculated_ vs Δδ_experimental_ data for either Molecule A or Molecule B, treated separately.

b The approach in this work.

c The RMSE approach.^37^

d CSD FURSEM entry ID (refer to Table 1).

e Values in **bold** indicate the fit parameters for the form corresponding to the measured experimental data, i.e., the ones the approaches should identify.

f *p*-Values are for the null hypothesis that *m* = 0 and the alternative hypothesis *m* > 0. Values underlined reject the null hypothesis at a one-tailed significance level of 0.050, suggesting a significant positive correlation between Δδ_experimental_ and Δδ_calculated_. Data as in Table 4.

g RMSE values between δ_solid expt_ and δ_solid calc_ as calculated according to ref (37). Note that in this approach, no ^1^H or ^13^C data points are removed due to exchange with solvent or other reasons. Values underlined indicate RMSE values that are within the thresholds for identifying a correct match (^13^C 1.9 ppm, ^1^H 0.33 ppm).^37^ See Table S13 for linear regression parameters.

h FURSEM15 has disorder around the furan ring, occupying two sites at 75 and 25% occupancy, respectively.

Table 5 shows that none of the furosemide crystal structures meet the RMSE threshold for ^1^H, although all Form I Molecule A and some of the Form 1 Molecule B calculated data vs their experimental data lie within one standard deviation of the threshold (0.33 + 0.16 = 0.49 ppm). Moreover, the ^1^H RMSE value for Form III against Molecule B data (0.40) is lower than that for any of the correct Form I Molecule B structures (0.48–0.52), which would therefore incorrectly suggest this is the best match. Nevertheless, it is generally true that lower RMSE values are attained for Form I Molecule A and Molecule B compared to the other forms and each other, and they do suggest that there is a problem with FURSEM17 Molecule B (perhaps only with the benefit of hindsight though). As with the Δδ regression approach, the RMSE method correctly does not identify FURSEM02 as being a good match with the Form I experimental data. Overall, it is concluded that the RMSE analysis would not definitively identify the correct furosemide crystal structure based on the ^1^H chemical shifts, although perhaps the use of different chemical shift reference values or not fixing the *m* coefficient during regression (aspects of the RMSE approach that are still debated in the literature^34,62,91,93,99^) may redeem it in this case. In contrast, as shown in Section 3.5 above, the *p-*values of the Δδ regression approach unambiguously and clearly identify the correct structures throughout and indicate that FURSEM17 molecules are largely right but have some errors of detail.

RMSE analysis of the ^13^C chemical shifts, however, correctly identifies all of the correct Form I structures under the threshold (1.9 ppm), and none of the incorrect structures pass the threshold nor lie within its one standard deviation (1.9 + 0.4 = 2.3 ppm). The problematic FURSEM17 structure also fails to pass the test, although in this case Molecule B (which is more incorrect structurally) scores better than Molecule A, being within one standard deviation of the threshold. FURSEM02 is also suggested to be like Form I Molecule B, with a value of 1.95 being just outside the threshold.

It is somewhat surprising that, in this case, RMSE analysis correctly identifies all of the correct Form I structures from ^13^C chemical shifts but not ^1^H chemical shifts because Baias et al.^34^ previously found that ^1^H chemical shifts are more sensitive to crystal structure. The explanation for this result is not immediately apparent.

A first significant point of difference between the methods is, therefore, that while the RMSE method quantifies the agreement of the calculated chemical shifts with the experimental solid-state NMR values, it does not provide a probability that the calculated and experimental results are in agreement,^31^ in contrast to the Δδ regression approach, which gives a well-defined statistical measure of the probability of agreement. Second, the Δδ regression approach is able to easily discriminate forms using ^1^H data, while the RMSE method is not, at least in this case. Third, and most significantly, it is noteworthy that the dynamic ranges offered by the two approaches are very different. The difference in *p*-values obtained for proposing the correct molecule in the correct form vs an incorrect proposal with the Δδ regression approach is typically a factor of 5–10-fold different (^1^H: worst correct 0.0406 vs best incorrect 0.1685, 4.2× fold; ^13^C worst correct 0.0131 vs best incorrect 0.0854, 6.5× fold), in contrast to that of the RMSE method, which shows only a small variation of approximately 1-fold different (^1^H: worst correct 0.52 vs best incorrect 0.40, 0.75× fold; ^13^C: worst correct 1.85 vs best incorrect 2.25, 1.2× fold). Clearly, greater discriminating power is afforded by the Δδ regression approach in using the change in chemical shift on passing from solution to solid rather than relying on the reproduction of chemical shifts from the solid state alone. As exemplified by the FURSEM17 structure, this greater dynamic range appears to allow the identification of structures that are essentially correct but retain some minor incorrect features, which is more difficult with the narrower dynamic range of the RMSE method. This again suggests that the Δδ regression approach has promise for iterative searching and optimization approaches in finding the best match predicted crystal structure from a CSP campaign.

Unlike the *p*-values of the Δδ regression approach that systematically improve for both ^13^C and ^1^H data as the temperature of the crystal structure determination increases, the RMSE values for ^13^C systematically improve while those for the^1^H data systematically worsen as the temperature increases (Table 5). While these trends again probably arise from the differential expansion of the unit cell and concomitant systematic torsion angle changes at torsions 1 and 2 with temperature, it is difficult to explain fully how this effect is transmitted through the RMSE method into the result seen. Whether these temperature effects on the two methods’ output are somewhat peculiar to furosemide or will be generally observed is also not determinable from the furosemide data alone presented in this work.

### 3.7 Analysis 2: Effects of Allowing Unit Cell Parameters to Vary during Geometry Optimization

The trends with temperature noted above prompted an analysis of the effects of allowing the unit cell to vary during the geometry optimization step. To achieve this, DFT-D dispersion correction was implemented according to the approach of Tkatchenko & Scheffler^90^ as described in Section 2.5. Calculated densities and crystal structure energies relative to Form I at 100 K (FURSEM13) after geometry optimization are compared for fixed unit cell parameters and variable unit cell parameters during geometry optimization in Table S14. As would be expected, the trends in crystal structure energy and density observed within each form under optimization with fixed unit cell parameters are essentially smoothed out in the optimization with variable unit cell parameters as the structures approach convergence to a common end-point. Supporting this, the differences in crystal packing similarity (as assessed by RMSD_15_) between the Form I structures with temperature are also largely smoothed out to a common residual value of <0.2 (Table S15), aligning closely with the residual values of 95% of the structures previously reported by Sacchi et al.^27^ The crystal structure energy of FURSEM17 still stands out after geometry optimization with variable unit cell parameters, consistent with its problematic carboxylic acid hydrogen position. FURSEM02 has a conspicuously large change in crystal structure energy between fixed and variable unit cell parameter calculations (from +77.6 to +9.67 kJ/mol, relative to the energy of FURSEM13), similarly indicating that this structure has problematic aspects as noted above.

The GIPAW calculated chemical shifts for each solid form from these variable unit cell parameter calculations^44^ (i.e., alternative values for δ_solid calc_) are given in Table S16 and the corresponding Δδ_calculated_ values in Table S17. Linear regression analysis parameters and *p*-values using these alternative variable unit cell Δδ_calculated_ values are given in Table S18, and the lower bound of the one-sided 95% confidence intervals for the *t*-test are given in Table S19.

The use of variable unit cell parameters during geometry optimization neither significantly improves nor worsens the results from the Δδ regression approach for either ^13^C or ^1^H data, with comparable numbers of *p*-values being slightly better or slightly worse than those shown in Table 5. FURSEM17 is more obviously correct at Molecule A and problematic at Molecule B with variable unit cell parameters during geometry optimization, but the changes are small and perhaps merely fortuitous. As would be expected, the subtle trends in *p*-value with temperature noted above with fixed unit cell parameters during geometry optimization (see Section 3.5 and Table 4) are no longer apparent with variable unit cell parameters during geometry optimization (Table S18). Therefore, the Δδ regression approach neither requires nor is hindered by geometry optimization with variable unit cell parameters, performing equally well with crystal structures determined at different temperatures, without or with DFT-D geometry optimization of the unit cell parameters.

Linear regression analysis parameters for the RMSE method using these δ_solid calc_ values from variable unit cell parameters during geometry optimization are given in Table S20 and are compared to the results from the Δδ regression approach in Table S21. Overall, the RMSE method generally has worse results for both ^13^C and ^1^H data (6 correct matches improve, 14 worsen) with variable unit cell parameters during geometry optimization, but again the differences are quite small. FURSEM02, however, does now dramatically pass the threshold for matching Molecule B ^13^C experiment data, having a better RMSE value than any of the more correct Form I structures; on the one hand, this is good because the variable unit cell geometry optimization process has produced a more viable structural match to the experimental data, but on the other hand this result could be strongly misleading. Again, there are no trends with temperature remaining. Overall, at least in this case of furosemide, the RMSE method appears to perform somewhat better with fixed unit cell parameters during geometry optimization.

### 3.8 Analysis 3: Choice of Solvent and Charge State for Solution Data

Our approach uses the experimentally measured change in chemical shift in passing from solution (δ_solution expt_) to the solid state (δ_solid expt_). This change involves different contributions, including not only those from conformation (i.e., from a conformational ensemble in solution to one or a few conformations in the solid) and molecular packing in the crystal lattice, but also desolvation, and possibly charge-transfer (in the case of salt formation). Though the desolvation process and change of charge state are of less interest to identifying the correct solid form, here the choices of solvent and charge state were investigated to determine their impact on the success of the approach, as was done previously for tolfenamic acid.^70^

The sensitivity of the dynamic 3D structure of furosemide to solvent was first assessed in a more aqueous environment (80% D_2_O, 20% DMSO-*d*_6_) in both neutral (pH 2.11) and negatively charged states (pH 6.77). Neither condition measurably perturbed the dynamic 3D structure in solution from that measured in pure DMSO-*d*_6_, as determined by comparing distances from NOE data measured under different solvent conditions.

Nevertheless, changes in solvent and charge state do induce chemical shift changes, which affect the values that could be used for δ_solution expt_. ^1^H and ^13^C chemical shifts for furosemide in solution under the three different solvent conditions explored in this work are shown in Table S22. After re-referencing the raw data to the absolute chemical shift scale (i.e., to TMS in CDCl_3_ at 0 ppm), the changes in chemical shift caused by changing from pure DMSO-*d*_6_ to the more aqueous environment can be calculated for both the carboxylic acid state (i.e., neutral) and also for the deprotonated carboxylate (i.e., charged state, see Table S22, Figure S15). While most changes in chemical shift when the solvent is changed from pure DMSO-*d*_6_ to the more aqueous environment (80% D_2_O, 20% DMSO-*d*_6_) are small (^13^C < 0.5 ppm, ^1^H < 0.2 ppm), there is a single large change at C9 of 2.85 ppm. As expected, when the charge is changed, some changes in chemical shift are substantial (^13^C > 2.0 ppm), most evidently at C8, C9, C11, and C12.

The linear regression analysis was first repeated using these alternative δ_solution expt_ values for neutral furosemide in an aqueous environment (80% D_2_O, 20% DMSO-*d*_6_, observed pH 2.11) (data given in Tables S23–S25). As shown in Table 6, the approach has maintained its performance for ^1^H, with all of the correct structures being identified, and none of the incorrect ones, and FURSEM17 Molecule B again being highlighted as essentially correct but slightly problematic (*p* = 0.0587). The *p*-values for ^13^C are however noticeably worse, with none of the correct matches passing the significance test although still clearly distinguishing them from incorrect ones in most cases (FURSEM14 and FURSEM15 ^13^C data give comparable *p*-values for Molecule B). Thus, in contrast to the previous results for tolfenamic acid, the choice of solvent for measuring the solution-state chemical shifts (δ_solution expt_) is of importance for the approach’s accuracy with ^13^C data. With the main difference in the data being the chemical shift of C9 noted above, it is clear that this one data point is having a strong effect on the end result. With hindsight, this is not surprising since C9 is immediately adjacent to the highly polar sulfonamide group, which will interact very differently with DMSO-*d*_6_ compared to water with its hydrogen-bond donating ability. To be aware of such possible deleterious effects caused by solvent choice, it is recommended to measure ^13^C data in a couple of solvents to see which nuclei’s chemical shifts are particularly sensitive to solvent effects and treat such nuclei cautiously in the regression analysis. Additionally, it seems prudent to choose solvents that share similar physical characteristics to the molecule of interest in order to better match the solid-state packed environment. In this case, DMSO-*d*_6_ with its S=O group was a sensible choice to match the sulfonamide S=O groups in furosemide. Nevertheless, since ^1^H chemical shifts are, in general, much less sensitive to solvent effects than ^13^C, this points out a clear advantage of using ^1^H data alongside ^13^C data.

**Table 6:** *p*-Values on Passing from Solution to the Solid State for Combinations of Calculated (Forms I, II, III) and Experimentally Measured (Form I, Molecule A and Molecule B) Changes in Furosemide Using Solution-State Chemical Shift Data from Different Sample Conditions

Δδ_calculated_ for			*p*-values for Δδ_experimental_ for Form I^a^					
			Molecule A			Molecule B^b^		
Form	CSD entry ID	Molecule	neutral DMSO^c^^,^^d^	neutral aqueous^e^	charged aqueous^f^	neutral DMSO^c^^,^^d^	neutral aqueous^e^	charged aqueous^f^
^13^C								
Form I	13^g^	A	**0.0131**^h^^,^^i^^,^^j^	**0.1274**	**0.0047**	0.1212	0.6850	0.0007
	18	A	**0.0111**	**0.1175**	**0.0046**	0.1081	0.6626	0.0006
	03	A	**0.0094**	**0.1087**	**0.0047**	0.0923	0.6313	0.0006
	01	A	**0.0069**	**0.0932**	**0.0047**	0.0736	0.5873	0.0006
							
	13	B	0.3741	0.6993	0.0354	**0.0115**	**0.1569**	**0.0007**
	18	B	0.3522	0.6912	0.0354	**0.0086**	**0.1542**	**0.0008**
	03	B	0.3144	0.6532	0.0329	**0.0074**	**0.1513**	**0.0007**
	01	B	0.2704	0.6044	0.0292	**0.0072**	**0.1578**	**0.0007**
							
	17	A	**0.0746**	**0.3297**	**0.0114**	0.0644	0.5329	0.0007
	17	B	0.5793	0.8183	0.0305	**0.0942**	**0.3019**	**0.0004**
							
	02		0.2255	0.5495	0.0297	0.0016	0.0963	0.0006
Form II	14		0.7362	0.8769	0.0905	0.0854	0.1562	0.0047
	15 (75%)^k^		0.7186	0.8784	0.0860	0.1358	0.2700	0.0062
	15 (25%)		0.7256	0.8654	0.0818	0.0835	0.1487	0.0042
Form III	16		0.1887	0.3129	0.0196	0.2689	0.5374	0.0027
^1^H								
Form I	13^c^	A	**0.0066**	**0.0051**	**0.0083**	0.9134	0.9355	0.9453
	18	A	**0.0068**	**0.0050**	**0.0078**	0.9077	0.9298	0.9418
	03	A	**0.0038**	**0.0025**	**0.0050**	0.9109	0.9343	0.9435
	01	A	**0.0032**	**0.0018**	**0.0030**	0.8801	0.9078	0.9211
							
	13	B	0.9914	0.9893	0.9961	**0.0406**	**0.0203**	**0.0596**
	18	B	0.9884	0.9858	0.9941	**0.0350**	**0.0168**	**0.0508**
	03	B	0.9865	0.9832	0.9930	**0.0315**	**0.0142**	**0.0445**
	01	B	0.9813	0.9771	0.9890	**0.0314**	**0.0144**	**0.0420**
							
	17	A	**0.0449**	**0.0362**	**0.0206**	0.7815	0.8017	0.8814
	17	B	0.9740	0.9714	0.9842	**0.0876**	**0.0587**	**0.1472**
							
	02		0.2103	0.1858	0.1207	0.5308	0.5311	0.6129
Form II	14		0.9644	0.9563	0.9649	0.1685	0.1116	0.2146
	15 (75%)^k^		0.9157	0.9076	0.9084	0.2847	0.2222	0.3657
	15 (25%)		0.9714	0.9647	0.9724	0.1586	0.1036	0.2043
Form III	16		0.9238	0.9382	0.9055	0.2027	0.2002	0.4254

a Linear regression analysis parameters for these data are given in Table 4 (“Neutral DMSO”), Table S24 (“Neutral aqueous”) and Table S28 (“Charged aqueous”). The lower bound of the one-sided 95% confidence intervals for the correlation between Δδ_experimental_ and Δδ_calculated_ are given in Tables S8, S25, and S29, respectively.

b Form I has two molecules in the asymmetric unit, which can be readily distinguished by their torsion 1 values (A ≅ 68°, B ≅ −58°). *p*-Values are given for Δδ_calculated_ vs Δδ_experimental_ data for either Molecule A or Molecule B, treated separately.

c See also Tables 4 and 5.

d Sample conditions are 10 mM furosemide in 100% DMSO-*d*_6_, under which the carboxylic acid group is neutral.

e Sample conditions are 2.5 mM furosemide in 80:20 (v/v) mixture of D_2_O/DMSO-*d*_6_, observed pH 2.11, under which the carboxylic acid group is neutral (p*K*_a_ 4.11 ± 0.05).

f Sample conditions are 2.5 mM furosemide in 80:20 (v/v) mixture of D_2_O/DMSO-*d*_6_, observed pH 6.77, under which the carboxylic acid group is charged (p*K*_a_ 4.11 ± 0.05).

g CSD FURSEM entry ID (refer to Table 1).

h Values are for the *p*-values for linear regression of data omitting the chemical shifts for the ^1^H atoms in exchange (H6, H9*, and H11) and the ^13^C atom adjacent to the chlorine (C8).

i Values in **bold** indicate the fit parameters for the form corresponding to the measured experimental data, i.e., the ones the approach should identify.

j *p*-values are for the null hypothesis that *m* = 0 and the alternative hypothesis *m* > 0. Values underlined reject the null hypothesis at a one-tailed significance level of 0.050, suggesting a significant positive correlation between Δδ_calculated_ and Δδ_experimental_.

k FURSEM15 has disorder around the furan ring, occupying two sites at 75 and 25% occupancy, respectively.

The linear regression analysis was then repeated using the alternative δ_solution expt_ values for charged furosemide in an aqueous environment (80% D_2_O, 20% DMSO-*d*_6_, observed pH 6.77) (data given in Tables S27–S29). Calculated chemical shifts for charged furosemide in solution (δ_solution calc_) were calculated following the same process as for the neutral molecule but starting from a deprotonated base conformation (see Section 2.6 and Table S26). The approach has again maintained its performance for ^1^H (which had relatively small chemical shift changes caused by the change of charge state; Figure S15 and Table S22), however the ^13^C data now no longer discriminates between any forms, returning significant *p*-values in nearly all cases. Regression analysis is highly sensitive to extreme values and, in this case, the change in charge state gives an extreme Δδ_calculated_ value for C11 of 7.67 ppm, giving this data point in particular high leverage and undue influence, driving the correlation to false positives. Thus, as found previously for tolfenamic acid,^70^ matching the charge state between the solution and the solid states is of high importance, and especially so for ^13^C data. This preference to correctly match the charge state is not prohibitive in the practical application of the approach, however, because the solid-state chemical shifts are, in most cases, immediately diagnostic for the molecule’s charge state within the crystal, allowing solution conditions to be chosen to reproduce that same charge state prior to solution chemical shift measurements. If, however, it were possible to only measure solution experimental data with a mismatched charge state, an alternative statistical analysis such as Bayesian model selection^24^ might be more appropriate and successful.

### 3.9 Analysis 4: Approximation of Solution Dynamic 3D Structure Using a Substitute Ensemble of Furosemide Conformations from the CSD

Having demonstrated that the approach can be successfully applied to flexible molecules, we sought to broaden its usability by critiquing the dependence on the solution dynamic 3D structure for calculating δ_solution calc_. In particular, we investigated whether the large collection of furosemide single-crystal diffraction structures in the neutral state in the CSD (also comprising neutral solvates and cocrystals) could provide a substitute ensemble that might mimic the solution-state behavior well enough.

**Figure 6:**
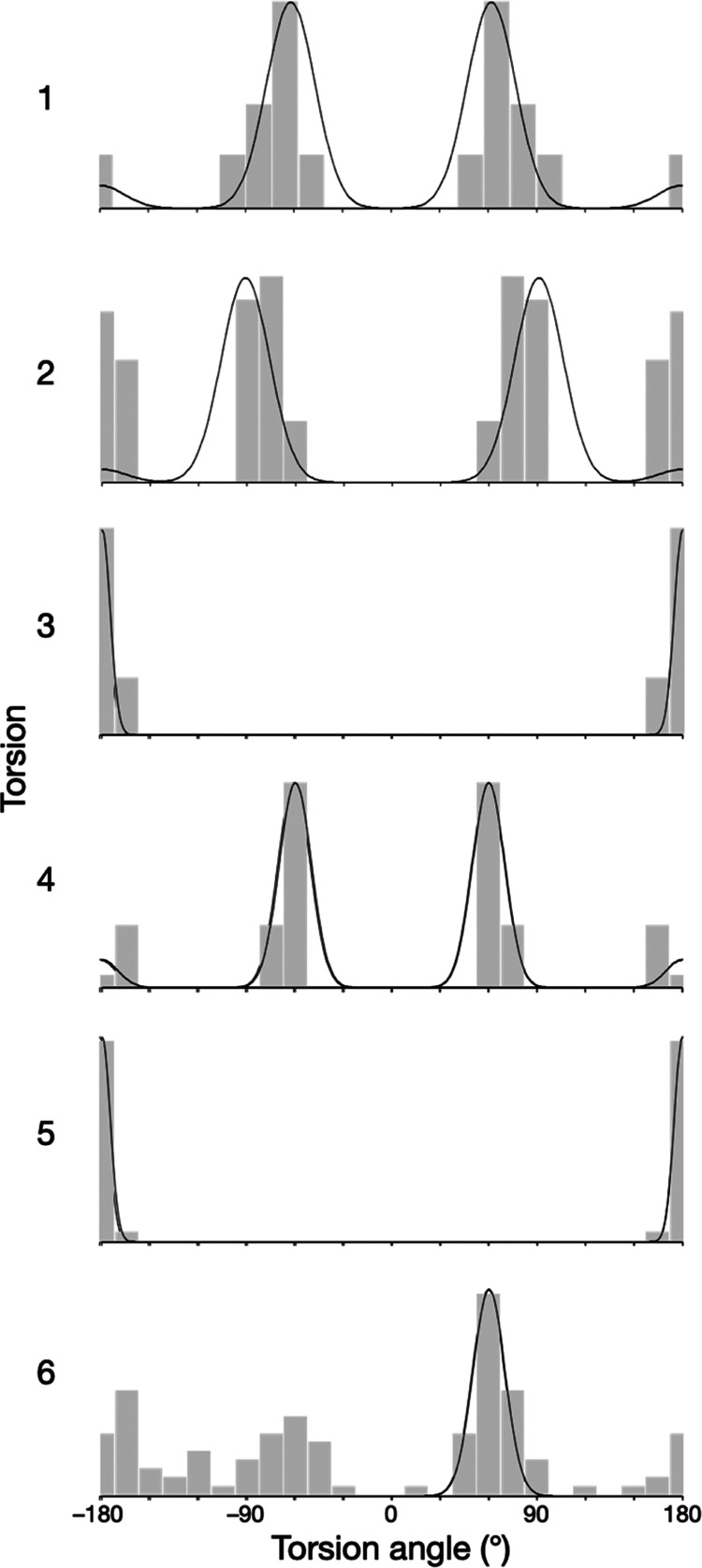
Histograms of torsion values from all crystal structures in the CSD containing neutral furosemide (also comprising neutral solvates and cocrystals, bars) compared to the solution dynamic 3D structure (lines). The histograms comprise data from 45 single-crystal diffraction structures, constituting 108 conformations (see the text and Table S30). For each torsion (see Figure 1 for definitions), the changing population with torsion angle (*x*-axis) is given relative to its maximum occupancy (*y*-axis).

There are 45 crystal structures containing neutral furosemide in the CSD (version 5.41), giving 54 distinct conformations across all of their asymmetric units (9 structures are *Z*′ = 2). Together, this produces an overall ensemble of 108 neutral furosemide conformations when all of the mirror images are included. The torsion values of each conformation were extracted (see Table S30), and these values were applied to the base conformation to create a substitute ensemble for estimating δ_solution calc_ (see Section 2.6). In gross appearance, this “CSD-SX” ensemble is quite similar to that of the measured solution dynamic 3D structure (see Figure S16). Histograms of the torsion values compared to the solution dynamic 3D structure population line-graphs for each torsion are given in Figure 6, showing that there is a good correspondence between the behavior of furosemide in solution and this collection of single-crystal structures considered as a whole. Torsion 6, however, has a noticeably more diverse behavior in the solid state compared to solution, which is most likely because it is usually involved in hydrogen-bonding interactions which would compensate energetically for the adoption of more diverse conformations.

Calculated δ_solution calc_ values for this substitute ensemble are given in Table S31. While there are some moderately large differences in calculated ^13^C values (up to −1.7 ppm), the differences in calculated ^1^H values are relatively small (most are 0.1–0.2 ppm; maximum 0.4 ppm). None of these differences obviously localize to any part of the molecule (Figure S17).

The linear regression analysis was then repeated using these alternative δ_solution calc_ values (Tables S32 and S33). As shown in Table 7, the approach has maintained its performance extremely well, with all of the correct structures being identified and none of the incorrect ones. The FURSEM17 Molecule B still shows a higher *p*-value, indicating that it is problematic but essentially correct.

**Table 7:** *p*-Values on Passing from Solution to the Solid State for Combinations of Calculated (Forms I, II, III) and Experimentally Measured (Form I, Molecule A and Molecule B) Changes in Furosemide Using a Substitute Ensemble from CSD Single-Crystal Diffraction Structures (SX) to Calculate δ_solution calc_

Δδ_calculated_ for			*p*-values for Δδ_experimental_ for Form I			
			Molecule A^a^		Molecule B^a^	
Form	CSD entry ID	Molecule	neutral DMSO^b^	CSD-SX^c^	neutral DMSO^b^	CSD-SX^c^
^13^C						
Form I	13^d^	A	**0.0131**^e^^,^^f^^,^^g^	**0.0096**^h^	0.1212	0.2398
	18	A	**0.0111**	**0.0110**	0.1081	0.2321
	03	A	**0.0094**	**0.0094**	0.0923	0.2105
	01	A	**0.0069**	**0.0072**	0.0736	0.1807
					
	13	B	0.3741	0.4159	**0.0115**	**0.0456**
	18	B	0.3522	0.3935	**0.0086**	**0.0400**
	03	B	0.3144	0.3478	**0.0074**	**0.0346**
	01	B	0.2704	0.2951	**0.0072**	**0.0327**
					
	17	A	**0.0746**	**0.0349**	0.0644	0.0927
	17	B	0.5793	0.6414	**0.0942**	**0.2232**
					
	02		0.2255	0.1793	0.0016	0.0005
Form II	14		0.7362	0.7900	0.0854	0.0991
	15 (75%)^i^		0.7186	0.7701	0.1358	0.1826
	15 (25%)		0.7256	0.7782	0.0835	0.1035
Form III	16		0.1887	0.2229	0.2689	0.4626
^1^H						
Form I	13^c^	A	**0.0066**	**0.0056**	0.9134	0.9347
	18	A	**0.0068**	**0.0057**	0.9077	0.9306
	03	A	**0.0038**	**0.0039**	0.9109	0.9319
	01	A	**0.0032**	**0.0031**	0.8801	0.9085
					
	13	B	0.9914	0.9933	**0.0406**	**0.0472**
	18	B	0.9884	0.9901	**0.0350**	**0.0402**
	03	B	0.9865	0.9873	**0.0315**	**0.0371**
	01	B	0.9813	0.9804	**0.0314**	**0.0379**
					
	17	A	**0.0449**	**0.0229**	0.7815	0.8539
	17	B	0.9740	0.9732	**0.0876**	**0.1143**
					
	02		0.2103	0.1535	0.5308	0.6531
Form II	14		0.9644	0.9573	0.1685	0.2051
	15 (75%)^i^		0.9157	0.8956	0.2847	0.3416
	15 (25%)		0.9714	0.9664	0.1586	0.1930
Form III	16		0.9238	0.8974	0.2027	0.3073

a Form I has two molecules in the asymmetric unit, which can be readily distinguished by their torsion 1 values (A ≅ 68°, B ≅ −58°). *p*-Values are given for Δδ_calculated_ vs Δδ_experimental_ data for either Molecule A or Molecule B, treated separately.

b See also Tables 4 and 5.

c Results for the substitute solution ensemble made from the collection of conformations of neutral furosemide in the CSD.

d CSD FURSEM entry ID (refer to Table 1).

e Values are for the *p*-values for linear regression of data omitting the chemical shifts for the ^1^H atoms in exchange (H6, H9*, and H11) and the ^13^C atom adjacent to the chlorine (C8).

f Values in **bold** indicate the fit parameters for the form corresponding to the measured experimental data, i.e., the ones the approach should identify.

g *p*-Values are for the null hypothesis that *m* = 0 and the alternative hypothesis *m* > 0. Values underlined reject the null hypothesis at a one-tailed significance level of 0.050, suggesting a significant positive correlation between Δδ_calculated_ and Δδ_experimental_.

h Linear regression analysis parameters for these data are given in Table S32. The lower bound of the one-sided 95% confidence intervals for the correlation between Δδ_experimental_ and Δδ_calculated_ are given in Table S33. Here δ_solution calc_ is replaced with values from Table S31 for the ensemble of neutral furosemide conformations extracted from the CSD.

i FURSEM15 has disorder around the furan ring, occupying two sites at 75 and 25% occupancy, respectively.

Thus, at least in the case of furosemide, the solution conformational behavior can be adequately approximately with a sufficiently large number of crystal conformations in the same charge state, suggesting that CSD data may be used more generally to create an ensemble of conformations suitable for estimating δ_solution calc_ values.

## 4 Conclusions

In this work, we have modified the Δδ regression approach described in a study by Blade et al.^70^ to be suitable for molecules with multiple conformational degrees of freedom and exemplified this improvement on furosemide, a typical pharmaceutical molecule with 6 rotatable bonds. The modification presented here is the use of Monte Carlo random sampling of the solution dynamic 3D structure to create a representative ensemble of conformations for calculating the solution-state chemical shift (δ_solution calc_). Rather than millions of conformations that the previous systematic sampling would have required, this modification means that sample sizes of even just 500 members are adequate for calculating δ_solution calc_ with sufficient precision for use in the linear regression analysis. Even though furosemide has inherently less data points available (12 for ^13^C, 6 for ^1^H) compared to tolfenamic acid (14 for ^13^C, 9 for ^1^H) and 5 more rotatable bonds, the Δδ regression approach has still been successful, accurate, and precise. The approach is also sufficiently sensitive to indicate which structures are essentially correct but have minor problems. It works equally well with unit cell parameters being fixed or variable during geometry optimization, making it impervious to the temperature conditions of crystal structure measurement.

Provided the solution dynamic 3D structure does not change with solvent, the choice of solvent for measuring δ_solution expt_ has again been found to be relatively unimportant for ^1^H data but now, in this study, more important for ^13^C data. In particular, the charge state in solution should match that in the crystal structure being compared against. This is not a problematic caveat because the charge state in the solid form being studied is usually immediately evident from the solid-state NMR chemical shifts, meaning that a solvent system and pH value can be easily chosen accordingly. Additionally, ^13^C chemical shifts that are particularly sensitive to changes in solvent should be treated with caution in the regression analysis, and it is prudent to measure data in several solvents to determine which nuclei (if any) behave so. A choice of solvent with physical properties similar to those of the molecule of interest is also likely to be beneficial for results.

We have also demonstrated that the approach is differently sensitive to the popular RMSE method, allowing correct structures to be readily identified from ^1^H data when the RMSE method gives suggestive but indecisive results. We observe that the Δδ regression approach provides a much greater dynamic range in its “scoring” parameter (i.e., *p*-value vs RMSE), allowing the identification of structures that are essentially correct but with some minor incorrect features, which is obscured by the narrower dynamic range of the RMSE method. We note that the RMSE method can have success with incomplete or ambiguous chemical shift assignments,^34^ which has not yet been explored for the Δδ regression approach.

The Δδ regression approach is, however, more labor intensive than the RMSE method, with the generation of the solution dynamic 3D structures for calculating δ_solution expt_ being demanding. While the Monte Carlo random sampling has dramatically reduced the time and cost required to calculate the solution-state chemical shifts compared to that required under the systematic sampling of Blade et al.,^70^ the cost and time of performing the required DFT calculations are significant, and would be expected to be more so for larger molecules with more rotatable bonds; in this regard, recent advances in the rapid calculation of accurate chemical shifts through machine learning^25,103−105^ may ameliorate this potential obstacle. Additionally, in the case of furosemide, the solution conformational ensemble can be well approximated by an ensemble of all of the conformations from the large collection of single crystals of furosemide in the same charge state (45 structures) as the solid-state NMR data displays. Few molecules will, of course, have such a wealth of prior-existing crystal data, but this insight is nevertheless suggestive that averaged data from the CSD where there are few or no crystal structures of the molecule under investigation may provide alternative routes to estimating δ_solution expt_ sufficiently well to be useful in the Δδ regression approach. On the other hand, it also seems unlikely that solution dynamic 3D structures will generally be mirrored quite so well by aggregated CSD data, as was observed in this particular case of furosemide.

In terms of future outlook, these results suggest several applications for the approach in solving structures by NMR crystallography combined with focused CSP campaigns to generate trial structures. First, we suggest that the most helpful conformations for input into CSP calculations are those selected from within the solution dynamic 3D structure, potentially greatly reducing the conformational space searching burden. In principle, low-energy conformations in solution are likely to be similar to the low-energy conformations when packed in the solid state, especially for molecules of rather limited hydrogen-bonding and salt-bridging capabilities, as pharmaceutical molecules typically are. Second, conformations from the solution ensemble whose calculated chemical shifts at key conformationally sensitive nuclei best match those of the solid-state NMR data should be prioritized for input into CSP calculations for structure determination activities. Third, having run a CSP campaign, the Δδ regression approach can be used to rank potential crystal structures, with *p*-values determining when the correct crystal structure is found. Fourth, *p*-values could potentially be used in an iterative improvement loop, taking structures that are essentially grossly correct but with incorrect details and refining them against the NMR chemical shift data until the best-fitting structure is found.

## Supporting Information Available

Comparison of conformations from each polymorph of furosemide; FURSEM17 Form I structure has been solved with one carboxylic acid hydrogen in the wrong orientation; experimentally measured conformational parameter values for the solution dynamic 3D-structure of furosemide; Crystal packing similarity of furosemide crystal structures after DFT geometry optimization; solution-state NMR chemical shifts for furosemide (δ_solution expt_) under different solvent conditions; numerical data from this study are provided as a supporting data set from the University of Warwick Research Data sets portal at http://wrap.warwick.ac.uk/179773 (PDF)

## References

[ref1] Desiraju G. R. Crystal Engineering: From Molecule to Crystal. J. Am. Chem. Soc. 2013, 135 (27), 9952–9967. 10.1021/ja403264c. 23750552

[ref2] Bhardwaj R. M.; McMahon J. A.; Nyman J.; Price L. S.; Konar S.; Oswald I. D. H.; Pulham C. R.; Price S. L.; Reutzel-Edens S. M. A Prolific Solvate Former, Galunisertib, under the Pressure of Crystal Structure Prediction, Produces Ten Diverse Polymorphs. J. Am. Chem. Soc. 2019, 141 (35), 13887–13897. 10.1021/jacs.9b06634. 31394896

[ref3] Mortazavi M.; Hoja J.; Aerts L.; Quéré L.; van de Streek J.; Neumann M. A.; Tkatchenko A. Computational polymorph screening reveals late-appearing and poorly-soluble form of rotigotine. Commun. Chem. 2019, 2 (1), 70 10.1038/s42004-019-0171-y.

[ref4] Hoja J.; Ko H.-Y.; Neumann M. A.; Car R.; DiStasio R. A.; Tkatchenko A. Reliable and practical computational description of molecular crystal polymorphs. Sci. Adv. 2019, 5 (1), eaau3338 10.1126/sciadv.aau3338. 30746448 PMC6357866

[ref5] Greenwell C.; McKinley J. L.; Zhang P.; Zeng Q.; Sun G.; Li B.; Wen S.; Beran G. J. O. Overcoming the difficulties of predicting conformational polymorph energetics in molecular crystals via correlated wavefunction methods. Chem. Sci. 2020, 11 (8), 2200–2214. 10.1039/C9SC05689K. 32190277 PMC7059316

[ref6] Beran G. J. O.; Sugden I. J.; Greenwell C.; Bowskill D. H.; Pantelides C. C.; Adjiman C. S. How many more polymorphs of ROY remain undiscovered. Chem. Sci. 2022, 13 (5), 1288–1297. 10.1039/D1SC06074K. 35222912 PMC8809489

[ref7] Cerreia Vioglio P.; Mollica G.; Juramy M.; Hughes C. E.; Williams P. A.; Ziarelli F.; Viel S.; Thureau P.; Harris K. D. M. Insights into the Crystallization and Structural Evolution of Glycine Dihydrate by In Situ Solid-State NMR Spectroscopy. Angew. Chem., Int. Ed. 2018, 57 (22), 6619–6623. 10.1002/anie.201801114. 29633439

[ref8] Al Rahal O.; Hughes C. E.; Williams P. A.; Logsdail A. J.; Diskin-Posner Y.; Harris K. D. M. Polymorphism of l-Tryptophan. Angew. Chem., Int. Ed. 2019, 58 (52), 18788–18792. 10.1002/anie.201908247. 31621998

[ref9] Smalley C. J. H.; Hoskyns H. E.; Hughes C. E.; Johnstone D. N.; Willhammar T.; Young M. T.; Pickard C. J.; Logsdail A. J.; Midgley P. A.; Harris K. D. M. A structure determination protocol based on combined analysis of 3D-ED data, powder XRD data, solid-state NMR data and DFT-D calculations reveals the structure of a new polymorph of l-tyrosine. Chem. Sci. 2022, 13 (18), 5277–5288. 10.1039/D1SC06467C. 35655549 PMC9093151

[ref10] LeBlanc L. M.; Dale S. G.; Taylor C. R.; Becke A. D.; Day G. M.; Johnson E. R. Pervasive Delocalisation Error Causes Spurious Proton Transfer in Organic Acid–Base Co-Crystals. Angew. Chem., Int. Ed. 2018, 57 (45), 14906–14910. 10.1002/anie.201809381. 30248221

[ref11] Widdifield C. M.; Farrell J. D.; Cole J. C.; Howard J. A. K.; Hodgkinson P. Resolving alternative organic crystal structures using density functional theory and NMR chemical shifts. Chem. Sci. 2020, 11 (11), 2987–2992. 10.1039/C9SC04964A. 34122800 PMC8157514

[ref12] Li M.; Xu W.; Su Y. Solid-state NMR spectroscopy in pharmaceutical sciences. Trac. Trends Anal. Chem. 2021, 135, 116152 10.1016/j.trac.2020.116152.

[ref13] Štoček J. R.; Socha O.; Císařová I.; Slanina T.; Dračínský M. Importance of Nuclear Quantum Effects for Molecular Cocrystals with Short Hydrogen Bonds. J. Am. Chem. Soc. 2022, 144 (16), 7111–7116. 10.1021/jacs.1c10885. 35394771

[ref14] Brown S. P. Applications of high-resolution ^1^H solid-state NMR. Solid State Nucl. Magn. Reson. 2012, 41, 1–27. 10.1016/j.ssnmr.2011.11.006. 22177472

[ref15] Thureau P.; Carvin I.; Ziarelli F.; Viel S.; Mollica G. A Karplus Equation for the Conformational Analysis of Organic Molecular Crystals. Angew. Chem., Int. Ed. 2019, 58 (45), 16047–16051. 10.1002/anie.201906359. 31397043

[ref16] Struppe J.; Quinn C. M.; Sarkar S.; Gronenborn A. M.; Polenova T. Ultrafast ^1^H MAS NMR Crystallography for Natural Abundance Pharmaceutical Compounds. Mol. Pharmaceutics 2020, 17 (2), 674–682. 10.1021/acs.molpharmaceut.9b01157. PMC7307729 31891271

[ref17] Cordova M.; Balodis M.; Hofstetter A.; Paruzzo F.; Nilsson Lill S. O.; Eriksson E. S. E.; Berruyer P.; Simões de Almeida B.; Quayle M. J.; Norberg S. T.; Svensk Ankarberg A.; Schantz S.; Emsley L. Structure determination of an amorphous drug through large-scale NMR predictions. Nat. Commun. 2021, 12 (1), 2964 10.1038/s41467-021-23208-7. 34016980 PMC8137699

[ref18] Evans C. L.; Evans I. R.; Hodgkinson P. Resolving alternative structure determinations of indapamide using ^13^C solid-state NMR. Chem. Commun. 2022, 58 (30), 4767–4770. 10.1039/D1CC06256E. 35343549

[ref19] Cordova M.; Emsley L. Chemical Shift-Dependent Interaction Maps in Molecular Solids. J. Am. Chem. Soc. 2023, 145 (29), 16109–16117. 10.1021/jacs.3c04538. 37440302 PMC10375520

[ref20] Taylor C. R.; Mulvee M. T.; Perenyi D. S.; Probert M. R.; Day G. M.; Steed J. W. Minimizing Polymorphic Risk through Cooperative Computational and Experimental Exploration. J. Am. Chem. Soc. 2020, 142 (39), 16668–16680. 10.1021/jacs.0c06749. 32897065 PMC7586337

[ref21] Yang S.; Day G. M. Global analysis of the energy landscapes of molecular crystal structures by applying the threshold algorithm. Commun. Chem. 2022, 5 (1), 86 10.1038/s42004-022-00705-4. 36697680 PMC9814927

[ref22] Musil F.; De S.; Yang J.; Campbell J. E.; Day G. M.; Ceriotti M. Machine learning for the structure–energy–property landscapes of molecular crystals. Chem. Sci. 2018, 9 (5), 1289–1300. 10.1039/C7SC04665K. 29675175 PMC5887104

[ref23] Paruzzo F. M.; Hofstetter A.; Musil F.; De S.; Ceriotti M.; Emsley L. Chemical shifts in molecular solids by machine learning. Nat. Commun. 2018, 9 (1), 4501 10.1038/s41467-018-06972-x. 30374021 PMC6206069

[ref24] Cordova M.; Balodis M.; Almeida B. S. d.; Ceriotti M.; Emsley L. Bayesian probabilistic assignment of chemical shifts in organic solids. Sci. Adv. 2021, 7 (48), eabk2341 10.1126/sciadv.abk2341. 34826232 PMC8626066

[ref25] Balodis M.; Cordova M.; Hofstetter A.; Day G. M.; Emsley L. De Novo Crystal Structure Determination from Machine Learned Chemical Shifts. J. Am. Chem. Soc. 2022, 144 (16), 7215–7223. 10.1021/jacs.1c13733. 35416661 PMC9052749

[ref26] Hofstetter A.; Balodis M.; Paruzzo F. M.; Widdifield C. M.; Stevanato G.; Pinon A. C.; Bygrave P. J.; Day G. M.; Emsley L. Rapid Structure Determination of Molecular Solids Using Chemical Shifts Directed by Unambiguous Prior Constraints. J. Am. Chem. Soc. 2019, 141 (42), 16624–16634. 10.1021/jacs.9b03908. 31117663 PMC7540916

[ref27] Sacchi P.; Lusi M.; Cruz-Cabeza A. J.; Nauha E.; Bernstein J. Same or different – that is the question: identification of crystal forms from crystal structure data. CrystEngComm 2020, 22 (43), 7170–7185. 10.1039/D0CE00724B.

[ref28] Elena B.; Emsley L. Powder Crystallography by Proton Solid-State NMR Spectroscopy. J. Am. Chem. Soc. 2005, 127 (25), 9140–9146. 10.1021/ja051208t. 15969592

[ref29] Elena B.; Pintacuda G.; Mifsud N.; Emsley L. Molecular Structure Determination in Powders by NMR Crystallography from Proton Spin Diffusion. J. Am. Chem. Soc. 2006, 128 (29), 9555–9560. 10.1021/ja062353p. 16848494

[ref30] Bryce D. L. NMR crystallography: structure and properties of materials from solid-state nuclear magnetic resonance observables. IUCRJ. 2017, 4 (4), 350–359. 10.1107/S2052252517006042. 28875022 PMC5571798

[ref31] Hodgkinson P. NMR Crystallography of Molecular Organics. Prog. Nucl. Magn. Reson. Spectrosc. 2020, 118–119, 10–53. 10.1016/j.pnmrs.2020.03.001. 32883448

[ref32] Berendt R. T.; Sperger D. M.; Munson E. J.; Isbester P. K. Solid-state NMR spectroscopy in pharmaceutical research and analysis. TrAC, Trends Anal. Chem. 2006, 25 (10), 977–984. 10.1016/j.trac.2006.07.006.

[ref33] Zilka M.; Yates J. R.; Brown S. P. An NMR crystallography investigation of furosemide. Magn. Reson. Chem. 2019, 57 (5), 191–199. 10.1002/mrc.4789. 30141257 PMC6492277

[ref34] Baias M.; Widdifield C. M.; Dumez J.-N.; Thompson H. P. G.; Cooper T. G.; Salager E.; Bassil S.; Stein R. S.; Lesage A.; Day G. M.; Emsley L. Powder crystallography of pharmaceutical materials by combined crystal structure prediction and solid-state ^1^H NMR spectroscopy. Phys. Chem. Chem. Phys. 2013, 15 (21), 8069–8080. 10.1039/c3cp41095a. 23503809

[ref35] Harris R. K.; Cadars S.; Emsley L.; Yates J. R.; Pickard C. J.; Jetti R. K. R.; Griesser U. J. NMR crystallography of oxybuprocaine hydrochloride, Modification II°. Phys. Chem. Chem. Phys. 2007, 9 (3), 360–368. 10.1039/B614318K. 17199152

[ref36] Tatton A. S.; Blade H.; Brown S. P.; Hodgkinson P.; Hughes L. P.; Nilsson Lill S. O.; Yates J. R. Improving Confidence in Crystal Structure Solutions Using NMR Crystallography: The Case of β-Piroxicam. Cryst. Growth. Des. 2018, 18 (6), 3339–3351. 10.1021/acs.cgd.8b00022.

[ref37] Salager E.; Day G. M.; Stein R. S.; Pickard C. J.; Elena B.; Emsley L. Powder crystallography by combined crystal structure prediction and high-resolution ^1^H solid-state NMR spectroscopy. J. Am. Chem. Soc. 2010, 132 (8), 2564–2566. 10.1021/ja909449k. 20136091

[ref38] Hofstetter A.; Emsley L. Positional Variance in NMR Crystallography. J. Am. Chem. Soc. 2017, 139 (7), 2573–2576. 10.1021/jacs.6b12705. 28146348

[ref39] Ding F.; Griffith K. J.; Koçer C. P.; Saballos R. J.; Wang Y.; Zhang C.; Nisbet M. L.; Morris A. J.; Rondinelli J. M.; Poeppelmeier K. R. Multimodal Structure Solution with ^19^F NMR Crystallography of Spin Singlet Molybdenum Oxyfluorides. J. Am. Chem. Soc. 2020, 142 (28), 12288–12298. 10.1021/jacs.0c04019. 32530621

[ref40] Caulkins B. G.; Young R. P.; Kudla R. A.; Yang C.; Bittbauer T. J.; Bastin B.; Hilario E.; Fan L.; Marsella M. J.; Dunn M. F.; Mueller L. J. NMR Crystallography of a Carbanionic Intermediate in Tryptophan Synthase: Chemical Structure, Tautomerization, and Reaction Specificity. J. Am. Chem. Soc. 2016, 138 (46), 15214–15226. 10.1021/jacs.6b08937. 27779384 PMC5129030

[ref41] Al-Ani A. J.; Szell P. M. J.; Rehman Z.; Blade H.; Wheatcroft H. P.; Hughes L. P.; Brown S. P.; Wilson C. C. Combining X-ray and NMR Crystallography to Explore the Crystallographic Disorder in Salbutamol Oxalate. Cryst. Growth. Des. 2022, 22 (8), 4696–4707. 10.1021/acs.cgd.1c01093. PMC9374327 35971412

[ref42] Ashbrook S. E.; Dawson D. M.; Gan Z.; Hooper J. E.; Hung I.; Macfarlane L. E.; McKay D.; McLeod L. K.; Walton R. I. Application of NMR Crystallography to Highly Disordered Templated Materials: Extensive Local Structural Disorder in the Gallophosphate GaPO-34A. Inorg. Chem. 2020, 59 (16), 11616–11626. 10.1021/acs.inorgchem.0c01450. 32799506

[ref43] Rehman Z.; Franks W. T.; Nguyen B.; Schmidt H. F.; Scrivens G.; Brown S. P. Discovering the Solid-State Secrets of Lorlatinib by NMR Crystallography: To Hydrogen Bond or not to Hydrogen Bond. J. Pharm. Sci. 2023, 112 (7), 1915–1928. 10.1016/j.xphs.2023.02.022. 36868358

[ref44] Dudenko D. V.; Yates J. R.; Harris K. D. M.; Brown S. P. An NMR crystallography DFT-D approach to analyse the role of intermolecular hydrogen bonding and π–π interactions in driving cocrystallisation of indomethacin and nicotinamide. CrystEngComm 2013, 15 (43), 8797–8807. 10.1039/c3ce41240g.

[ref45] Watts A. E.; Maruyoshi K.; Hughes C. E.; Brown S. P.; Harris K. D. M. Combining the Advantages of Powder X-ray Diffraction and NMR Crystallography in Structure Determination of the Pharmaceutical Material Cimetidine Hydrochloride. Cryst. Growth. Des. 2016, 16 (4), 1798–1804. 10.1021/acs.cgd.6b00016.

[ref46] Neumann M. A.; Perrin M.-A. Energy Ranking of Molecular Crystals Using Density Functional Theory Calculations and an Empirical van der Waals Correction. J. Phys. Chem. B 2005, 109 (32), 15531–15541. 10.1021/jp050121r. 16852970

[ref47] Neumann M. A.; Streek J. v. d.; Fabbiani F. P. A.; Hidber P.; Grassmann O. Combined crystal structure prediction and high-pressure crystallization in rational pharmaceutical polymorph screening. Nat. Commun. 2015, 6 (1), 7793 10.1038/ncomms8793. 26198974 PMC4525153

[ref48] Kendrick J.; Leusen F. J. J.; Neumann M. A.; Streek J. v. d. Progress in Crystal Structure Prediction. Chem. - Eur. J. 2011, 17 (38), 10736–10744. 10.1002/chem.201100689. 22003515

[ref49] Nyman J.; Day G. M. Static and lattice vibrational energy differences between polymorphs. CrystEngComm 2015, 17 (28), 5154–5165. 10.1039/C5CE00045A.

[ref50] Nyman J.; Yu L.; Reutzel-Edens S. M. Accuracy and reproducibility in crystal structure prediction: the curious case of ROY. CrystEngComm 2019, 21 (13), 2080–2088. 10.1039/C8CE01902A.

[ref51] Kazantsev A. V.; Karamertzanis P.; Pantelides C.; Adjiman C. CrystalOptimizer: An Efficient Algorithm for Lattice Energy Minimization of Organic Crystals Using Isolated-Molecule Quantum Mechanical Calculations, In Process Systems Engineering: Volume 6: Molecular Systems Engineering, 2010; pp 1–42.

[ref52] Day G. M. Current approaches to predicting molecular organic crystal structures. Crystallogr. Rev. 2011, 17 (1), 3–52. 10.1080/0889311X.2010.517526.

[ref53] Ryan K.; Lengyel J.; Shatruk M. Crystal Structure Prediction via Deep Learning. J. Am. Chem. Soc. 2018, 140 (32), 10158–10168. 10.1021/jacs.8b03913. 29874459

[ref54] Price S. L.; Braun D. E.; Reutzel-Edens S. M. Can computed crystal energy landscapes help understand pharmaceutical solids?. Chem. Commun. 2016, 52 (44), 7065–7077. 10.1039/C6CC00721J. PMC5486446 27067116

[ref55] Price S. L. Why don’t we find more polymorphs?. Acta Crystallogr., Sect. B: Struct. Sci., Cryst. Eng. Mater. 2013, 69 (4), 313–328. 10.1107/S2052519213018861. 23873056

[ref56] Price S. L. Predicting crystal structures of organic compounds. Chem. Soc. Rev. 2014, 43 (7), 2098–2111. 10.1039/C3CS60279F. 24263977

[ref57] Price S. L. The computational prediction of pharmaceutical crystal structures and polymorphism. Adv. Drug Deliver Rev. 2004, 56 (3), 301–319. 10.1016/j.addr.2003.10.006. 14962583

[ref58] Brus J.; Czernek J.; Kobera L.; Urbanova M.; Abbrent S.; Husak M. Predicting the Crystal Structure of Decitabine by Powder NMR Crystallography: Influence of Long-Range Molecular Packing Symmetry on NMR Parameters. Cryst. Growth. Des. 2016, 16 (12), 7102–7111. 10.1021/acs.cgd.6b01341.

[ref59] Dudek M. K.; Paluch P.; Śniechowska J.; Nartowski K. P.; Day G. M.; Potrzebowski M. J. Crystal structure determination of an elusive methanol solvate – hydrate of catechin using crystal structure prediction and NMR crystallography. CrystEngComm 2020, 22 (30), 4969–4981. 10.1039/D0CE00452A.

[ref60] Bravetti F.; Bordignon S.; Alig E.; Eisenbeil D.; Fink L.; Nervi C.; Gobetto R.; Schmidt M. U.; Chierotti M. R. Solid-State NMR-Driven Crystal Structure Prediction of Molecular Crystals: The Case of Mebendazole. Chem. - Eur. J. 2022, 28 (6), e202103589 10.1002/chem.202103589. 34962330

[ref61] Khalaji M.; Paluch P.; Potrzebowski M. J.; Dudek M. K. Narrowing down the conformational space with solid-state NMR in crystal structure prediction of linezolid cocrystals. Solid State Nucl. Magn. Reson. 2022, 121, 101813 10.1016/j.ssnmr.2022.101813. 35964358

[ref62] Hartman J. D.; Kudla R. A.; Day G. M.; Mueller L. J.; Beran G. J. O. Benchmark fragment-based ^1^H, ^13^C, ^15^N and ^17^O chemical shift predictions in molecular crystals. Phys. Chem. Chem. Phys. 2016, 18 (31), 21686–21709. 10.1039/C6CP01831A. 27431490 PMC4991946

[ref63] Nilsson Lill S. O.; Widdifield C. M.; Pettersen A.; Svensk Ankarberg A.; Lindkvist M.; Aldred P.; Gracin S.; Shankland N.; Shankland K.; Schantz S.; Emsley L. Elucidating an Amorphous Form Stabilization Mechanism for Tenapanor Hydrochloride: Crystal Structure Analysis Using X-ray Diffraction, NMR Crystallography, and Molecular Modeling. Mol. Pharmaceutics 2018, 15 (4), 1476–1487. 10.1021/acs.molpharmaceut.7b01047. 29490140

[ref64] Pickard C. J.; Mauri F. All-electron magnetic response with pseudopotentials: NMR chemical shifts. Phys. Rev. B 2001, 63 (24), 245101 10.1103/PhysRevB.63.245101.

[ref65] Yates J. R.; Pickard C. J.; Mauri F. Calculation of NMR chemical shifts for extended systems using ultrasoft pseudopotentials. Phys. Rev. B 2007, 76 (2), 024401 10.1103/PhysRevB.76.024401.

[ref66] Bonhomme C.; Gervais C.; Babonneau F.; Coelho C.; Pourpoint F.; Azaïs T.; Ashbrook S. E.; Griffin J. M.; Yates J. R.; Mauri F.; Pickard C. J. First-Principles Calculation of NMR Parameters Using the Gauge Including Projector Augmented Wave Method: A Chemist’s Point of View. Chem. Rev. 2012, 112 (11), 5733–5779. 10.1021/cr300108a. 23113537

[ref67] Engel E. A.; Anelli A.; Hofstetter A.; Paruzzo F.; Emsley L.; Ceriotti M. A Bayesian approach to NMR crystal structure determination. Phys. Chem. Chem. Phys. 2019, 21 (42), 23385–23400. 10.1039/C9CP04489B. 31631196

[ref68] Oganov A. R. Crystal structure prediction: reflections on present status and challenges. Faraday Discuss. 2018, 211 (0), 643–660. 10.1039/C8FD90033G. 30306975

[ref69] Cruz-Cabeza A. J.; Reutzel-Edens S. M.; Bernstein J. Facts and fictions about polymorphism. Chem. Soc. Rev. 2015, 44 (23), 8619–8635. 10.1039/C5CS00227C. 26400501

[ref70] Blade H.; Blundell C. D.; Brown S. P.; Carson J.; Dannatt H. R. W.; Hughes L. P.; Menakath A. K. Conformations in Solution and in Solid-State Polymorphs: Correlating Experimental and Calculated Nuclear Magnetic Resonance Chemical Shifts for Tolfenamic Acid. J. Phys. Chem. A 2020, 124 (43), 8959–8977. 10.1021/acs.jpca.0c07000. 32946236

[ref71] Cruz-Cabeza A. J.; Bernstein J. Conformational Polymorphism. Chem. Rev. 2014, 114 (4), 2170–2191. 10.1021/cr400249d. 24350653

[ref72] Widdifield C. M.; Robson H.; Hodgkinson P. Furosemide’s one little hydrogen atom: NMR crystallography structure verification of powdered molecular organics. Chem. Commun. 2016, 52 (40), 6685–6688. 10.1039/C6CC02171A. 27115483

[ref73] Sarojini B. K.; Yathirajan H. S.; Narayana B.; Sunil K.; Bolte M. CSD Communication Private Communication 2007.

[ref74] Fronckowiak M.; Hauptmann H. American Crystallographic Association, Abstracts Papers Winter 1976, 9.

[ref75] Babu N. J.; Cherukuvada S.; Thakuria R.; Nangia A. Conformational and Synthon Polymorphism in Furosemide (Lasix). Cryst. Growth. Des. 2010, 10 (4), 1979–1989. 10.1021/cg100098z.

[ref76] Lamotte J.; Campsteyn H.; Dupont L.; Vermeire M. Structure cristalline et moléculaire de l’acide furfurylamino-2 chloro-4 sulfamoyl-5 benzoïque, la furosémide (C_12_H_11_ClN_2_O_5_S). Acta Crystallogr. B: Struct. Sci. 1978, 34 (5), 1657–1661. 10.1107/S0567740878006251.

[ref77] Bolukbasi O.; Yilmaz A. X-ray structure analysis and vibrational spectra of Furosemide. Vib. Spectrosc. 2012, 62, 42–49. 10.1016/j.vibspec.2012.06.002.

[ref78] Shin W.; Jeon G. S. The Crystal Structure of Furosemide. Proc. Coll. Natur. Sci., SNU 1983, 8, 45–51.

[ref79] Macrae C. F.; Bruno I. J.; Chisholm J. A.; Edgington P. R.; McCabe P.; Pidcock E.; Rodriguez-Monge L.; Taylor R.; van de Streek J.; Wood P. A. Mercury CSD 2.0 - new features for the visualization and investigation of crystal structures. J. Appl. Crystallogr. 2008, 41, 466–470. 10.1107/S0021889807067908.

[ref80] Harris R. K.; Becker E. D.; Menezes S. M. C. d.; Granger P.; Hoffman R. E.; Zilm K. W. Further conventions for NMR shielding and chemical shifts (IUPAC Recommendations 2008). Pure Appl. Chem. 2008, 80 (1), 59–84. 10.1351/pac200880010059. 18407566

[ref81] Hoffman R. E. Standardization of chemical shifts of TMS and solvent signals in NMR solvents. Magn. Reson. Chem. 2006, 44, 606–616. 10.1002/mrc.1801. 16534833

[ref82] Blundell C. D.; Packer M. J.; Almond A. Quantification of free ligand conformational preferences by NMR and their relationship to the bioactive conformation. Bioorg. Med. Chem. 2013, 21 (17), 4976–4987. 10.1016/j.bmc.2013.06.056. 23886813 PMC3744816

[ref83] Thiele C. M.; Petzold K.; Schleucher J. EASY ROESY: Reliable Cross-Peak Integration in Adiabatic Symmetrized ROESY. Chem. - Eur. J. 2009, 15 (3), 585–588. 10.1002/chem.200802027. 19065697

[ref84] Clark S. J.; Segall M. D.; Pickard C. J.; Hasnip P. J.; Probert M. I. J.; Refson K.; Payne M. C. First principles methods using CASTEP. Z. Kristallogr. - Cryst. Mater. 2005, 220 (5–6), 567–570. 10.1524/zkri.220.5.567.65075.

[ref85] Perdew J. P.; Burke K.; Ernzerhof M. Generalized Gradient Approximation Made Simple. Phys. Rev. Lett. 1996, 77 (18), 3865–3868. 10.1103/PhysRevLett.77.3865. 10062328

[ref86] Lejaeghere K.; Bihlmayer G.; Björkman T.; Blaha P.; Blügel S.; Blum V.; Caliste D.; Castelli I. E.; Clark S. J.; Dal Corso A.; de Gironcoli S.; Deutsch T.; Dewhurst J. K.; Di Marco I.; Draxl C.; Dułak M.; Eriksson O.; Flores-Livas J. A.; Garrity K. F.; Genovese L.; Giannozzi P.; Giantomassi M.; Goedecker S.; Gonze X.; Grånäs O.; Gross E. K.; Gulans A.; Gygi F.; Hamann D. R.; Hasnip P. J.; Holzwarth N. A.; Iuşan D.; Jochym D. B.; Jollet F.; Jones D.; Kresse G.; Koepernik K.; Küçükbenli E.; Kvashnin Y. O.; Locht I. L.; Lubeck S.; Marsman M.; Marzari N.; Nitzsche U.; Nordström L.; Ozaki T.; Paulatto L.; Pickard C. J.; Poelmans W.; Probert M. I.; Refson K.; Richter M.; Rignanese G. M.; Saha S.; Scheffler M.; Schlipf M.; Schwarz K.; Sharma S.; Tavazza F.; Thunström P.; Tkatchenko A.; Torrent M.; Vanderbilt D.; van Setten M. J.; Van Speybroeck V.; Wills J. M.; Yates J. R.; Zhang G. X.; Cottenier S. Reproducibility in density functional theory calculations of solids. Science 2016, 351 (6280), aad3000 10.1126/science.aad3000. 27013736

[ref87] Tatton A. S.; Hughes L.; Blade H.; Nilsson Lill S. O.; Bartók-Pártay A.; Brown S. P.; Hodgkinson P. Isolated molecule calculation protocol with Materials Studio. https://www.ccpnc.ac.uk/output/isolated-molecule-calculation-protocol-with-materials-studio.

[ref88] Szell P. M. J.; Nilsson Lill S. O.; Blade H.; Brown S. P.; Hughes L. P. A toolbox for improving the workflow of NMR crystallography. Solid State Nucl. Magn. Reson. 2021, 116, 101761 10.1016/j.ssnmr.2021.101761. 34736104

[ref89] Monkhorst H. J.; Pack J. D. Special points for Brillouin-zone integrations. Phys. Rev. B 1976, 13 (12), 5188–5192. 10.1103/PhysRevB.13.5188.

[ref90] Tkatchenko A.; Scheffler M. Accurate Molecular Van Der Waals Interactions from Ground-State Electron Density and Free-Atom Reference Data. Phys. Rev. Lett. 2009, 102 (7), 073005 10.1103/PhysRevLett.102.073005. 19257665

[ref91] Harris R. K.; Hodgkinson P.; Pickard C. J.; Yates J. R.; Zorin V. Chemical shift computations on a crystallographic basis: some reflections and comments. Magn. Reson. Chem. 2007, 45 (S1), S174–S186. 10.1002/mrc.2132. 18157842

[ref92] Hartman J. D.; Monaco S.; Schatschneider B.; Beran G. J. O. Fragment-based ^13^C nuclear magnetic resonance chemical shift predictions in molecular crystals: An alternative to planewave methods. J. Chem. Phys. 2015, 143 (10), 102809 10.1063/1.4922649. 26374002

[ref93] Reddy G. N. M.; Cook D. S.; Iuga D.; Walton R. I.; Marsh A.; Brown S. P. An NMR crystallography study of the hemihydrate of 2′, 3′-O-isopropylidineguanosine. Solid State Nucl. Magn. Reson. 2015, 65, 41–48. 10.1016/j.ssnmr.2015.01.001. 25686689

[ref94] Cole J. C.; Korb O.; McCabe P.; Read M. G.; Taylor R. Knowledge-Based Conformer Generation Using the Cambridge Structural Database. J. Chem. Inf. Model 2018, 58 (3), 615–629. 10.1021/acs.jcim.7b00697. 29425456

[ref95] Gervais C.; Profeta M.; Lafond V.; Bonhomme C.; Azaïs T.; Mutin H.; Pickard C. J.; Mauri F.; Babonneau F. Combined ab initio computational and experimental multinuclear solid-state magnetic resonance study of phenylphosphonic acid. Magn. Reson. Chem. 2004, 42 (5), 445–452. 10.1002/mrc.1360. 15095380

[ref96] Yates J. R.; Pham T. N.; Pickard C. J.; Mauri F.; Amado A. M.; Gil A. M.; Brown S. P. An Investigation of Weak CH···O Hydrogen Bonds in Maltose Anomers by a Combination of Calculation and Experimental Solid-State NMR Spectroscopy. J. Am. Chem. Soc. 2005, 127 (29), 10216–10220. 10.1021/ja051019a. 16028932

[ref97] Mooney C. Z. Monte Carlo Simulation; Sage Publications: Thousand Oaks, California, 1997.

[ref98] Corlett E. K.; Blade H.; Hughes L. P.; Sidebottom P. J.; Walker D.; Walton R. I.; Brown S. P. An XRD and NMR crystallographic investigation of the structure of 2,6-lutidinium hydrogen fumarate. CrystEngComm 2019, 21 (22), 3502–3516. 10.1039/C9CE00633H.

[ref99] Webber A. L.; Emsley L.; Claramunt R. M.; Brown S. P. NMR Crystallography of Campho[2,3-c]pyrazole (Z′ = 6): Combining High-Resolution ^1^H-^13^C Solid-State MAS NMR Spectroscopy and GIPAW Chemical-Shift Calculations. J. Phys. Chem. A 2010, 114 (38), 10435–10442. 10.1021/jp104901j. 20815383

[ref100] Hoffmann-Jorgensen J.; Pisier G. The Law of Large Numbers and the Central Limit Theorem in Banach Spaces. Ann. Probab. 1976, 4 (4), 587–599. 10.1214/aop/1176996029.

[ref101] Stine R. An Introduction to Bootstrap Methods: Examples and Ideas. Sociol. Methods Res. 1989, 18 (2–3), 243–291. 10.1177/0049124189018002003.

[ref102] Pearson’s Correlation Coefficient. In Encyclopedia of Public Health; Kirch W., Ed.; Springer Netherlands: Dordrecht, 2008; pp 1090–1091.

[ref103] Cordova M.; Engel E. A.; Stefaniuk A.; Paruzzo F.; Hofstetter A.; Ceriotti M.; Emsley L. A Machine Learning Model of Chemical Shifts for Chemically and Structurally Diverse Molecular Solids. J. Phys. Chem. C 2022, 126 (39), 16710–16720. 10.1021/acs.jpcc.2c03854. PMC9549463 36237276

[ref104] Gerrard W.; Bratholm L. A.; Packer M. J.; Mulholland A. J.; Glowacki D. R.; Butts C. P. IMPRESSION – prediction of NMR parameters for 3-dimensional chemical structures using machine learning with near quantum chemical accuracy. Chem. Sci. 2020, 11 (2), 508–515. 10.1039/C9SC03854J. 32190270 PMC7067266

[ref105] Jonas E.; Kuhn S.; Schlörer N. Prediction of chemical shift in NMR: A review. Magn. Reson. Chem. 2022, 60 (11), 1021–1031. 10.1002/mrc.5234. 34787335

