# Supplementary material for: Polymorph Identification for Flexible Molecules: Linear Regression Analysis of Experimental and Calculated Solution- and Solid-State NMR Data
Source: J Phys Chem A. 2024 Mar 1;128(10):1793–816. doi: 10.1021/acs.jpca.3c07732 (PMC10945485; doi:10.1021/acs.jpca.3c07732)
Supplement: Supplementary file 1 — jp3c07732_si_001.pdf [file jp3c07732_si_001.pdf]

Polymorph Identification for Flexible Molecules: Linear Regression Analysis of Experimental and Calculated Solution- and Solid-state NMR Data Supporting Information

Mohammed Rahman[a,b$], Hugh R. W Dannatt[c$], Charles D. Blundell[c$*], Leslie P. Hughes[d*], Helen Blade[d*], Jake Carson[e], Ben P. Tatman[a,b], Steven T. Johnston[c], Steven P. Brown[a*].

[a]Department of Physics, University of Warwick, Coventry, CV4 7AL, UK
[b]Department of Chemistry, University of Warwick, Coventry, CV4 7AL, UK
[c]C4X Discovery, Manchester, M1 3LD, UK
[d]Oral Product Development, Pharmaceutical Technology & Development, Operations, AstraZeneca, Macclesfield, SK10 2NA, UK
[e]Mathematics Institute at Warwick, University of Warwick, Coventry, CV4 7AL, UK

[$]M.R., H.D. and C.B. contributed equally to this paper.

*Corresponding authors
Email:
S.P.Brown@warwick.ac.uk
Helen.Blade@astrazeneca.com
Les.Hughes2@astrazeneca.com
Charles.Blundell@c4xdiscovery.com

[$]M.R., H.D. and C.B. contributed equally to this paper.

Supporting Information

1. Introduction

TS1. Crystal packing similarity between crystal structures of furosemide polymorphs.
FS1. Comparison of conformations from each polymorph of furosemide.
FS2. Comparison of conformations from furosemide polymorphs with each other and the solution conformational distribution at each of the 6 torsions.
FS3. The FURSEM17 Form I structure has been solved with one carboxylic acid hydrogen in the wrong orientation.

2. Methods

FS4. Detailed flowchart of steps for enacting the Δδ regression approach.
FS5. Change of chemical shift of H7 with pH for measuring the pK_a value of furosemide.
FS6. Chemical shift of H7 with furosemide concentration in pure DMSO-d_6 for measuring furosemide's tendency to self-associate in solution.
FS7. Example portions of solution-state NMR spectra used for chemical shift assignment and the measurement of distance restraints.
TS2. Acquisition parameters for solution-state NMR data.
FS8. Overlay of the base conformation used in dynamic 3D-structure determination before and after DFT geometry optimisation.

3. Results

3.1 Measurement of solution-state NMR chemical shifts ($\delta_{Solution\ expt}$) and dynamic 3D structure

TS3. Conformational parameter values for the solution dynamic 3D-structure of furosemide.
FS9. Temperature coefficients and $^1J_{HN}$ coupling constants for H6 and H9* in solution.
TS4. Comparison of furosemide torsion angles values between the solution and solid states.

FS10. Comparison of furosemide solution dynamic 3D structure and polymorph conformations in conformational ensemble representation.

FS11. Comparison of polymorph conformations with each other and the solution dynamic 3D structure in torsion-population representation.

3.2 Calculation of solution-state NMR chemical shifts ($\delta_{Solution\ calc}$) from the solution dynamic 3D structure

TS5. Calculated mean chemical shift value for ensembles of different sizes from the solution dynamic 3D structure and neutral crystal structures.

FS12. Distributions of chemical shift values calculated for each nucleus from the *N* = 1,000 ensemble of conformations randomly selected from the solution dynamic 3D structure.

FS13. Quartile-Quartile (Q-Q) plots comparing a normal distribution with the distribution of calculated chemical shifts for 1,000 randomly sampled conformations from the solution dynamic 3D structure ensemble.

3.3 Experimental measurement of solid-state NMR chemical shifts ($\delta_{Solid\ expt}$)

There are no Supporting Information Figures or Tables for this section.

3.4 Calculation of solid-state NMR chemical shifts ($\delta_{Solid\ calc}$)

FS14. Overlay of the asymmetric units of each furosemide form from structures obtained at 100 K before and after geometry optimisation.

TS6. Crystal packing similarity of furosemide crystal structures after DFT geometry optimisation.

TS7. Torsion angles for each rotatable bond in furosemide crystal structures before and after DFT geometry optimisation (CASTEP).

3.5 Linear regression of $\Delta\delta_{Calculated}$ vs $\Delta\delta_{Experimental}$ and t-test to identify the correct form

TS8. The lower bound of the one-sided 95% confidence intervals for the correlation of $\Delta\delta_{Calculated}$ vs $\Delta\delta_{Experimental}$ (see Table 4).

TS9. Linear regression analysis parameters and p-values for combinations of calculated (Forms I, II, III) and experimentally measured (Form I, Molecule A and Molecule B) changes in furosemide chemical shift *when data from all the ^{1}H atoms in exchange (H6, H9*, and H11) and the ^{13}C atom adjacent to the chlorine (C8) are included.*

TS10. The lower bound of the one-sided 95% confidence intervals for the correlation of $\Delta\delta_{Experimental}$ vs $\Delta\delta_{Calculated}$ *when data from all the ^{1}H atoms in exchange (H6, H9*, and H11) and the ^{13}C atom adjacent to the chlorine (C8) are included* (see Table S9).

TS11. Linear regression analysis parameters and p-values for calculated and experimentally measured changes in furosemide chemical shift for Form I structures (both molecules A and B treated together).

TS12. Linear regression analysis parameters and *p*-values for calculated and experimentally measured changes in furosemide chemical shift for Form I structures (both molecules A and B treated together) *when data from all the ^{1}H atoms in exchange (H6, H9*, and H11) and the ^{13}C atom adjacent to the chlorine (C8) are included*.

3.6 Analysis 1: Comparison with the RMSE method

TS13. Linear regression analysis parameters and RMSE values for combinations of calculated (Forms I, II, III) and experimentally measured (Form I, Molecule A and Molecule B) solid-state NMR chemical shifts.

3.7 Analysis 2: Effects of allowing unit cell parameters to vary during geometry optimisation

TS14A. Relative energies and densities of crystal structures of furosemide after DFT geometry optimisation *with unit cell parameters being fixed or allowed to vary during optimisation.*

TS14B. Changes to unit cell parameters for crystal structures of furosemide after DFT geometry optimisation *with unit cell parameters being fixed or allowed to vary (with DFT-D) during optimisation.*

TS15. Crystal packing similarity between crystal structures of furosemide after DFT geometry optimisation *with unit cell parameters being fixed or allowed to vary during optimisation.*

TS16. Experimentally measured and calculated GIPAW NMR chemical shifts for furosemide in solution and in solid-state forms *after allowing the unit cell to vary during DFT-D geometry optimisation.*

TS17. Comparison of the experimentally measured ($\Delta\delta_{Experimental}$) and GIPAW calculated ($\Delta\delta_{Calculated}$) differences in NMR chemical shifts for furosemide between the solution state and solid-state forms *after allowing the unit cell to vary during DFT-D geometry optimisation.*

TS18. Linear regression analysis parameters and *p*-values for chemical shift differences between the solution state and the solid state for combinations of calculated (Forms I, II, III) and experimentally measured (Form I, Molecule A and Molecule B) differences in furosemide NMR chemical shift *after allowing the unit cell to vary during DFT-D geometry optimisation.*

TS19. The lower bound of the one-sided 95% confidence intervals for the correlation of $\Delta\delta_{Experimental}$ vs $\Delta\delta_{Calculated}$ after allowing the unit cell to vary during DFT-D geometry optimisation.

TS20. Linear regression analysis parameters and RMSE values for combinations of calculated (Forms I, II, III) and experimentally measured (Form I, Molecule A and Molecule B) solid-state NMR chemical shifts *after allowing the unit cell to vary during DFT-D geometry optimisation.*

TS21. Comparison of approaches for identifying the correct form from solid state NMR chemical shift data *after allowing the unit cell to vary during DFT-D geometry optimisation.*

3.8 Analysis 3: Choice of solvent and charge-state for solution data

TS22. Solution-state NMR chemical shifts for furosemide ($\delta_{Solution\ expt}$) under different solvent conditions.

FS15. Change in absolute solution-state NMR chemical shift with change in solvent conditions.

TS23. Comparison of the experimentally measured ($\Delta\delta_{Experimental}$) and DFT GIPAW calculated ($\Delta\delta_{Calculated}$) changes in NMR chemical shifts, *using solution chemical shifts measured in an aqueous environment in the neutral state.*

TS24. Linear regression analysis parameters and *p*-values *using solution chemical shifts measured in an aqueous environment in the neutral state.*

TS25. Lower bound of the one-sided 95% confidence intervals for the correlation of $\Delta\delta_{Calculated}$ vs $\Delta\delta_{Experimental}$ *using solution chemical shifts measured in an aqueous environment in the charged state.*

TS26. $\delta_{Solution\ calc}$ values for furosemide in neutral and charged states.

TS27. Comparison of the experimentally measured ($\Delta\delta_{Experimental}$) and DFT GIPAW calculated ($\Delta\delta_{Calcualted}$) changes in NMR chemical shifts, *using solution chemical shifts measured in an aqueous environment in the charged state.*

TS28. Linear regression analysis parameters and *p*-values *using solution chemical shifts measured in an aqueous environment in the charged state.*

TS29. Lower bound of the one-sided 95% confidence intervals for the correlation of $\Delta\delta_{Calculated}$ vs $\Delta\delta_{Experimental}$ *using solution chemical shifts measured in an aqueous environment in the neutral state.*

3.9 Analysis 4: Approximation of solution dynamic 3D structure using a substitute ensemble of furosemide conformations from the CSD

TS30. Torsion angle values from all single-crystal diffraction structures in the CSD[a] containing neutral furosemide.

FS16. Comparison of furosemide solution dynamic 3D structure (grey carbons, in conformational ensemble representation) and the substitute ensemble (yellow carbons, 'CSD-SX') for estimating $\delta_{Solution\ calc}$ created from neutral furosemide conformations in single crystals structures in the CSD.

TS31. $\delta_{Solution\ calc}$ values for furosemide from the CSD-SX substitute ensemble (108 conformations) compared to the solution dynamic 3D structure.

FS17. Change in calculated solution chemical shift between solution dynamic 3D structure and the substitute ensemble created from neutral conformations of furosemide in single-crystal diffraction structures.

TS32. Linear regression analysis parameters and *p*-values *using a substitute ensemble from CSD single-crystal diffraction structures to calculate* $\delta_{Solution\ calc}$.

TS33. The lower bound of the one-sided 95% confidence intervals for the correlation of $\Delta\delta_{Experimental}$ vs $\Delta\delta_{Calculated}$ *using a substitute ensemble from CSD single-crystal diffraction structures to calculate* $\delta_{Solution\ calc}$.

Supporting Information References

User guide for accompanying Graphical User Interface for data processing

1. Introduction

Table S1. Crystal packing similarity between crystal structures of furosemide polymorphs, as calculated using the crystal packing similarity tool within Mercury using default settings.

A) Number of molecules in common out of 15.

			Form I						Form II			Form III
			FURSEM13 100 K	FURSEM18 120 K	FURSEM03 173 K	FURSEM01 295 K	FURSEM17 293 K	FURSEM02 295 K	FURSEM14 100 K	FURSEM15 (75%) 293 K	FURSEM15 (25%) 293 K	FURSEM16 100 K
Form I	FURSEM13	100 K	-	15	15	15	15	1	1	1	1	1
	FURSEM18	120 K	15	-	15	15	15	2	1	1	1	1
	FURSEM03	173 K	15	15	-	15	15	2	1	1	1	1
	FURSEM01	295 K	15	15	15	-	15	2	1	1	1	1
	FURSEM17	293 K	15	15	15	15	-	2	1	1	1	2
	FURSEM02	295 K	1	2	2	2	2	-	1	1	1	1
Form II	FURSEM14	100 K	1	1	1	1	1	1	-	1	15	1
	FURSEM15 (75%)	293 K	1	1	1	1	1	1	1	-	2	1
	FURSEM15 (25%)	293 K	1	1	1	1	1	1	15	2	-	1
Form III	FURSEM16	100 K	1	1	1	1	2	1	1	1	1	-

B) RMSD for the 15 molecules (Å).

			Form I						Form II			Form III
			FURSEM13 100 K	FURSEM18 120 K	FURSEM03 173 K	FURSEM01 295 K	FURSEM17 293 K	FURSEM02 295 K	FURSEM14 100 K	FURSEM15 (75%) 293 K	FURSEM15 (25%) 293 K	FURSEM16 100 K
Form I	FURSEM13	100 K	-	0.022	0.086	0.140	0.139	0.699	1.520	1.248	1.506	0.820
	FURSEM18	120 K	0.022	-	0.063	0.119	0.124	0.703	1.522	1.248	1.507	0.821
	FURSEM03	173 K	0.086	0.063	-	0.076	0.079	0.700	1.522	1.247	1.508	0.822
	FURSEM01	295 K	0.140	0.119	0.076	-	0.015	0.698	1.524	1.250	1.510	0.820
	FURSEM17	293 K	0.139	0.124	0.079	0.015	-	0.697	1.523	1.250	1.508	0.845
	FURSEM02	295 K	0.699	0.703	0.700	0.698	0.697	-	1.704	1.568	1.687	1.251
Form II	FURSEM14	100 K	1.520	1.522	1.522	1.524	1.523	1.704	-	0.833	0.109	1.204
	FURSEM15 (75%)	293 K	1.248	1.248	1.247	1.250	1.250	1.568	0.833	-	0.830	0.824
	FURSEM15 (25%)	293 K	1.506	1.507	1.508	1.510	1.508	1.687	0.109	0.830	-	1.188
Form III	FURSEM16	100 K	0.820	0.821	0.822	0.82	0.845	1.251	1.204	0.824	1.188	-

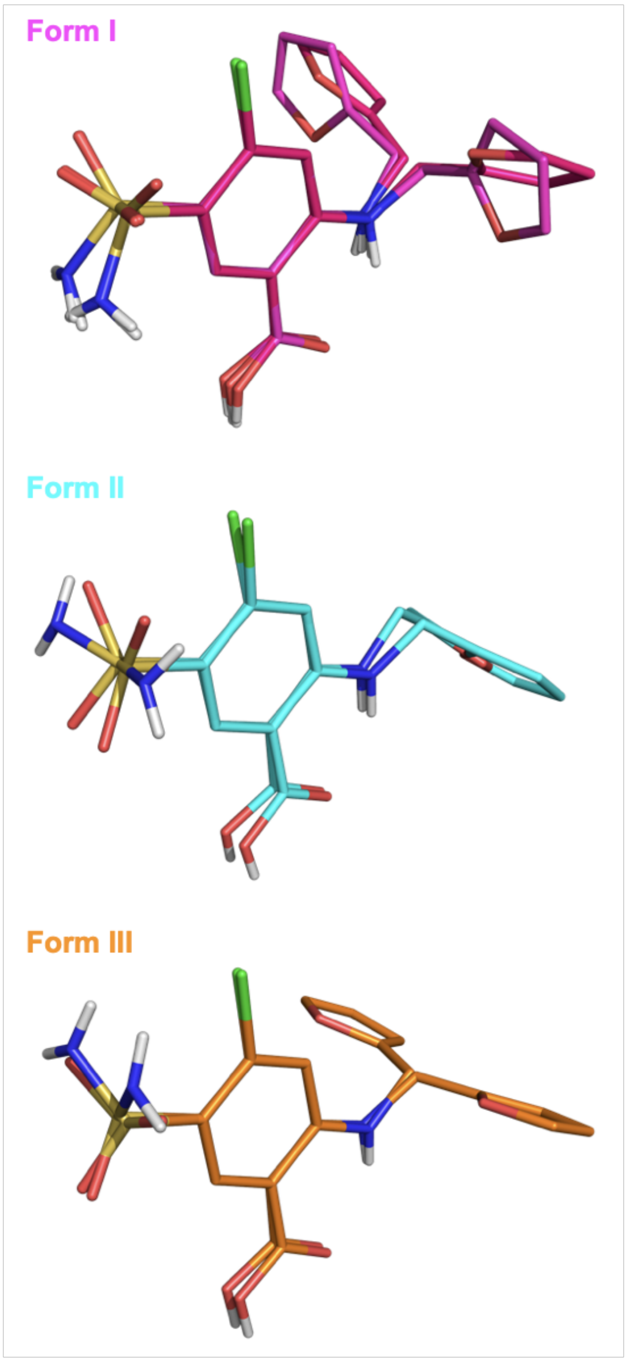

Figure S1. Comparison of conformations from each polymorph of furosemide. Conformations have been extracted from the crystal structures at 100 K and overlaid in the same reference frame to highlight differences (CSD entry IDs FURSEM13, FURSEM14, and FURSEM16, respectively). Most of the variation in conformation between forms is found at torsions 1, 2 and 4 (see also Figure S2; torsion definitions are given in Figure 1). Form I displays two distinct conformations and their mirror images, having *Z*' = 2 and *Z* = 4; these are defined as Molecules A and B by their torsion 1 values of +68° and –57°, respectively (see Table S7). Forms II and III both have one conformation each and its mirror image, having *Z*' = 1 and *Z* = 2 or 4 (respectively). Oxygen atoms are coloured red, nitrogen blue, chlorine green, hydrogen white and carbons in magenta (I), cyan (II) or orange (III). Non-polar hydrogens have been omitted for clarity.

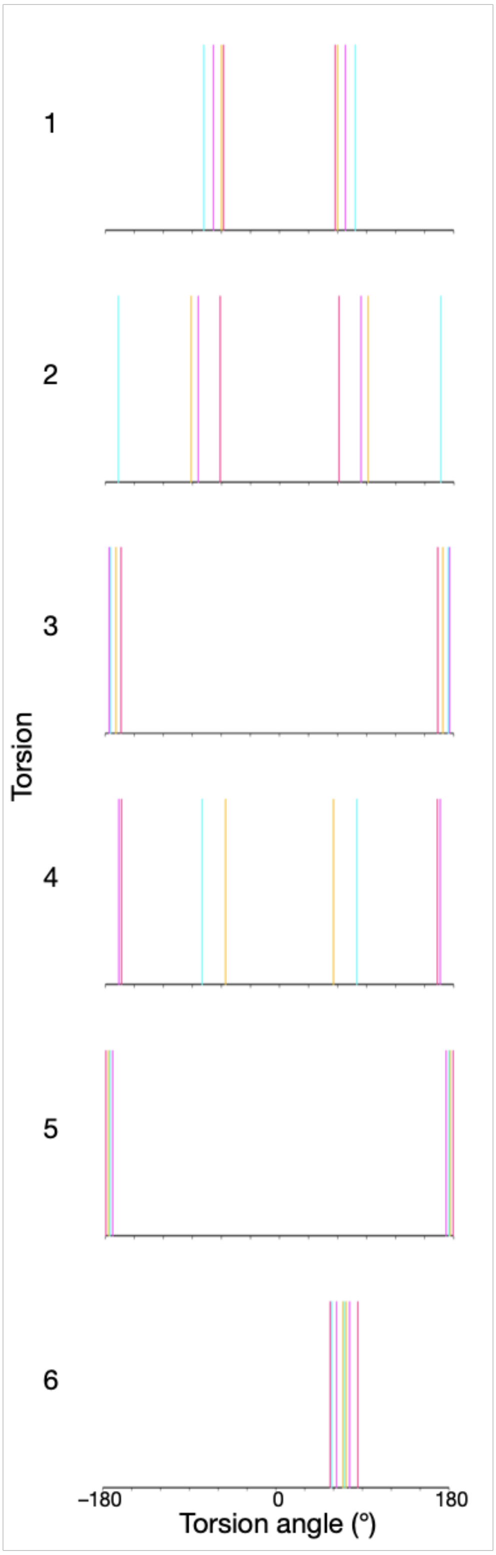

Figure S2. Comparison of conformations from furosemide polymorphs with each other at each of the 6 torsions (Form I – magenta; Form II – cyan; Form III – orange, see Table S7 for values). Most of the variation in conformation between forms is found at torsions 1, 2 and 4 (see also Figure S1; torsion definitions are given in Figure 1). Values are taken from crystal structures at 100 K, including both the *Z*' conformation and its *Z* mirror image conformation from the unit cell (CSD entry IDs FURSEM13, FURSEM14, and FURSEM16, for Forms I, II and III, respectively) – see Table S4.

Form I - 100 K
(FURSEM13)

Form I - 295 K
(FURSEM01)

Form I - 293 K
(FURSEM17)

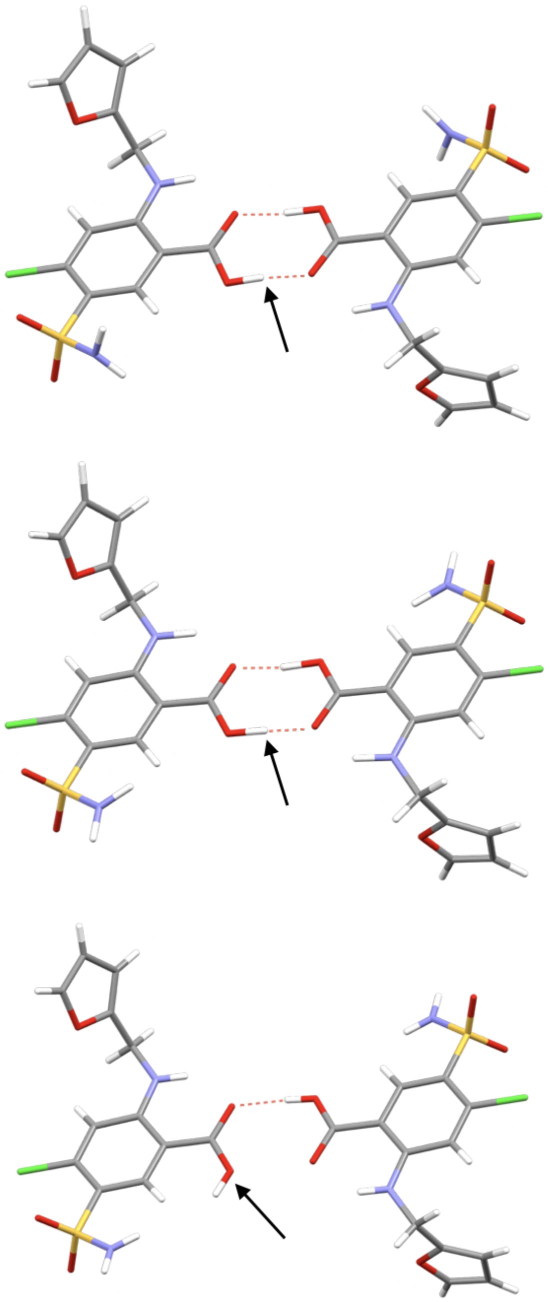

Figure S3. The FURSEM17 Form I structure has been solved with one carboxylic acid hydrogen in the wrong orientation. Across all forms of furosemide, and all other Form I structures from 100 K (FURSEM13) to 293 K (FURSEM01), reciprocal hydrogen-bonded dimers between carboxylic acid groups are formed. This feature is only half present in the FURSEM17 structure and is therefore probably erroneous; unfortunately, DFT geometry optimisation does not correct its position. In contrast, note also that although the sulphonamide NH_2 geometries in the room temperature structures (FURSEM01, FURSEM17) are not clearly resolved like they are at 100 K (FURSEM13), and are consequently modelled in slightly erroneous positions, these flaws are corrected during DFT geometry optimisation. Atoms are coloured by element (carbon grey, oxygen red, nitrogen blue, chlorine green, hydrogen white) and all hydrogens are shown.

2. Methods

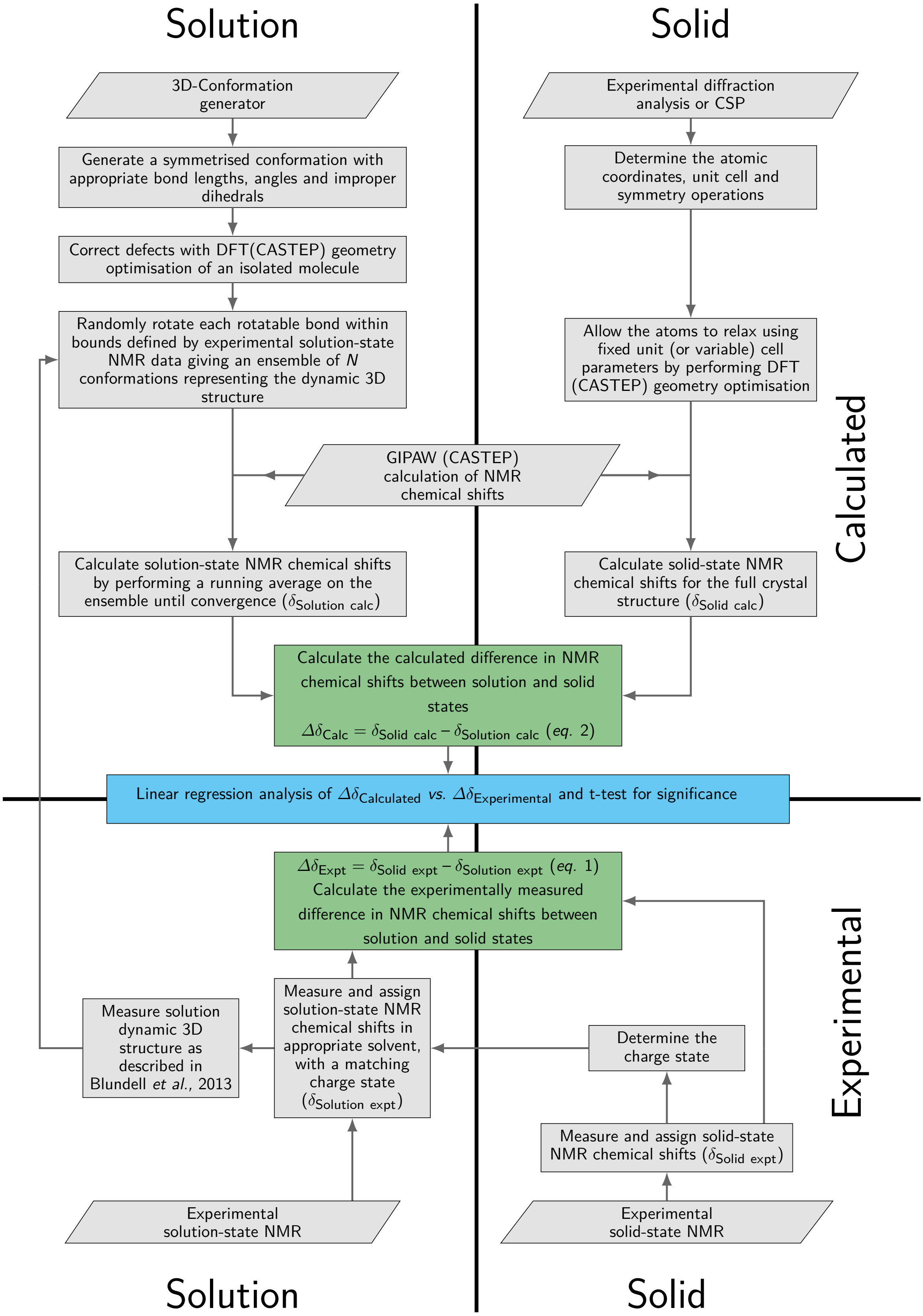

Figure S4. Detailed flowchart of steps for enacting the Δδ regression approach described in this work.

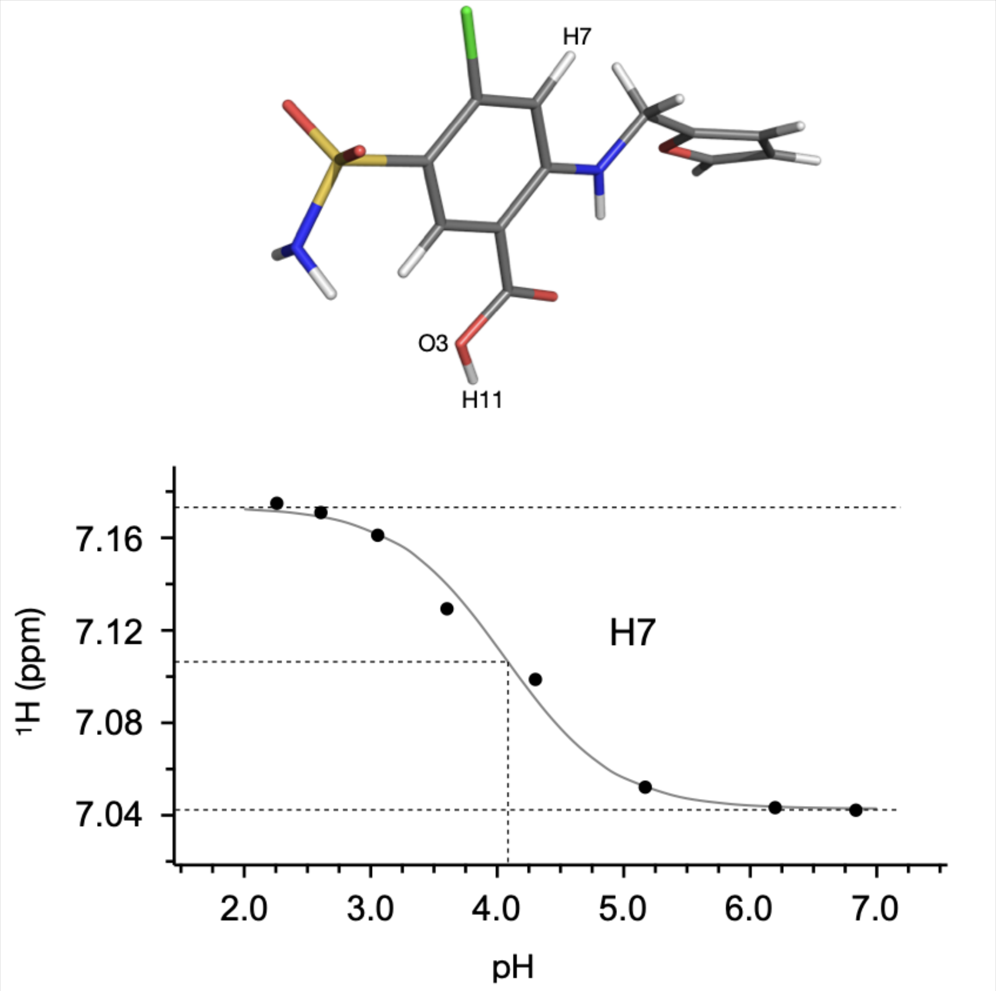

Figure S5. The change of ^{1}H NMR chemical shift of H7 with pH as a reporter proton for measuring the pK_a value of furosemide as H11 is protonated/deprotonated at the carboxylic acid/carboxylate oxygen O3. The pK_a value was determined to be 4.11 ± 0.05 in 80% H_2O, 20% DMSO-d_6 at 25 ºC using a sample containing 1 mM furosemide.

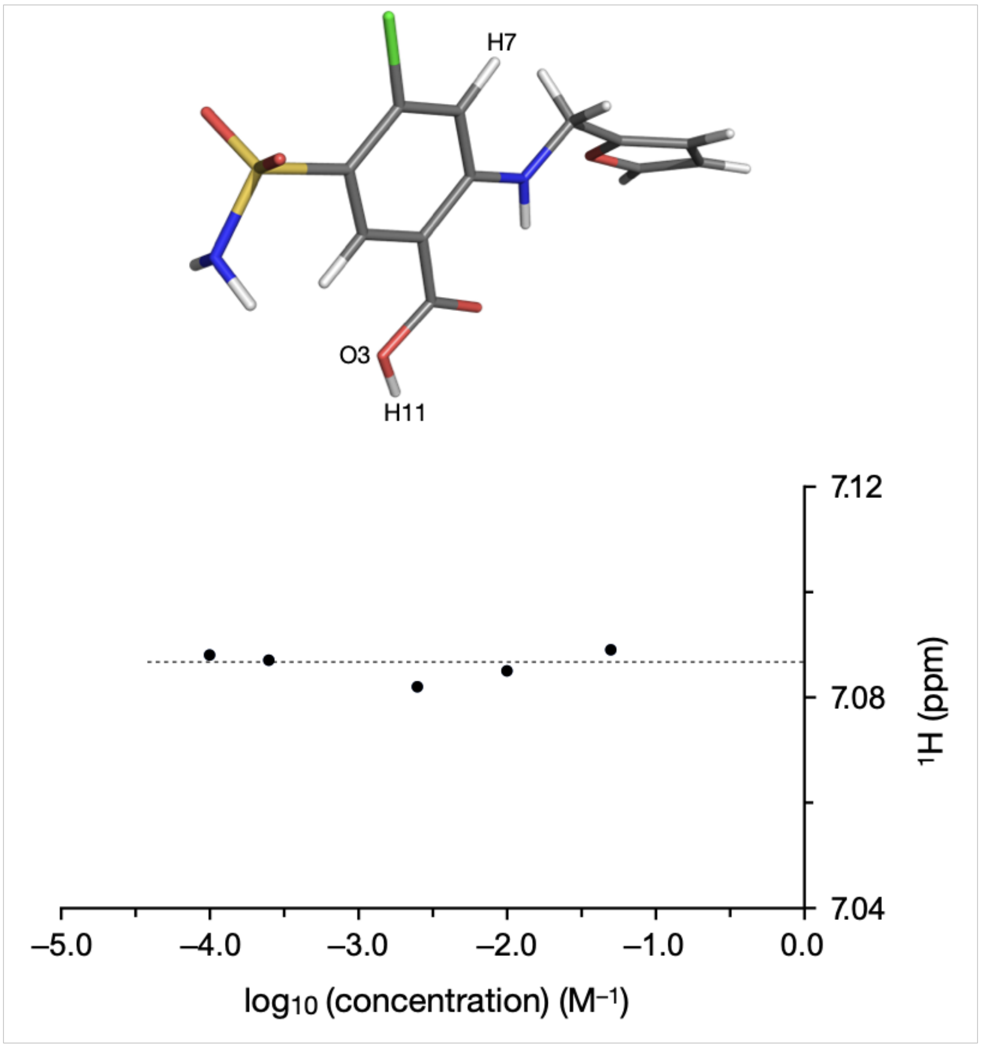

Figure S6. The dependence of the ^{1}H NMR chemical shift of H7 with furosemide concentration in pure DMSO-d_6 as a reporter proton for measuring furosemide's tendency to self-associate in solution. The flat line indicates that, within experimental error, there is no discernible self-association over the concentration range explored in this study. The study was performed at 25 ºC.

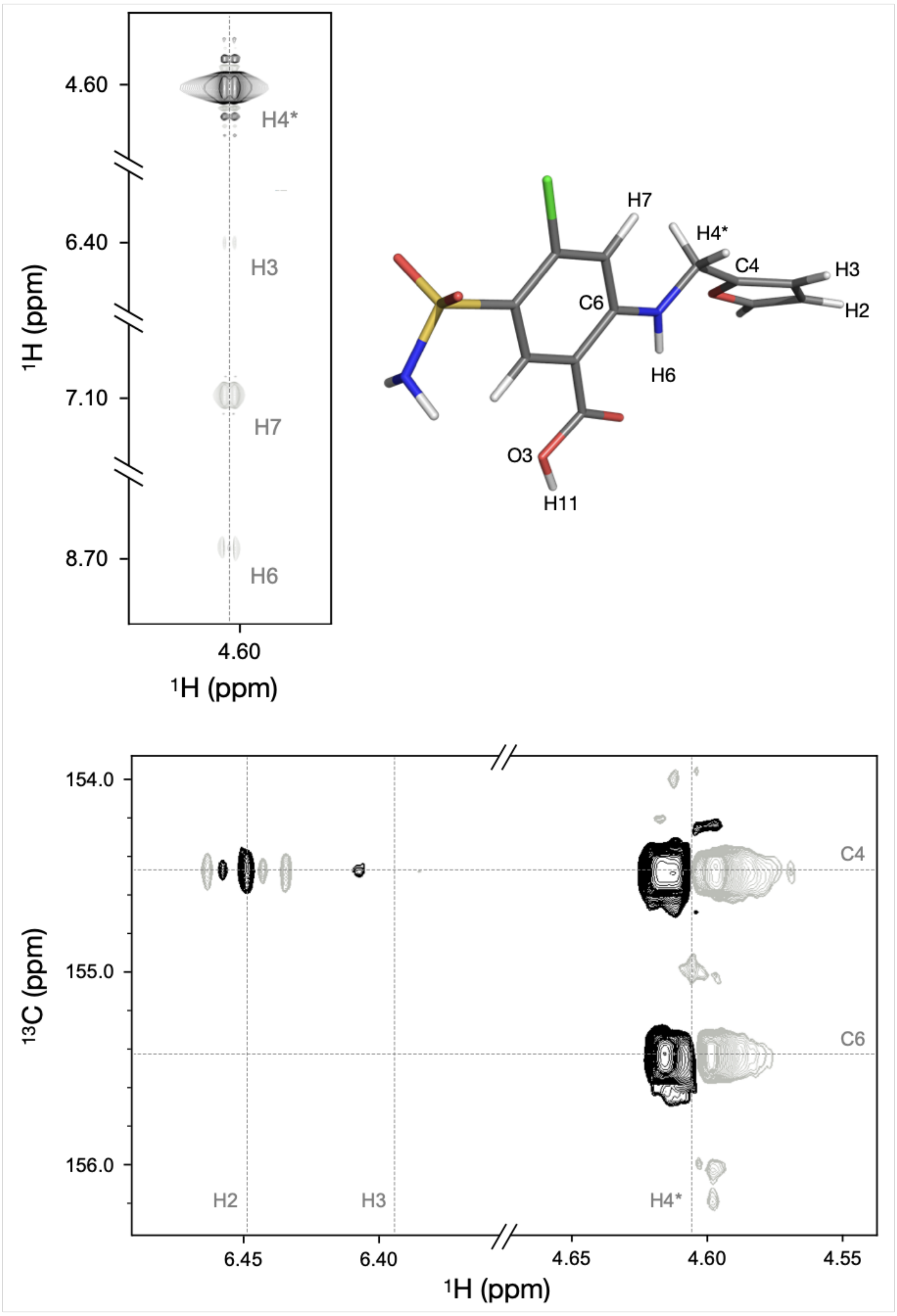

Figure S7. Example portions of solution-state NMR spectra used for chemical shift assignment (bottom, [^{1}H, ^{13}C]-IMPACT HMBC) and the measurement of distance restraints (top, [^{1}H, ^{1}H]-EASY-ROESY, mixing time 400 ms). Acquisition parameters are given in Table S2.

Table S2. Acquisition parameters for solution-state NMR data.

	^{1}H-1D	[^{1}H-^{13}C]-HSQC	[^{1}H-^{13}C]--HMBC	[^{1}H,^{1}H]-ROESY
**Spectral window (F_1) (ppm)**	-	100-150	100-190	4-9.5
**No. of data points (F_1)**	-	500	900	250
**Spectral resolution (F_1) (ppm)**	-	0.2	0.2	0.044
**F_1 sampling, %**	-	25	25	100
**Mixing time (s)**	-	-	-	0.4
**Total acquisition time**	1 min	24 min	1 h 15 min	3x 4 h 36 min
**References**	Liu *et al.*[1]<br>Adams *et al.*[2]	Palmer *et al.*[3]<br>Kay *et al.*[4]<br>Schleucher *et al.*[5]<br>Kupce *et al.*[6]	Claridge *et al.*,[7]<br>J. Furrer,[8]	Schleucher *et al.*[9]<br>Thiele *et al.*[10]<br>Mo *et al.*[11]<br>Liu *et al.*[1]<br>Adams *et al.*[2]

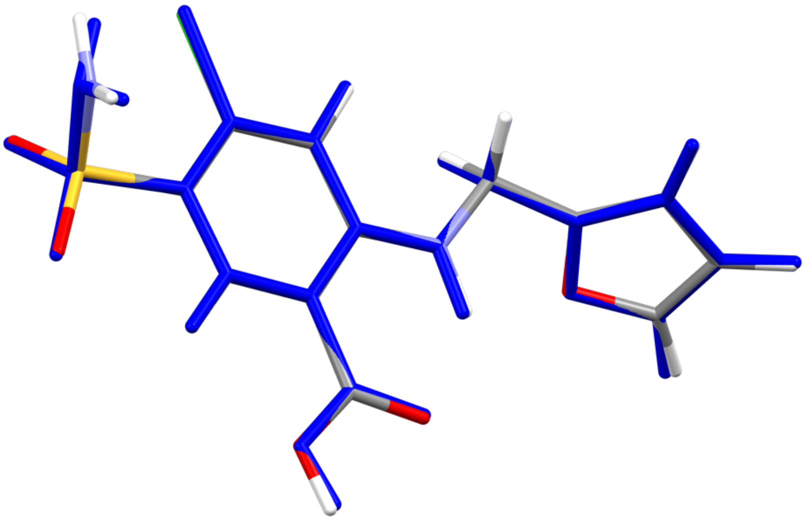

Figure S8. Overlay of the base conformation of furosemide used in dynamic 3D-structure determination before (coloured by element) and after (blue) DFT GIPAW geometry optimisation (CASTEP). The largest changes are observed at the geometry of the sulphonamide nitrogen, which adopts a more *sp*^{3}-like character after optimisation. The RMSD for all heavy atoms is 0.053 Å and all atoms is 0.054 Å, agreeing with the visual result that the conformation has been barely changed by the DFT geometry optimisation. All hydrogens are shown.

3. Results

3.1 Measurement of solution-state NMR chemical shifts ($\delta_{Solution\ expt}$) and dynamic 3D structure

Table S3. Experimentally measured conformational parameter values for the solution dynamic 3D-structure of furosemide.[a]

Torsion	Torsion definition[b]	Mode	Population (%)	Mean angle (°)	Libration (°)
1	O1−C4−C5−N1	1	45 ± 2[c]	−62 ± 4	15 ± 5
		2	45 ± 2	62 ± 4	15 ± 5
		3	10 ± 2	180± 2[d]	15 ± 5
2	C4−C5−N1−C6	1	47 ± 3	−91 ± 2	15 ± 5
		2	47 ± 2	91 ± 2	15 ± 5
		3	6 ± 3	180 ± 2[d]	15 ± 5
3	C5−N1−C6−C11	1	100 ± 2	180 ± 2[d]	5 ± 5
4	C8−C9−S1−N2	1	44 ± 3	−60 ± 2	10 ± 5
		2	44 ± 3	60 ± 2	10 ± 5
		3	12 ± 2	180 ± 2[d]	10 ± 5
5	C6−C11−C12−O3	1	100 ± 2	180 ± 2[d]	0 ± 5
6	C9−S1−N2−H9	1	100 ± 2	60 ± 2	10 ± 5

[a]Solution dynamic 3D structure was determined in DMSO-d_6 at 10 mM and 25 ºC according to the method of Blundell *et al.*[12]
[b]Refer to Figure 1 for atom definitions.
[c]Measured value and its standard deviation.
[d]Molecular symmetry would necessitate that any deviation from 180° is matched by a corresponding mode in the opposite direction; there was no evidence for such a bimodal behaviour.

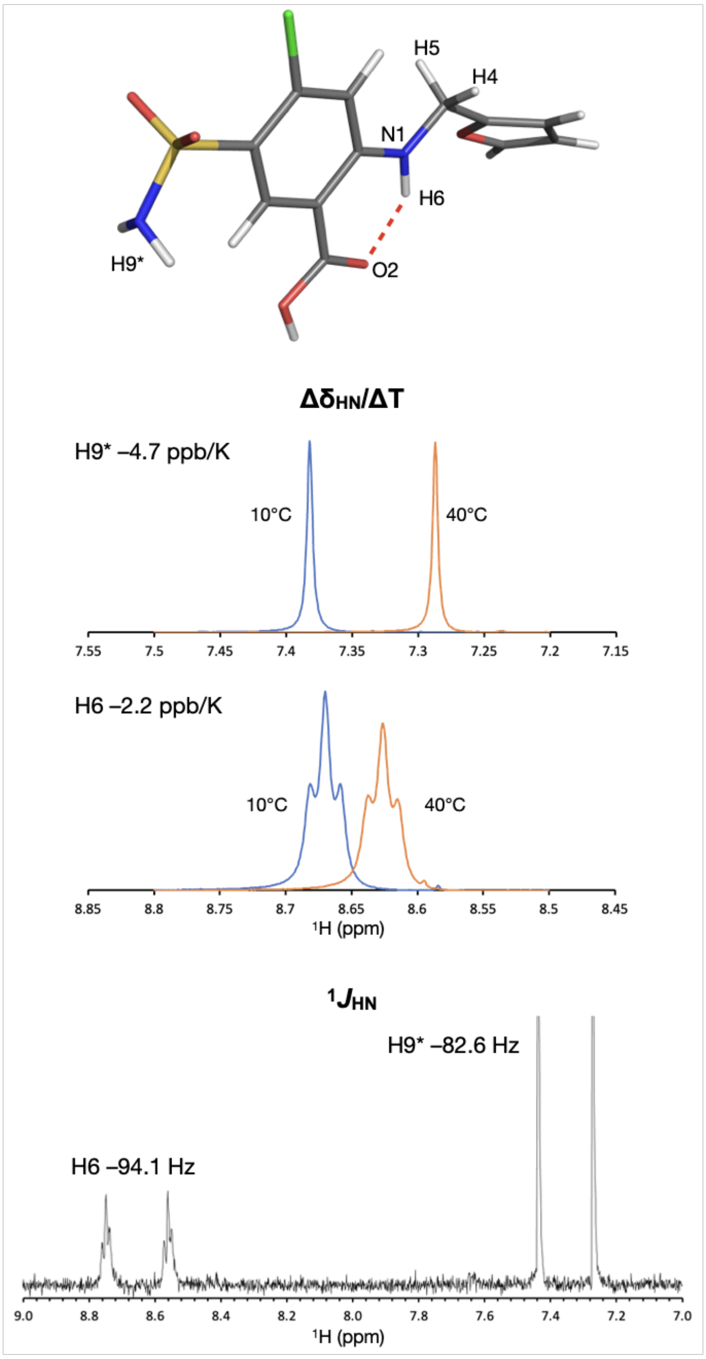

Figure S9. (Top) Location of intramolecular hydrogen bond between H6 and O2 shown on an arbitrary conformation present in solution. The sulphonamide hydrogens (H9, H10) are in rapid exchange in solution and manifest in spectra as a single resonance, labelled H9*; the aniline hydrogen H6 manifests as a triplet due to scalar coupling with H4 and H5. Atoms are coloured by element (carbon grey, oxygen red, nitrogen blue, chlorine green, hydrogen white) and non-polar hydrogens have been omitted for clarity. (Middle) Overlay of portions of ^{1}H NMR spectra for H6 and H9* showing the movement with temperature between 10° and 40 °C and associated temperature coefficients ($\Delta\delta_{HN}/\Delta T$). Data was recorded in the neutral state in pure DMSO-d_6 at 10 mM. (Bottom) Measurement of $^{1}J_{HN}$ coupling constants to assess extent of nitrogen sp^2/sp^3 hybridisation character. A ^{15}N-filtered 1D ^{1}H spectrum recorded without heteronuclear decoupling during acquisition on a sample of 10 mM furosemide in DMSO-d_6 is shown.

Table S4. Comparison of furosemide torsion angle values between the solution and solid states.

Torsion[b]	Solution dynamic 3D structure[a]				Form I 100 K (FURSEM13)		Form II 100 K (FURSEM14) (°)	Form III 100 K (FURSEM16) (°)
	Mode	Population (%)	Mean angle (°)	Libration (°)	A (°)	B (°)		
1	1	45 ± 2[c]	–62 ± 4	15 ± 5	68.2	–57.6	–78.2	–60.0
	2	45 ± 2	62 ± 4	15 ± 5	–68.2	57.6	78.2	60.0
	3	10 ± 2	180 ± 2[d]	15 ± 5				
2	1	47 ± 3	–91 ± 2	15 ± 5	–84.0	–61.4		91.3
	2	47 ± 2	91 ± 2	15 ± 5	84.0	61.4		–91.3
	3	6 ± 3	180 ± 2[d]	15 ± 5			–166.4<br>166.4	
3	1	100 ± 2	180 ± 2[d]	5 ± 5	175.8<br>–175.8	163.6<br>–163.6	174.3<br>–174.3	–168.8<br>168.8
4	1	44 ± 3	–60 ± 2	10 ± 5			–79.9	55.7
	2	44 ± 3	60 ± 2	10 ± 5			79.9	–55.7
	3	12 ± 2	180 ± 2[d]	10 ± 5	166.0<br>–166.0	163.2<br>–163.2		
5	1	100 ± 2	180 ± 2[d]	0 ± 5	172.3<br>–172.3	179.4<br>–179.4	175.2<br>–175.2	176.5<br>–176.5
6	1	100 ± 2	60 ± 2	10 ± 5	76.6<br>62.9	85.3<br>56.5	58.2<br>71.2[e]	69.5<br>72.9

[a]Solution dynamic 3D structure was determined in DMSO-d_6 at 10 mM and 25 ºC according to the method of Blundell *et al.*[12] All values as per Table S3.
[b]Refer to Figure 1 for atom definitions.
[c]Measured value and its standard deviation.
[d]Molecular symmetry would necessitate that any deviation from 180° is matched by a corresponding mode in the opposite direction; there was no evidence for such a bimodal behaviour in solution, though it is clearly possible as torsion 3 in the solid forms displays.
[e]Since the torsion 6 definition is to a *pro*-R hydrogen (H9), the equivalent values to the mirror-image molecule in the unit cell are not opposite in sign as they are for torsions 1-5.

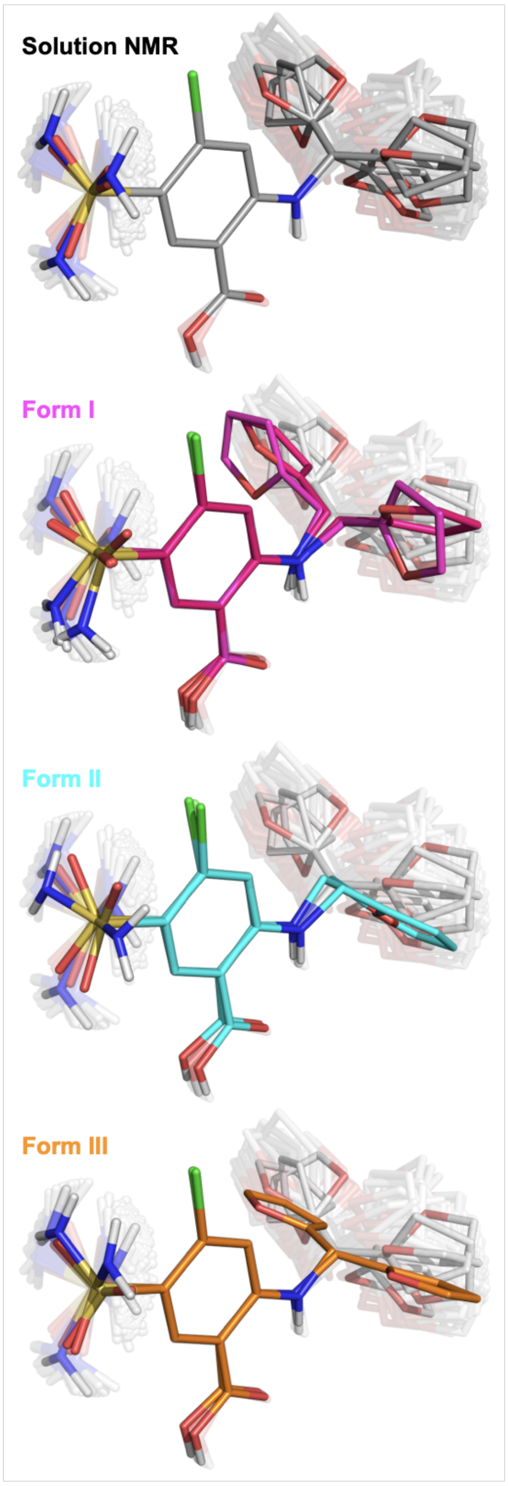

Figure S10. Comparison of furosemide solution dynamic 3D structure and polymorph conformations (coloured carbons) in a conformational ensemble representation. The solution dynamic 3D structure is shown with all its conformers (bright) overlaid on an ensemble representative for the range of libration in solution (faded). Crystal conformations have been extracted from the 100 K crystal structures (CSD entry IDs FURSEM13, FURSEM14, and FURSEM16, for Forms I, II and III, respectively). All conformations are overlaid on the central aromatic ring. Oxygen atoms are coloured red, nitrogen blue, chlorine green, hydrogen white and carbons in grey (solution), magenta (I), cyan (II) or orange (III). Non-polar hydrogens have been omitted for clarity.

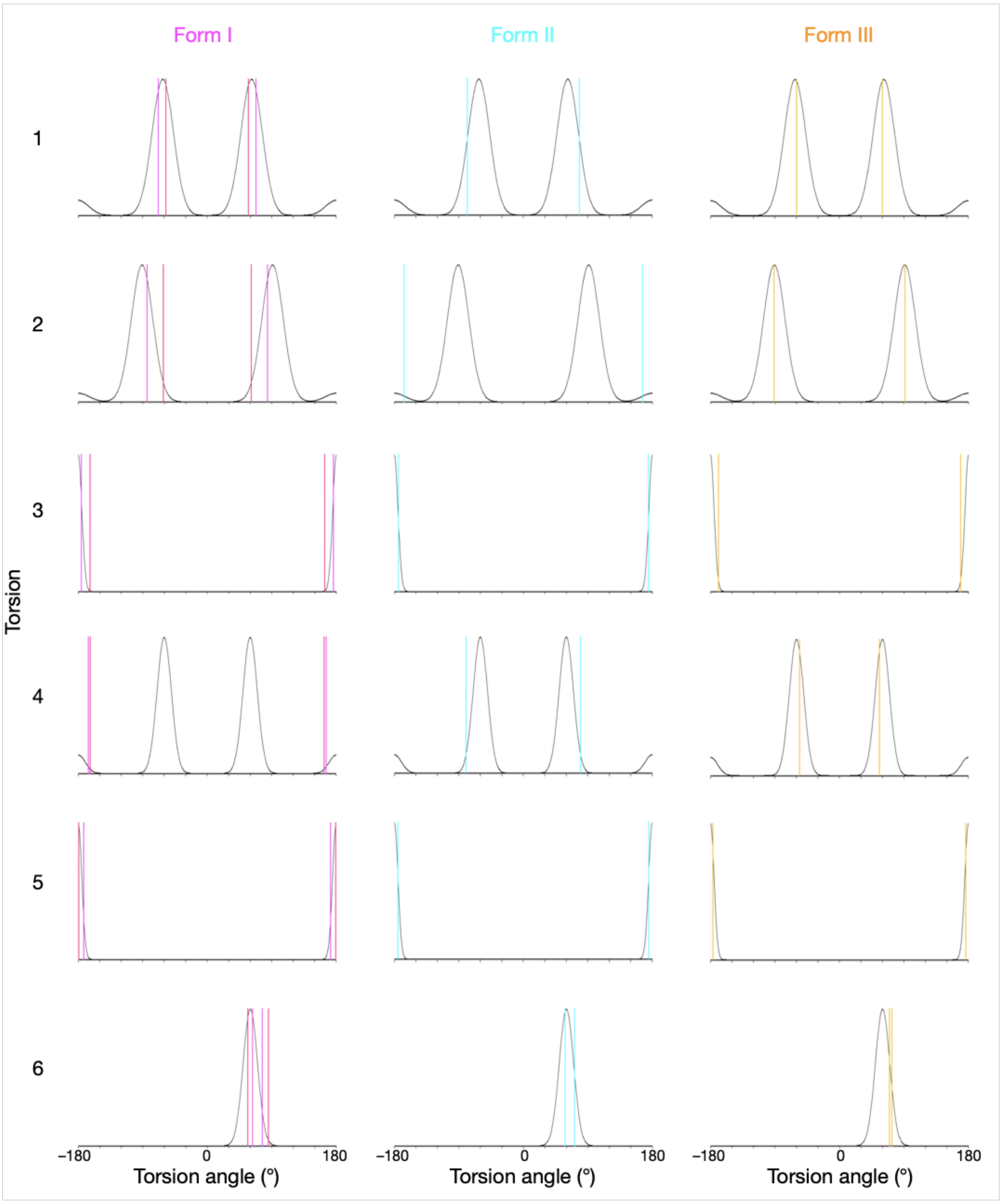

Figure S11. Comparison of conformations from furosemide polymorphs (colours) with each other and the solution dynamic 3D structure (continuous line graphs) at each of the 6 torsions in a torsion-population representation (Form I – magenta; Form II – cyan; Form III – orange; see Table S7 for values). Most of the variation in conformation between forms is found at torsions 1, 2 and 4 (see also Figure S1; torsion definitions are given in Figure 1). Values are taken from crystal structures at 100 K including both the *Z*' conformation and its *Z* mirror image conformation from the unit cell (CSD entry IDs FURSEM13, FURSEM14, and FURSEM16, for Forms I, II and III, respectively) – see Table S4.

3.2 Calculation of solution-state NMR chemical shifts ($\delta_{Solution\ calc}$) from the solution dynamic 3D structure

Table S5. Calculated mean NMR chemical shift value for ensembles of different sizes derived from random-sampling of the solution dynamic 3D structure, random resampling, and a substitute set of neutral single-crystal structures.

Atom[a]	$\delta_{Solution\ calc}$ (ppm)									
	N = 10[b]	*N* = 20	*N* = 50	*N* = 100	*N* = 150	*N* = 250	*N* = 500	*N* = 1,000	*M* = 1,000[c]	SX substitute set (*N* = 108)[d]
C1	142.7 ± 0.46[e]	**142.6 ± 0.33**[f]	142.6 ± 0.25	142.6 ± 0.17	142.6 ± 0.13	142.5 ± 0.11	142.5 ± 0.07[g]	142.5 ± 0.05	142.5 ± 0.05[c]	143.0 ± 0.19
C2	**108.5 ± 0.25**	108.5 ± 0.18	108.6 ± 0.13	108.5 ± 0.10	108.5 ± 0.07	108.5 ± 0.06	108.5 ± 0.04	108.5 ± 0.03	108.5 ± 0.03	108.5 ± 0.15
C3	105.7 ± 0.69	106.2 ± 0.97	106.4 ± 0.61	106.4 ± 0.37	106.4 ± 0.31	**106.2 ± 0.22**	106.2 ± 0.15	106.2 ± 0.11	106.3 ± 0.12	107.9 ± 0.47
C4	152.9 ± 0.87	152.6 ± 0.73	152.6 ± 0.39	152.6 ± 0.29	**152.7 ± 0.24**	152.8 ± 0.19	152.8 ± 0.13	152.8 ± 0.09	152.7 ± 0.08	151.8 ± 0.45
C5	36.5 ± 1.07	36.3 ± 0.61	**36.2 ± 0.34**	36.2 ± 0.24	36.2 ± 0.19	36.1 ± 0.15	36.1 ± 0.10	36.1 ± 0.07	36.1 ± 0.07	36.5 ± 0.35
C6	**149.8 ± 0.71**	149.7 ± 0.40	149.7 ± 0.24	149.7 ± 0.15	149.8 ± 0.12	149.8 ± 0.09	149.8 ± 0.07	149.8 ± 0.05	149.8 ± 0.05	149.8 ± 0.20
C7	111.4 ± 1.67	111.6 ± 1.18	111.4 ± 0.55	111.6 ± 0.44	111.7 ± 0.34	112.1 ± 0.34	**111.9 ± 0.23**	111.9 ± 0.16	111.9 ± 0.18	112.1 ± 0.41
C8	142.5 ± 0.70	142.8 ± 0.66	143.2 ± 0.35	**143.0 ± 0.27**	143.0 ± 0.22	142.9 ± 0.18	143.0 ± 0.12	143.0 ± 0.09	142.9 ± 0.09	144.3 ± 0.48
C9	131.5 ± 0.42	131.4 ± 0.31	**131.5 ± 0.26**	131.6 ± 0.17	131.6 ± 0.15	131.5 ± 0.11	131.5 ± 0.08	131.5 ± 0.06	131.6 ± 0.05	130.0 ± 0.51
C10	133.6 ± 0.46	133.4 ± 0.57	**133.1 ± 0.50**	133.1 ± 0.33	133.0 ± 0.27	133.1 ± 0.20	133.1 ± 0.14	133.0 ± 0.10	133.0 ± 0.10	133.4 ± 0.67
C11	103.9 ± 0.39	103.7 ± 0.32	**103.5 ± 0.23**	103.5 ± 0.15	103.5 ± 0.12	103.5 ± 0.09	103.5 ± 0.06	103.5 ± 0.04	103.5 ± 0.05	103.7 ± 0.21
C12	169.2 ± 0.17	**169.1 ± 0.15**	169.0 ± 0.11	169.0 ± 0.07	169.0 ± 0.06	169.0 ± 0.04	169.0 ± 0.03	169.0 ± 0.02	169.0 ± 0.02	168.9 ± 0.09
H1	**7.2 ± 0.04**	7.2 ± 0.03	7.2 ± 0.02	7.1 ± 0.02	7.1 ± 0.01	7.1 ± 0.01	7.1 ± 0.01	7.1 ± 0.01	7.1 ± 0.01	7.1 ± 0.03
H2	**6.0 ± 0.03**	6.0 ± 0.02	6.0 ± 0.01	6.0 ± 0.01	6.0 ± 0.01	6.0 ± 0.01	6.0 ± 0.00	6.0 ± 0.00	6.0 ± 0.00	6.0 ± 0.02
H3	**5.7 ± 0.09**	5.8 ± 0.06	5.8 ± 0.04	5.8 ± 0.03	5.8 ± 0.02	5.8 ± 0.01	5.8 ± 0.01	5.8 ± 0.01	5.8 ± 0.01	5.8 ± 0.05
H4	**3.9 ± 0.19**	3.9 ± 0.14	3.9 ± 0.10	3.9 ± 0.07	3.9 ± 0.06	3.9 ± 0.04	3.9 ± 0.03	3.9 ± 0.02	3.9 ± 0.02	3.8 ± 0.07
H5	**4.0 ± 0.24**	4.0 ± 0.17	3.9 ± 0.10	3.9 ± 0.07	3.9 ± 0.06	3.9 ± 0.04	3.9 ± 0.03	3.9 ± 0.02	3.9 ± 0.02	3.8 ± 0.06
H6	8.6 ± 0.33	**8.7 ± 0.19**	8.8 ± 0.10	8.8 ± 0.07	8.8 ± 0.05	8.8 ± 0.04	8.8 ± 0.03	8.8 ± 0.02	8.8 ± 0.02	8.6 ± 0.10
H7	6.2 ± 0.24	**6.3 ± 0.16**	6.2 ± 0.12	6.3 ± 0.13	6.3 ± 0.09	6.4 ± 0.13	6.4 ± 0.07	6.4 ± 0.06	6.4 ± 0.07	6.0 ± 0.09
H8	**8.0 ± 0.04**	8.0 ± 0.03	8.0 ± 0.02	8.0 ± 0.02	8.0 ± 0.01	8.0 ± 0.01	8.0 ± 0.01	8.0 ± 0.00	8.0 ± 0.01	8.0 ± 0.04
H9	3.6 ± 0.48	3.8 ± 0.37	3.9 ± 0.21	**4.1 ± 0.17**	4.0 ± 0.14	4.0 ± 0.11	4.0 ± 0.07	4.0 ± 0.05	4.0 ± 0.06	3.8 ± 0.15
H10	4.6 ± 0.51	4.2 ± 0.35	4.1 ± 0.19	**3.9 ± 0.13**	4.0 ± 0.11	4.0 ± 0.09	4.0 ± 0.06	4.0 ± 0.04	4.0 ± 0.04	3.8 ± 0.11
H11	**4.9 ± 0.05**	4.9 ± 0.03	4.8 ± 0.02	4.8 ± 0.01	4.8 ± 0.01	4.8 ± 0.01	4.8 ± 0.01	4.8 ± 0.00	4.8 ± 0.00	4.8 ± 0.01

[a]Refer to Figure 1 for atom definitions.
[b]95% confidence intervals using the Central Limit Theorem.
[c]95% confidence intervals using bootstrapping with M = 1000 samples.
[d]Substitute set of conformations taken from neutral single-crystal structures of furosemide from the CSD. Refer to Table 7 and Figure S16.
[e]Mean value ± 95% confidence interval.
[f]**Bold** values indicate when the calculated mean chemical shift value and its standard deviation lie within the 95% confidence interval of that for the value calculated for the largest ensemble of *N* = 1000.
[g]Underlined values indicate when the standard deviation of the calculated chemical shift is less than the experimental error of measurement (^{13}C ± 0.1 ppm, ^{1}H ± 0.2 ppm).

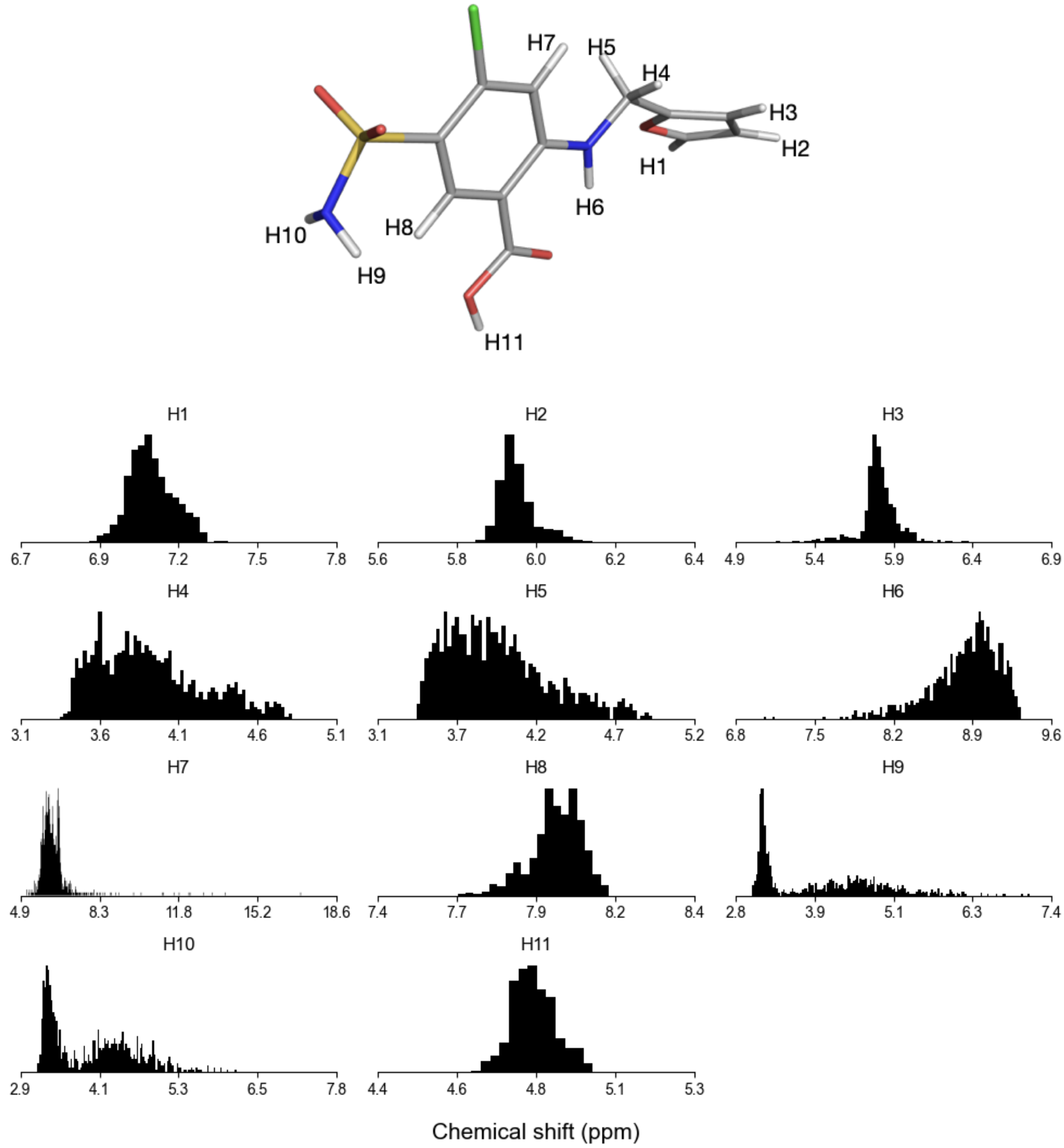

Figure S12A. Distributions of ^{1}H NMR chemical shift values calculated for each nucleus from the N = 1,000 ensemble of conformations randomly selected from the solution dynamic 3D structure. Some nuclei clearly show multi-modal distributions that arise from the modal behaviour of one or more torsions measured in solution (refer to Figure 3 and Table S4), *i.e.,* these nuclei in particular are highly sensitive to conformation (*e.g.,* H4). All bin sizes are 0.025 ppm.

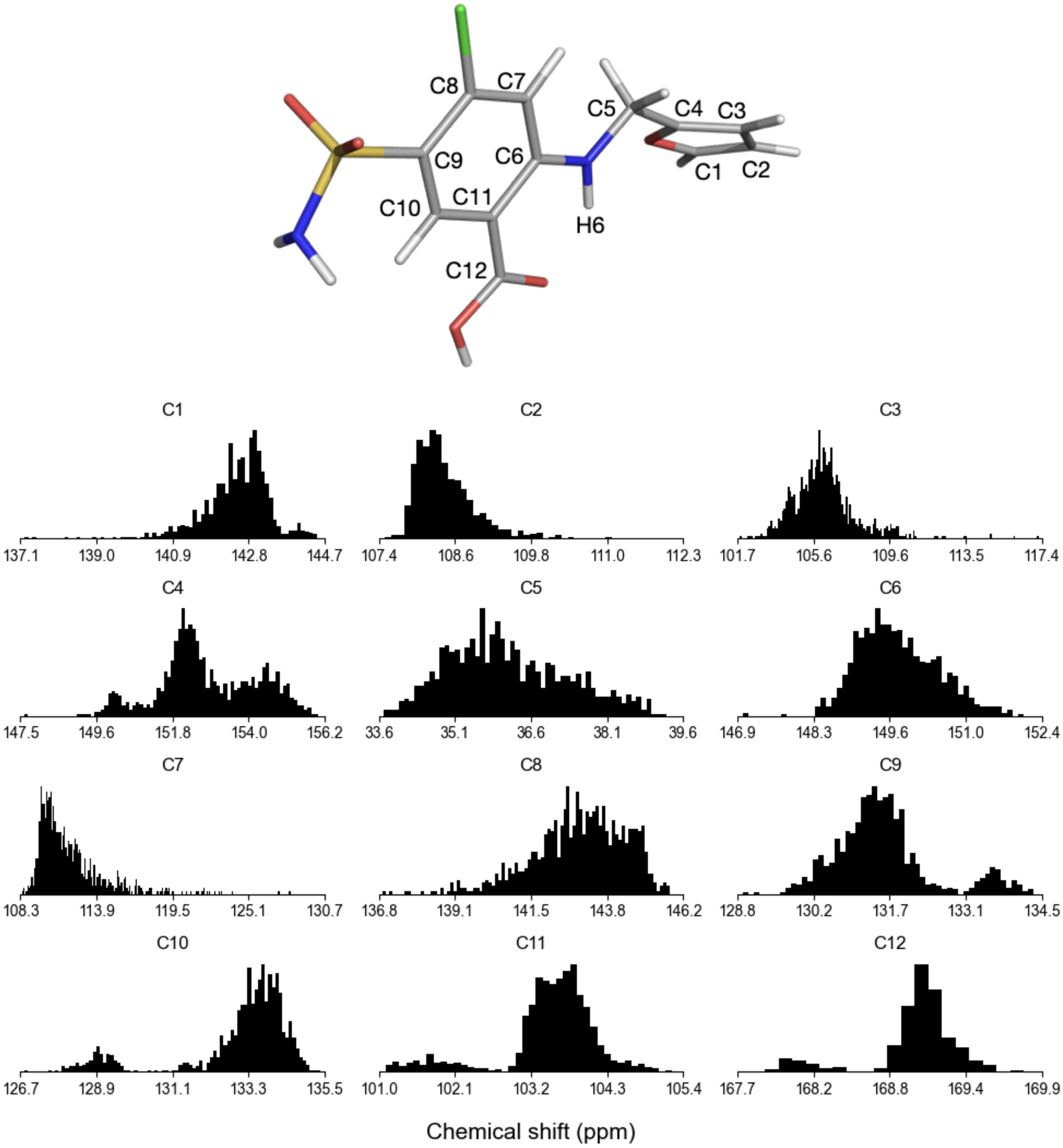

Figure S12B. Distributions of ^{13}C NMR chemical shift values calculated for each nucleus from the N = 1,000 ensemble of conformations randomly selected from the solution dynamic 3D structure. Some nuclei clearly show multi-modal distributions that arise from the modal behaviour of one or more torsions measured in solution (refer to Figure 3 and Table S4), *i.e.,* these nuclei in particular are highly sensitive to conformation (*e.g.,* C4). All bin sizes are 0.1 ppm.

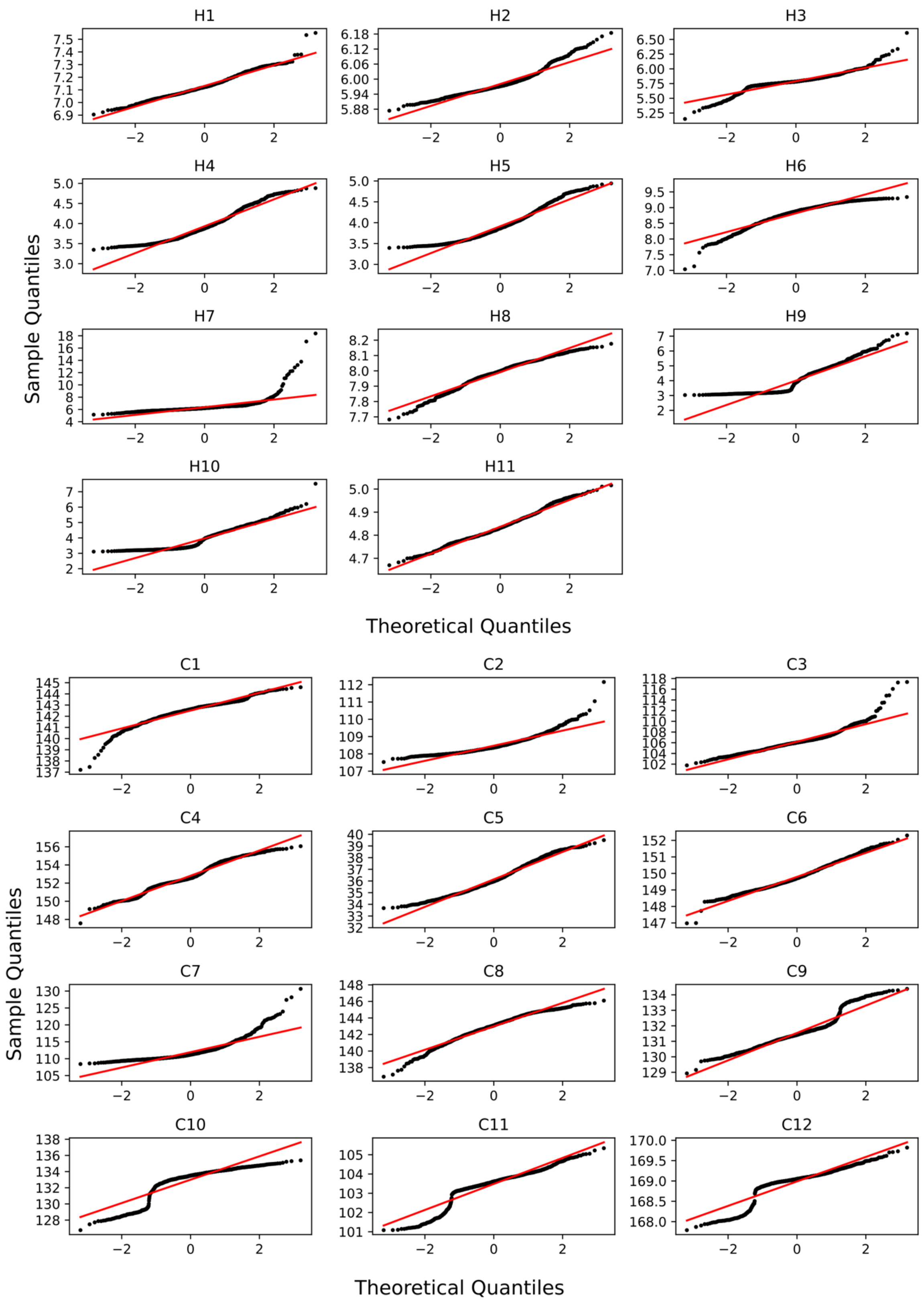

Figure S13A. Quantile-Quantile (Q-Q) plots for each nucleus comparing the distribution of calculated chemical shifts for 1,000 randomly sampled conformations from the solution dynamic 3D structure ensemble (*y*-axis) with a normal distribution (*x*-axis, N = 1,000). Deviations from the $y = x$ identity line indicate where the calculated chemical shift distributions are non-normal (refer to Figure S12). Refer to Figure 1 for atom definitions.

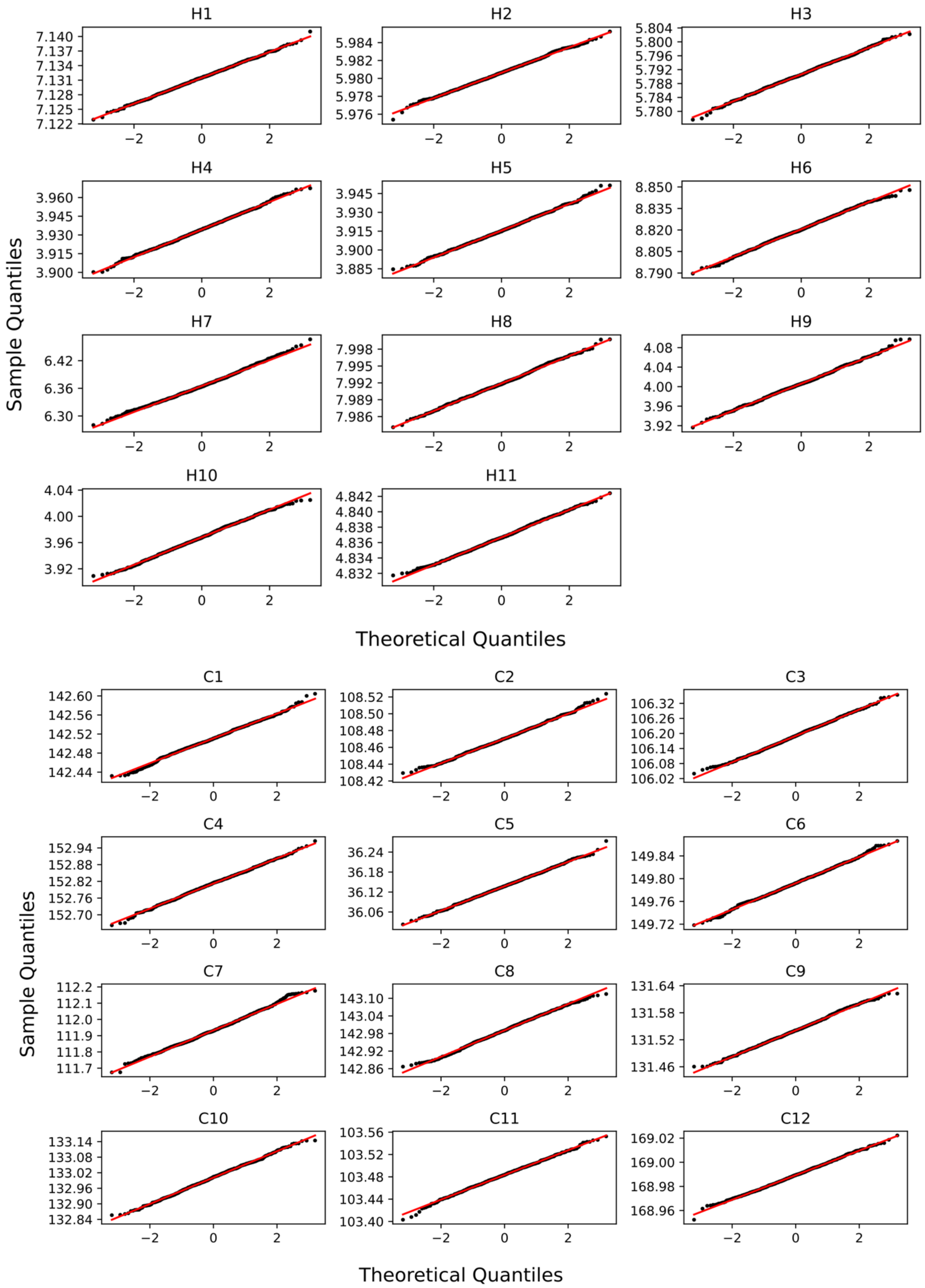

Figure S13B. Quantile-Quantile (Q-Q) plots for each nucleus comparing the distribution of 1,000 mean values calculated from 1,000 conformations randomly resampled using boot-strappping from the solution dynamic 3D structure ensemble (*y*-axis) with a normal distribution (*x*-axis, *N* = 1,000). The close tracking with the identity lines indicates these mean values are normally distributed, *i.e.*, 1,000 conformations are sufficient to sample the dynamic 3D-structure well. Refer to Figure 1 for atom definitions.

3.3 Experimental measurement of solid-state NMR chemical shifts ($\delta_{Solid\ expt}$)

There are no Supporting Information Figures or Tables for this section.

3.4 Calculation of solid-state NMR chemical shifts ($\delta_{Solid\ calc}$)

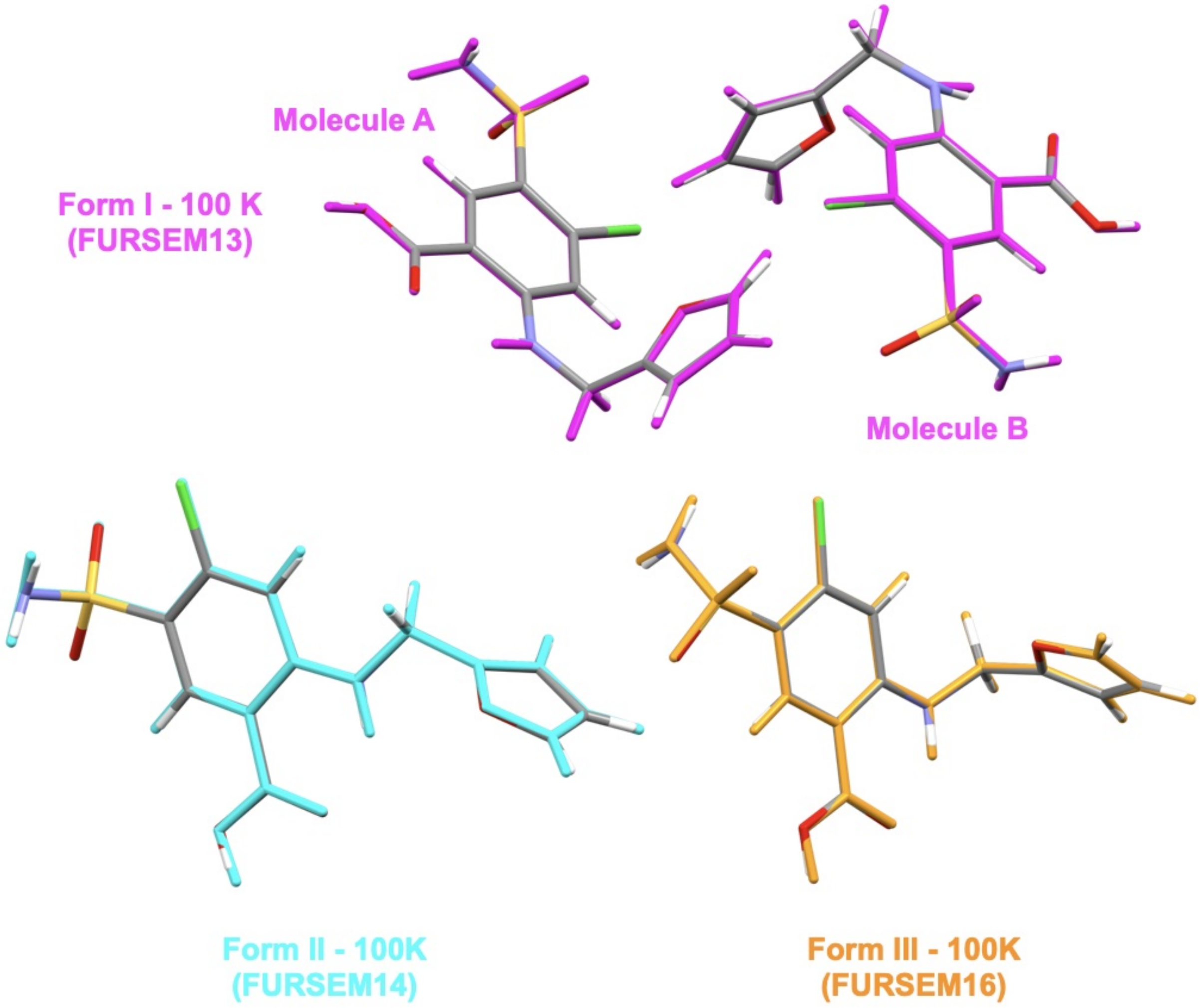

Figure S14. Overlay of the asymmetric units of each furosemide form from diffraction structures obtained at 100 K before (coloured by element) and after (coloured by a block colour) geometry optimisation. All hydrogens are shown.

Table S6. Crystal packing similarity of furosemide crystal structures after DFT geometry optimisation.

Form	CSD entry ID	Temperature (K)	$RMSD_1$[a] (Å)	$RMSD_{15}$ (Å)
I	13[b]	100	0.029	0.035
	18	120	0.037	0.049
	03	173	0.024	0.033
	01	295	0.045	0.068
	17	293	0.144	0.297
	02[c]	295	0.293	0.630[d]
II	14	100	0.029	0.036
	15 (75%)[e]	293	0.039	0.041
	15 (25%)	293	0.058	0.072
III	16	100	0.033	0.034

[a]$RMSD_x$ was calculated using CSD Mercury with the software default settings with a 20% tolerance on distances and 20° tolerance on angle over *x* number of molecules.
[b]CSD FURSEM entry ID (refer to Table 1).
[c]FURSEM02 has no co-ordinates for any hydrogen atoms; these were added in reasonable geometries prior to DFT geometry optimisation.
[d]Only 5 out of 15 molecules matched. In all other structures, 15/15 molecules matched.
[e]FURSEM15 has no co-ordinates for hydrogen atoms on the disordered furan ring; these were added in reasonable geometries prior to DFT geometry optimisation.

Table S7. Torsion angles for each rotatable bond in furosemide crystal structures before and after DFT geometry optimisation (CASTEP).

Form	CSD entry ID		Torsion 1[a] (°)			Torsion 2 (°)			Torsion 3 (°)			Torsion 4 (°)			Torsion 5 (°)			Torsion 6 (°)		
		Mol	CSD[b]	DFT[c]	Δ[d]	CSD	DFT	Δ	CSD	DFT	Δ	CSD	DFT	Δ	CSD	DFT	Δ	CSD	DFT	Δ
Form I	FURSEM13	A	68.2	68.0	–0.2	–84.0	–85.2	–1.2	175.8	175.1	–0.7	166.0	166.5	0.5	172.3	173.5	1.2	76.6	65.9	–10.7
	FURSEM18	A	68.0	66.8	–1.2	–83.8	–85.2	–1.4	175.1	174.7	–0.4	165.4	167.4	2.0	172.4	174.2	1.8	76.7	74.3	–2.4
	FURSEM03	A	67.6	66.7	–0.9	–83.7	–85.6	–1.9	175.5	175.7	0.2	166.0	166.7	0.7	173.1	173.8	0.7	78.8	72.7	–6.1
	FURSEM01	A	67.2	64.4	–2.8	–83.5	–84.1	–0.6	175.2	174.6	–0.6	166.3	167.2	0.9	174.6	173.9	–0.7	–13.4	75.1	88.5
	FURSEM13	B	–57.6	–56.7	0.9	–61.4	–64.0	–2.6	163.6	164.9	1.3	163.2	162.8	–0.4	179.4	–178.5	2.1	85.3	84.1	–1.2
	FURSEM18	B	–57.2	–56.2	1.0	–62.0	–64.0	–2.0	163.9	164.5	0.6	164.0	163.2	–0.8	179.1	–177.5	3.4	82.7	84.4	1.7
	FURSEM03	B	–56.3	–55.6	0.7	–62.1	–64.2	–2.1	164.2	165.7	1.5	164.1	162.8	–1.3	179.5	–179.0	1.5	88.1	82.3	–5.8
	FURSEM01	B	–53.9	–53.5	0.4	–63.0	–63.3	–0.3	165.6	165.2	–0.4	164.9	163.5	–1.4	179.8	–176.1	4.1	–6.9[e]	84.1	91.0
	FURSEM17	A	65.5	60.9	–4.6	–83.1	–92.3	–9.2	174.8	180	5.2	167.1	164.8	–2.3	174.4	158.7	–15.7	0.9[e]	72.2	71.3
	FURSEM17	B	–52.0	–52.8	–0.8	–64.4	–60.4	4.0	166.4	162.3	–4.1	166.3	173.8	7.5	179.9	–169.2	10.9	–0.1[e]	155.2[f]	155.3
	FURSEM02	-	–44.5	–34.2	10.3	–72.6	–58.0	14.6	167.9	170.7	2.8	164.4	168.1	3.7	174.9	–175.7	9.4	0.0[e]	77.0[f]	77.0
Form II	FURSEM14		–78.2	–78.4	–0.2	–166.4	–165.8	0.6	174.3	173.8	–0.5	–79.9	–79.9	0.0	175.2	175.7	0.5	58.2	58.5	0.3
	FURSEM15 (75%)[g]		–80.0	–79.0	1.0	–162.2	–163.6	–1.4	173.4	172.9	–0.5	–78.6	–78.2	0.4	175.4	175.6	0.2	63.0	60.2	–2.8
	FURSEM15 (25%)		97.2	92.3	–0.4	–162.2	–155.3	6.9	173.4	174.6	1.2	–78.6	–77.9	0.7	175.4	175.2	–0.2	63.0	57.9	–5.1
Form III	FURSEM16		–60.0	–60.3	–0.3	91.3	92.8	1.5	–168.8	–168.8	0.0	55.7	54.5	–1.2	176.5	178.7	2.2	69.5	65.2	–4.3

[a]Refer to Figure 1 for torsion definitions.
[b]Value in crystal structure as measured using CSD Mercury before DFT optimisation.
[c]Value as measured using CSD Mercury after DFT optimisation.
[d]Change in value upon DFT optimisation. Positive values denote a clockwise rotation; co-ordinates proceed from –180° to 180°.
[e]Sulphonamide nitrogen geometry is essentially sp^2 before optimisation making assignment of H9 somewhat subjective.
[f]The hydrogen-bond arrangement involving this sulphonamide group is unlike that found in all other Form I crystal structures.
[g]FURSEM15 has disorder around the furan ring, occupying two sites at 75% and 25% occupancy, respectively.

3.5 Linear regression of $\Delta\delta_{Calculated}$ vs $\Delta\delta_{Experimental}$ and t-test to identify the correct form

Table S8. The lower bound of the one-sided 95% confidence intervals for the correlation of $\Delta\delta_{Calculated}$ vs $\Delta\delta_{Experimental}$ (see Table 4).

Form	CSD entry ID	Mol[a]	$\Delta\delta_{Calculated}$ vs $\Delta\delta_{Experimental}$	
			Molecule A	Molecule B
^{13}C				
Form I	13[b]	A	**0.213**[c, d]	–0.174
	18	A	**0.237**	–0.150
	03	A	**0.260**	–0.119
	01	A	**0.302**	–0.075
	13	B	–0.439	**0.232**
	18	B	–0.423	**0.272**
	03	B	–0.393	**0.293**
	01	B	–0.355	**0.297**
	17	A	**–0.077**	–0.050
	17	B	0.572	**–0.123**
	02	-	–0.311	0.471
Form II	14		–0.663	–0.103
	15 (75%)[e]		–0.653	–0.198
	15 (25%)		–0.657	–0.099
Form III	16		–0.270	–0.354
^{1}H				
Form I	13	A	**0.499**	–0.936
	18	A	**0.492**	–0.933
	03	A	**0.602**	–0.935
	01	A	**0.628**	–0.921
	13	B	–0.983	**0.040**
	18	B	–0.980	**0.084**
	03	B	–0.978	**0.115**
	01	B	–0.974	**0.116**
	17	A	**0.010**	–0.878
	17	B	–0.969	**–0.197**
	02	-	–0.474	–0.758
Form II	14		–0.962	–0.404
	15 (75%)[e]		–0.937	–0.568
	15 (25%)		–0.967	–0.385
Form III	16		–0.941	–0.462

[a]Form I has two molecules in the asymmetric unit, which can be readily distinguished by their torsion 1 values (A ≅ 68°, B ≅ –58°). Fit parameters are given for $\Delta\delta_{Calculated}$ vs $\Delta\delta_{Experimental}$ data for either Molecule A or Molecule B treated separately.
[b]CSD FURSEM entry ID (refer to Table 1).
[c]Values are for the fit parameters corresponding to the measured experimental data after omitting the chemical shifts for the ^{1}H atoms in exchange (H6, H9*, and H11) and the ^{13}C atom adjacent to the chlorine (C8).
[d]Values in **bold** indicate the fit parameters for the form corresponding to the measured experimental data, *i.e.,* the ones the approach should identify (see Figures 4 and 5, and Table 4).
[e]FURSEM15 has disorder around the furan ring, occupying two sites at 75% and 25% occupancy, respectively.

Table S9. Linear regression analysis parameters and *p*-values for chemical shift differences between the solution state and the solid state for combinations of calculated (Forms I, II, III) and experimentally measured (Form I, Molecule A and Molecule B) differences in furosemide chemical shift *when data from all the ^{1}H atoms in exchange (H6, H9*, and H11) and the ^{13}C atom adjacent to the chlorine (C8) are included.*

$\Delta\delta_{Calculated}$ for			$\Delta\delta_{Experimental}$ for Form I							
			Molecule A[a]				Molecule B[a]			
Form	CSD entry ID	Mol	r^{2} [b]	m[b]	c[b]	*p*-value	r^{2} [b]	m[b]	c[b]	*p*-value
^{13}C										
Form I	13[c]	A	**0.40**[d]	**0.88**	**0.77**	**0.0138**[e]	0.13	0.74	0.90	0.1291
	18	A	**0.40**	**0.89**	**0.82**	**0.0141**	0.13	0.77	0.93	0.1239
	03	A	**0.41**	**0.88**	**0.76**	**0.0120**	0.15	0.80	0.83	0.1063
	01	A	**0.44**	**0.90**	**0.76**	**0.0091**	0.18	0.86	0.80	0.0859
	13	B	0.01	0.12	1.89	0.4015	**0.35**	**1.28**	**0.81**	**0.0214**
	18	B	0.01	0.14	1.76	0.3813	**0.37**	**1.29**	**0.69**	**0.0180**
	03	B	0.02	0.18	1.53	0.3406	**0.39**	**1.28**	**0.52**	**0.0147**
	01	B	0.03	0.23	1.53	0.2950	**0.40**	**1.27**	**0.57**	**0.0139**
	17	A	**0.21**	**0.64**	**1.07**	**0.0651**	0.23	0.99	0.74	0.0564
	17	B	0.01	–0.12	1.07	0.6026	**0.13**	**0.75**	**0.26**	**0.1217**
	02		0.06	0.33	1.23	0.2303	0.58	1.59	0.06	0.0021
Form II	14		0.04	–0.40	2.49	0.7411	0.20	1.31	0.91	0.0717
	15 (75%)[f]		0.04	–0.33	2.18	0.7231	0.14	0.95	1.00	0.1192
	15 (25%)		0.04	–0.35	2.44	0.7297	0.20	1.22	0.98	0.0699
Form III	16		0.07	0.37	1.54	0.1956	0.03	0.37	1.54	0.2863
^{1}H										
Form I	13	A	**0.02**	**–0.71**	**0.93**	**0.651**	0.20	–3.91	–0.47	0.8886
	18	A	**0.02**	**–0.75**	**0.94**	**0.656**	0.20	–3.98	–0.48	0.8871
	03	A	**0.03**	**–0.78**	**0.92**	**0.661**	0.20	–3.98	–0.49	0.8859
	01	A	**0.02**	**–0.80**	**0.99**	**0.655**	0.18	–4.06	–0.44	0.8743
	13	B	0.22	–2.29	0.48	0.897	**0.06**	**–2.18**	**0.30**	**0.7343**
	18	B	0.21	–2.30	0.49	0.891	**0.06**	**–2.27**	**0.28**	**0.7366**
	03	B	0.20	–2.26	0.49	0.886	**0.06**	**–2.34**	**0.23**	**0.7433**
	01	B	0.19	–2.30	0.57	0.877	**0.06**	**–2.47**	**0.27**	**0.7436**
	17	A	**0.01**	**–0.24**	**0.43**	**0.598**	0.21	–2.10	–0.37	0.8945
	17	B	0.23	–1.48	0.18	0.903	**0.04**	**–1.13**	**0.17**	**0.6950**
	02		0.07	–1.41	0.89	0.7514	0.13	–3.60	-0.16	0.8301
Form II	14		0.25	–2.42	0.20	0.915	0.07	–2.27	0.02	0.7467
	15 (75%)[f]		0.21	–2.24	0.41	0.893	0.08	–2.55	0.06	0.7703
	15 (25%)		0.26	–2.35	0.20	0.917	0.06	–2.09	0.07	0.7365
Form III	16		0.14	–1.73	0.55	0.836	0.09	–2.62	0.01	0.7870

[a]Form I has two molecules in the asymmetric unit, which can be readily distinguished by their torsion 1 values (A ≅ 68°, B ≅ –58°). Fit parameters are given for $\Delta\delta_{Calculated}$ vs $\Delta\delta_{Experimental}$ data for either Molecule A or Molecule B treated separately.

[b]Values are for the fit parameters corresponding to the measured experimental data *when all the ^{1}H and ^{13}C data are used, including all the ^{1}H atoms in exchange (H6, H9*, and H11) and the ^{13}C atom adjacent to the chlorine (C8) are included.*

[c]CSD FURSEM entry ID (refer to Table 1).

[d]Values in **bold** indicate the fit parameters for the form corresponding to the measured experimental data, *i.e.,* the ones the approach should identify.

[e]*p*-values are for the null hypothesis that $m = 0$, and the alternative hypothesis $m > 0$. Values underlined reject the null hypothesis at a one-tailed significance level of 0.050, suggesting a significant positive correlation between $\Delta\delta_{Experimental}$ and $\Delta\delta_{Calculated}$. The lower bound of the one-sided 95% confidence intervals for the correlation between $\Delta\delta_{Experimental}$ and $\Delta\delta_{Calculated}$ are given in Table S10.

[f]FURSEM15 has disorder around the furan ring, occupying two sites at 75% and 25% occupancy, respectively.

Table S10. The lower bound of the one-sided 95% confidence intervals for the correlation of $\Delta\delta_{Experimental}$ vs $\Delta\delta_{Calculated}$ *when data from all the* ^{1}H *atoms in exchange (H6, H9*, and H11) and the* ^{13}C *atom adjacent to the chlorine (C8) are included* (see Table S9).

Form	CSD entry ID	Mol[a]	$\Delta\delta_{Experimental}$ *vs* $\Delta\delta_{Calculated}$ for Form I	
			Molecule A	Molecule B
^{13}C				
Form I	13[b]	A	**0.193**[c, d]	–0.176
	18	A	**0.191**	–0.168
	03	A	**0.212**	–0.138
	01	A	**0.249**	–0.098
	13	B	–0.436	**0.131**
	18	B	–0.422	**0.156**
	03	B	–0.393	**0.185**
	01	B	–0.357	**0.192**
	17	A	**–0.048**	–0.023
	17	B	–0.560	**–0.164**
	02	-	–0.299	0.419
Form II	14		–0.640	–0.065
	15 (75%)[e]		–0.630	–0.160
	15 (25%)		–0.634	–0.060
Form III	16		–0.262	–0.349
^{1}H				
Form I	13	A	**–0.677**	–0.820
	18	A	**–0.680**	–0.819
	03	A	**–0.683**	–0.818
	01	A	**–0.680**	–0.810
	13	B	–0.827	**–0.725**
	18	B	–0.822	**–0.726**
	03	B	–0.819	**–0.730**
	01	B	–0.812	**–0.730**
	17	A	**–0.647**	–0.825
	17	B	–0.831	**–0.702**
	02	-	–0.735	–0.782
Form II	14		–0.840	–0.732
	15 (75%)[e]		–0.824	–0.746
	15 (25%)		–0.842	–0.726
Form III	16		–0.785	–0.756

[a]Form I has two molecules in the asymmetric unit, which can be readily distinguished by their torsion 1 values (A ≅ 68°, B ≅ –58°). Fit parameters are given for $\Delta\delta_{Calculated}$ vs $\Delta\delta_{Experimental}$ data for either Molecule A or Molecule B treated separately.
[b]CSD FURSEM entry ID (refer to Table 1).
[c]Values are for the fit parameters corresponding to the measured experimental data *when all the* ^{1}H *and* ^{13}C *data are used, including all the* ^{1}H *atoms in exchange (H6, H9*, and H11) and the* ^{13}C *atom adjacent to the chlorine (C8) are included.*
[d]Values in **bold** indicate the fit parameters for the form corresponding to the measured experimental data, *i.e.,* the ones the approach should identify.
[e]FURSEM15 has disorder around the furan ring, occupying two sites at 75% and 25% occupancy, respectively.

Table S11. Linear regression analysis parameters and *p*-values for chemical shift differences between the solution state and the solid state for calculated and experimentally measured differences in chemical shift for Form I structures (both Molecules A and B treated together).

$\Delta\delta_{Calculated}$ for		$\Delta\delta_{Experimental}$ for Form I			
Form I	CSD entry ID	r^{2} [a]	m[a]	c[a]	*p*-value
^{13}C	13[b]	**0.42**[c]	**1.04**	**1.12**	**0.00051**[d]
	18	**0.45**	**1.06**	**1.12**	**0.00031**
	03	**0.47**	**1.04**	**0.98**	**0.00022**
	01	**0.49**	**1.05**	**1.00**	**0.00014**
	17	**0.20**	**0.70**	**0.86**	**0.01913**
^{1}H	13	**0.64**	**0.95**	**0.26**	**0.00095**
	18	**0.65**	**0.95**	**0.24**	**0.00074**
	03	**0.69**	**0.93**	**0.20**	**0.00040**
	01	**0.71**	**0.95**	**0.23**	**0.00031**
	17	**0.46**	**0.70**	**0.08**	**0.00750**

[a]Values are for the fit parameters corresponding to the measured experimental data after omitting the chemical shifts for the ^{1}H atoms in exchange (H6, H9*, and H11) and the ^{13}C atom adjacent to the chlorine (C8).
[b]CSD FURSEM entry ID (refer to Table 1).
[c]Values in **bold** indicate the fit parameters for the form corresponding to the measured experimental data, *i.e.,* the ones the approach should identify.
[d]*p*-values are for the null hypothesis that $m = 0$, and the alternative hypothesis $m > 0$. Values underlined reject the null hypothesis at a one-tailed significance level of 0.050, suggesting a significant positive correlation between $\Delta\delta_{Experimental}$ and $\Delta\delta_{Calculated}$.

Table S12. Linear regression analysis parameters and *p*-values for chemical shift differences between the solution state and the solid state for calculated and experimentally measured differences in chemical shift for Form I structures (both Molecules A and B treated together) *when data from all the ^{1}H atoms in exchange (H6, H9*, and H11) and the ^{13}C atom adjacent to the chlorine (C8) are included*.

$\Delta\delta_{Calculated}$ for		$\Delta\delta_{Experimental}$ for Form I			
Form I	CSD entry ID	r^2 [a]	m[a]	c[a]	*p*-value
^{13}C	13[b]	**0.36**[d]	**1.00**	**0.86**	**0.00099**[e]
	18	**0.37**	**1.01**	**0.83**	**0.00081**
	03	**0.39**	**1.00**	**0.71**	**0.00056**
	01	**0.41**	**1.01**	**0.73**	**0.00038**
	17	**0.17**	**0.67**	**0.69**	**0.02270**
^{1}H	13	**0.03**	**–1.04**	**0.80**	**0.75627**
	18	**0.03**	**–1.09**	**0.80**	**0.76107**
	03	**0.03**	**–1.13**	**0.77**	**0.76830**
	01	**0.03**	**–1.17**	**0.84**	**0.76445**
	17	**0.02**	**–0.45**	**0.41**	**0.69737**

[a]Values are for the fit parameters corresponding to the measured experimental data *when all the ^{1}H and ^{13}C data are used, including all the ^{1}H atoms in exchange (H6, H9*, and H11) and the ^{13}C atom adjacent to the chlorine (C8) are included.*
[b]CSD FURSEM entry ID (refer to Table 1).
[c]Values in **bold** indicate the fit parameters for the form corresponding to the measured experimental data, *i.e.,* the ones the approach should identify.
[d]*p*-values are for the null hypothesis that $m = 0$, and the alternative hypothesis $m > 0$. Values underlined reject the null hypothesis at a one-tailed significance level of 0.050, suggesting a significant positive correlation between $\Delta\delta_{Experimental}$ and $\Delta\delta_{Calculated}$.

3.6 Analysis 1: Comparison with the RMSE method

Table S13. Linear regression analysis parameters and RMSE values for combinations of calculated (Forms I, II, III) and experimentally measured (Form I, Molecule A and Molecule B) solid-state NMR chemical shifts.

$\delta_{Solid\ calc}$ for			$\delta_{Solid\ expt}$ for Form I							
			Molecule A[a]				Molecule B[a]			
Form	CSD entry ID	Mol	r^{2} [b]	m[b]	c[b]	RMSE	r^{2} [b]	m[b]	c[b]	RMSE
^{13}C										
Form I	13[c]	A	**1.00**[d]	**1.02**	**–2.54**	**1.85**[e]	0.99	1.03	–4.14	2.43
	18	A	**1.00**	**1.02**	**–2.38**	**1.69**	1.00	1.03	–3.98	2.32
	03	A	**1.00**	**1.02**	**–2.73**	**1.70**	1.00	1.03	–4.35	2.27
	01	A	**1.00**	**1.02**	**–3.01**	**1.71**	1.00	1.04	–4.64	2.25
	13	B	0.99	1.01	–0.70	3.00	**1.00**	**1.02**	**–2.76**	**1.85**
	18	B	0.99	1.01	–0.86	2.88	**1.00**	**1.02**	**–2.91**	**1.69**
	03	B	0.99	1.01	–1.41	2.84	**1.00**	**1.03**	**–3.45**	**1.71**
	01	B	0.99	1.01	–1.51	2.80	**1.00**	**1.03**	**–3.53**	**1.77**
	17	A	**0.99**	**1.01**	**–0.79**	**2.56**	0.99	1.02	–2.53	2.54
	17	B	0.99	0.99	0.37	3.10	**1.00**	**1.01**	**–1.63**	**2.15**
	02		0.99	1.01	–1.03	2.95	1.00	1.02	–3.05	1.95
Form II	14		0.97	0.99	1.37	5.34	0.98	1.01	–0.90	4.37
	15 (75%)[f]		0.98	0.99	1.42	4.83	0.99	1.01	–0.73	3.99
	15 (25%)		0.98	1.00	0.66	5.17	0.98	1.02	–1.59	4.23
Form III	16		0.99	1.06	–7.30	3.16	0.99	1.07	–9.21	2.88
^{1}H										
Form I	13	A	**0.98**	**1.09**	**–0.95**	**0.34**	0.92	1.03	–0.37	0.71
	18	A	**0.98**	**1.11**	**–1.07**	**0.38**	0.92	1.05	–0.49	0.72
	03	A	**0.98**	**1.12**	**–1.14**	**0.37**	0.92	1.06	–0.58	0.70
	01	A	**0.97**	**1.20**	**–1.68**	**0.44**	0.92	1.14	–1.08	0.78
	13	B	0.79	1.05	–0.55	1.18	**0.97**	**1.12**	**–1.02**	**0.48**
	18	B	0.80	1.09	–0.82	1.19	**0.97**	**1.16**	**–1.27**	**0.49**
	03	B	0.81	1.09	–0.86	1.16	**0.97**	**1.16**	**–1.28**	**0.47**
	01	B	0.82	1.15	–1.27	1.20	**0.97**	**1.22**	**–1.65**	**0.52**
	17	A	**0.84**	**0.54**	**2.56**	**0.52**	0.78	0.51	2.84	0.61
	17	B	0.68	0.74	1.20	1.12	**0.84**	**0.80**	**0.82**	**0.80**
	02		0.93	1.26	–2.05	0.74	0.96	1.24	–1.78	0.61
Form II	14		0.72	0.95	–0.04	1.31	0.91	1.04	–0.61	0.75
	15 (75%)[f]		0.75	1.00	–0.32	1.29	0.90	1.07	–0.74	0.82
	15 (25%)		0.72	0.93	0.09	1.27	0.91	1.02	–0.48	0.70
Form III	16		0.88	1.09	–0.95	0.90	0.98	1.12	–1.06	0.40

[a]Form I has two molecules in the asymmetric unit, which can be readily distinguished by their torsion 1 values (A ≅ 68°, B ≅ –58°). Fit parameters are given for $\Delta\delta_{Calculated}$ vs $\Delta\delta_{Experimental}$ data for either Molecule A or Molecule B treated separately.
[b]Values are for the fit parameters.
[c]CSD FURSEM entry ID (refer to Table 1).
[d]Values in **bold** indicate the fit parameters for the form corresponding to the measured experimental data, *i.e.,* the ones the approach should identify.
[e]RMSE values between $\delta_{Solid\ expt}$ and $\delta_{Solid\ calc}$ as calculated according to reference.[13] Note that in this approach, no ^{1}H or ^{13}C data points are removed due to exchange with solvent or other reasons. Values underlined indicate RMSE values that are within the thresholds for identifying a correct match (^{13}C 1.90 ppm, ^{1}H 0.33 ppm).[13]
[f]FURSEM15 has disorder around the furan ring, occupying two sites at 75% and 25% occupancy, respectively.

3.7 Analysis 2: Effects of allowing unit cell parameters to vary during geometry optimisation

Table S14A. Relative energies and densities of crystal structures of furosemide after DFT geometry optimisation with unit cell parameters being fixed or allowed to vary during optimisation.

Structure			Structure parameters after geometry optimisation			
			Fixed unit cell (DFT)[a]		Variable unit cell (DFT-D)[b]	
Form	CSD entry ID	Z[c]	Relative energy per molecule (kJ/mol)[d]	Density (g/cm^3)	Relative energy per molecule (kJ/mol)[d]	Density (g/cm^3)
Form I	13[e]	4	0.00	1.700	0.00	1.667
	18	4	–1.99	1.689	–0.82	1.662
	03	4	–7.46	1.658	–0.78	1.659
	01	4	–9.46	1.648	–0.77	1.660
	17	4	17.15	1.645	23.63	1.681
	02	2	77.62	1.648	9.67	1.612
Form II	14	4	–7.86	1.637	1.79	1.615
	15 (75%)[f]	4	–13.83	1.594	0.74	1.617
	15 (25%)	4	–12.63	1.594	3.36	1.615
Form III	16	2	–12.47	1.622	0.11	1.603

[a]The unit cell parameters were kept constant while geometry optimisation was performed using DFT (CASTEP v17.21).
[b]The unit cell parameters were allowed to vary, while geometry optimisation was performed using dispersion correction DFT-D (CASTEP v. 20.21 with pseudopotential from v. 17.21).
[c]Refers to the number of molecules in the unit cell.
[d]Energies are given relative to the structure of FURSEM13 (Form 1, 100 K), which are –22178.35 eV/molecule and –22187.44 eV/molecule for fixed and variable cell DFT-D geometry optimisation, respectively.
[e]CSD FURSEM entry ID (refer to Table 1).
[f]FURSEM15 has disorder around the furan ring, occupying two sites at 75% and 25% occupancy, respectively.

Table S14B. Changes to unit cell parameters for crystal structures of furosemide after DFT geometry optimisation with unit cell parameters being fixed or allowed to vary (with DFT-D) during optimisation.

Structure			Structure parameters after geometry optimisation			
			Fixed unit cell (DFT)[a]		Variable unit cell (DFT-D)[b]	
Form	CSD entry ID	Z[c]	Cell lengths (a, b, c) / Å	Cell angles (α, β, γ) / °	Cell lengths (a, b, c) / Å	Cell angles (α, β, γ) / °
Form I	13[d]	4	9.515, 10.448, 15.583	92.84, 107.09, 116.75	9.659, 10.434, 15.675	92.51, 107.66, 116.43
	18	4	9.535, 10.469, 15.606	92.94, 107.09, 116.62	9.654, 10.467, 15.724	92.32, 107.86, 116.64
	03	4	9.593, 10.504, 15.710	93.06, 107.22, 116.21	9.663, 10.441, 15.742	92.59, 107.87, 116.29
	01	4	9.584, 10.467, 15.725	93.47, 107.27, 115.04	9.668, 10.424, 15.699	93.00, 107.64, 115.99
	17	4	9.590, 10.475, 15.735	93.46, 107.31, 115.04	9.408, 10.137, 15.755	96.03, 104.41, 112.83
	02	2	5.234, 8.751, 14.982	77.43, 89.10, 84.41	5.265, 9.086, 14.819	102.71, 91.61, 99.09
Form II	14	4	5.010, 10.109, 26.620	90.00, 95.40, 90.00	5.019, 10.144, 26.825	90.00, 95.20, 90.00
	15 (75%)[e]	4	5.031, 10.254, 28.803	90.00, 94.70, 90.00	4.949, 10.197, 27.014	90.00, 94.94, 90.00
	15 (25%)	4	5.031, 10.254, 26.803	90.00, 94.70, 90.00	4.970, 10.204, 26.939	90.00, 95.23, 90.00
Form III	16	2	4.876, 10.500, 13.641	78.07, 86.72, 82.59	4.931, 10.456, 13.650	79.24, 86.22, 82.84

[a]The unit cell parameters were kept constant while geometry optimisation was performed using DFT (CASTEP v17.21).
[b]The unit cell parameters were allowed to vary, while geometry optimisation was performed using dispersion correction DFT-D (CASTEP v. 20.21 with pseudopotentials from v. 17.21).
[c]Refers to the number of molecules in the unit cell.
[d]CSD FURSEM entry ID (refer to Table 1).
[e]FURSEM15 has disorder around the furan ring, occupying two sites at 75% and 25% occupancy, respectively.

Table S15. Crystal packing similarity between crystal structures of furosemide after DFT geometry optimisation with unit cell parameters being fixed or allowed to vary during optimisation, as calculated using the crystal packing similarity tool within Mercury using default settings.

			Form I							
			Fixed unit cell (DFT)[a]				Variable unit cell (DFT-D)[b]			
			13[c] 100 K	18 120 K	03 173 K	01 295 K	13 100 K	18 120 K	03 173 K	01 295 K
Form I	13	100 K	-	0.034[d]	0.086	0.141	-	0.033	0.035	0.049
	18	120 K	0.034	-	0.071	0.121	0.033	-	0.028	0.065
	03	173 K	0.086	0.071	-	0.079	0.035	0.028	-	0.046
	01	295 K	0.141	0.121	0.079	-	0.049	0.065	0.046	-

[a]The unit cell parameters were kept constant while geometry optimisation was performed using DFT (CASTEP v17.21).
[b]The unit cell parameters were allowed to vary, while geometry optimisation was performed using dispersion correction DFT-D (CASTEP v. 20.21 with pseudopotential from v. 17.21).
[c]CSD FURSEM entry ID (refer to Table 1).
[d]RMSD for 15 molecules (Å).

Table S16. Experimentally measured and calculated GIPAW NMR chemical shifts for furosemide in solution and in the solid-state forms *after allowing the unit cell to vary during DFT-D geometry optimisation.*

Nucleus	Experimentally measured δ (ppm)			Calculated δ (ppm)															
	$\delta_{\text{Solution expt}}$[a]	$\delta_{\text{Solid expt}}$[b]		$\delta_{\text{Solution calc}}$[c]	$\delta_{\text{Solid calc}}$[d]														
		Form I, Molecule			Form I, Molecule											Form II			Form III
		A[e]	B[e]		13[f] A[e]	18 A	03 A	01 A	13 B[e]	18 B	03 B	01 B	17 A	17 B	02	14	15 (75%)[g]	15 (25%)	16
C1	143.3[h]	141.5[h]	144.2	142.5	142.4	142.5	142.5	142.4	145.6	145.5	145.4	145.3	143.7	144.4	143.9	148.3	146.9	148.3	145.0
C2	111.1	112.1	110.5	108.5	112.9	112.6	112.6	112.5	110.0	110.0	110.1	110.2	112.3	110.5	109.6	113.7	114.7	113.8	111.5
C3	108.2	109.4	110.5	106.2	110.5	110.5	110.5	110.3	112.5	112.3	112.3	112.3	110.3	113.6	111.2	113.7	112.1	114.1	110.9
C4	151.9	155.2	150.6	152.8	155.1	155.1	155.0	154.8	152.2	152.2	152.3	152.4	153.7	152.7	151.4	148.0	147.0	147.2	154.8
C5	39.7	39.6	39.6	36.1	37.1	37.0	37.1	37.2	36.4	36.4	36.4	36.5	37.8	37.7	37.8	38.0	39.2	38.7	38.4
C6	152.9	155.2	154.0	149.8	152.0	152.0	152.0	152.1	150.8	150.9	150.9	150.9	152.1	150.7	151.7	147.4	148.7	148.2	152.3
C7	114.1	116.8	117.1	111.9	115.9	115.9	116.0	116.1	117.0	116.9	116.7	116.8	115.9	117.0	116.9	111.7	111.7	111.7	112.6
C8	136.7	138.0	138.0	143.0	142.3	142.2	142.3	142.2	141.5	141.7	141.7	141.8	145.1	140.5	142.2	146.2	146.3	146.3	143.9
C9	127.3	125.2	127.1	131.5	126.7	126.8	126.7	126.6	128.9	129.1	128.8	128.9	125.2	126.6	128.9	131.0	130.8	131.1	134.7
C10	133.8	136.6	135.4	133.0	135.9	135.8	135.9	135.9	134.4	134.4	134.3	134.3	135.3	131.4	134.4	133.5	134.7	134.2	135.9
C11	108.7	106.1	108.6	103.5	105.1	105.2	105.1	104.9	107.7	107.7	107.5	107.5	105.1	107.8	106.8	104.5	105.8	104.7	106.1
C12	169.2	172.2	172.2	169.0	173.3	173.4	173.3	173.5	173.6	173.7	173.7	173.8	172.3	168.2	174.0	174.8	174.8	175.1	175.0
H1	7.7	6.5	7.7	7.1	6.5	6.4	6.4	6.4	7.8	7.8	7.7	7.7	6.6	7.7	7.0	7.7	8.0	7.7	7.4
H2	6.5	6.4	6.0	6.0	6.5	6.4	6.4	6.5	6.0	6.0	6.0	5.9	6.4	6.2	5.2	6.7	7.3	6.7	6.2
H3	6.4	5.7	6.0	5.8	5.1	5.1	5.1	5.0	5.9	5.9	5.8	5.8	4.8	5.9	5.3	5.8	5.7	5.7	6.0
H4*[i]	4.7	4.7	4.3	3.9	4.2	4.2	4.2	4.2	3.9	3.9	3.9	4.0	4.5	4.0	4.4	3.8	3.6	3.9	3.7
H6	8.7	8.4	8.4	8.8	7.9	7.9	8.0	8.0	8.4	8.4	8.5	8.5	7.4	9.0	8.5	8.0	8.3	8.0	8.4
H7	7.1	7.9	6.2	6.4	7.3	7.3	7.3	7.3	4.9	4.9	4.9	4.9	6.9	4.8	6.5	4.4	4.9	4.4	5.8
H8	8.5	8.7	8.6	8.0	8.0	8.0	8.0	8.1	7.9	7.9	7.9	7.9	8.3	7.7	8.1	7.2	7.3	7.3	8.1
H9*[j]	7.4	6.5	6.7	4.0	6.5	6.5	6.5	6.5	7.1	7.1	7.1	7.1	6.3	5.8	7.0	6.7	6.8	6.8	6.0
H11	13.4	12.7	12.7	4.8	13.8	13.7	13.8	13.9	14.0	14.0	14.0	14.1	10.0	10.3	14.2	13.1	12.9	13.3	13.5

[a]Chemical shifts of furosemide in DMSO-d_6 at 10 mM and 25 ºC, referenced relative to absolute TMS in $CDCl_3$ as follows: Direct (^{1}H) and indirect (^{13}C) referencing relative to internal d_6-DSS, then corrections of ^{1}H +0.0455 and ^{13}C –2.6194 (see sections 2.2 and 2.3). Raw values are given in Table S22.
[b]Data taken from Widdifield *et al.*[14] (see section 2.4).
[c]Solution-state NMR chemical shifts were calculated as described in sections 2.5 and 2.6.
[d]Solid-state NMR chemical shifts were calculated as described in sections 2.5 and 2.7.
[e]Form I has two molecules in the asymmetric unit, which can be readily distinguished by their torsion 1 values (A ≅ 68°, B ≅ −58°).
[f]CSD FURSEM entry ID (refer to Table 1).
[g]FURSEM15 has disorder around the furan ring, occupying two sites at 75% and 25% occupancy, respectively.
[h]Measurement errors are in ppm. In solution: ^{1}H ± 0.001, ^{13}C ± 0.020; in the solid-state: ^{1}H ± 0.2, ^{13}C ± 0.1.[15, 16]
[i]H4 and H5 have identical chemical shifts in solution due to the absence of a chiral centre in the molecule, manifesting as a single resonance, labelled H4*. The solid-state NMR chemical shifts are given as the mean of H4 and H5.
[j]The sulphonamide hydrogens (H9, H10) are in rapid exchange in solution and manifest in spectra as a single broadened triplet resonance, labelled H9*. The solid-state NMR chemical shifts are given as the mean of H9 and H10.

Table S17. Comparison of the experimentally measured ($\Delta\delta_{Experimental}$) and GIPAW calculated ($\Delta\delta_{Calculated}$) differences in NMR chemical shifts for furosemide between the solution state and the solid-state forms *after allowing the unit cell to vary during DFT-D geometry optimisation.*

Nucleus	Experimentally measured change $\Delta\delta_{Experimental}$[a]		Calculated change $\Delta\delta_{Calculated}$[b]														
	Form I, Molecule[c]		Form I, Molecule											Form II			Form III
	A	B	13 A[d]	18 A	03 A	01 A	13 B	18 B	03 B	01 B	17 A	17 B	02	14	15 (75%)[e]	15 (25%)	16
C1	–1.75[f]	0.94	–0.07	–0.01	–0.02	–0.09	3.07	3.03	2.87	2.79	1.14	1.92	1.41	5.82	4.37	5.76	2.52
C2	1.00	–0.56	4.38	4.17	4.15	3.99	1.51	1.51	1.58	1.69	3.82	2.07	1.09	5.25	6.19	5.31	3.05
C3	1.19	2.35	4.28	4.34	4.32	4.14	6.35	6.11	6.11	6.15	4.15	7.37	5.01	7.51	5.90	7.94	4.71
C4	3.33	–1.27	2.31	2.29	2.20	2.00	–0.61	–0.58	–0.53	–0.45	0.88	–0.08	–1.41	–4.83	–5.79	–5.59	1.98
C5	–0.06	–0.06	1.00	0.90	1.00	1.06	0.30	0.29	0.29	0.35	1.62	1.60	1.69	1.85	3.08	2.61	2.26
C6	2.29	1.09	2.21	2.23	2.25	2.28	1.04	1.12	1.07	1.13	2.35	0.89	1.95	–2.38	–1.14	–1.64	2.53
C7	2.69	2.99	4.00	3.99	4.04	4.21	5.08	4.92	4.78	4.83	3.95	5.10	4.98	–0.20	–0.26	–0.25	0.69
C8	1.30	1.30	–0.72	–0.77	–0.67	–0.78	–1.49	–1.33	–1.30	–1.16	2.11	–2.49	–0.77	3.17	3.32	3.26	0.92
C9	–2.11	–0.21	–4.79	–4.73	–4.82	–4.91	–2.60	–2.43	–2.69	–2.62	–6.37	–4.92	–2.68	–0.58	–0.78	–0.44	3.13
C10	2.75	1.51	2.95	2.82	2.89	2.95	1.35	1.38	1.26	1.33	2.27	–1.56	1.38	0.48	1.68	1.16	2.92
C11	–2.63	–0.08	1.64	1.68	1.65	1.43	4.23	4.20	4.06	3.98	1.58	4.28	3.29	1.05	2.31	1.23	2.61
C12	3.09	3.09	4.31	4.37	4.35	4.46	4.66	4.67	4.72	4.79	3.26	-0.80	4.99	5.85	5.78	6.11	5.98
H1	–1.20[f]	0.00	–0.68	–0.70	–0.72	–0.73	0.69	0.66	0.61	0.53	–0.56	0.57	–0.11	0.62	0.82	0.59	0.24
H2	–0.09	–0.49	0.50	0.45	0.42	0.48	0.00	0.01	–0.02	–0.04	0.44	0.24	–0.77	0.74	1.30	0.69	0.25
H3	–0.74	–0.44	–0.67	–0.66	–0.68	–0.76	0.12	0.06	0.04	0.03	–1.03	0.09	–0.48	–0.03	–0.06	–0.08	0.25
H4*[g]	0.04	–0.36	0.28	0.29	0.30	0.32	–0.03	–0.05	0.00	0.05	0.57	0.11	0.49	–0.14	–0.28	–0.05	–0.23
H6	–0.31	–0.31	–0.91	–0.89	–0.85	–0.81	–0.41	–0.38	–0.33	–0.28	–1.40	0.21	–0.37	–0.84	–0.50	–0.83	–0.40
H7	0.77	–0.93	0.95	0.91	0.93	0.92	–1.48	–1.46	–1.46	–1.48	0.56	–1.52	0.10	–1.98	–1.46	–1.98	–0.55
H8	0.23	0.13	0.02	0.00	0.03	0.09	–0.10	–0.08	–0.09	–0.06	0.27	–0.31	0.09	–0.75	–0.71	–0.65	0.07
H9*[h]	–0.91	–0.71	2.50	2.48	2.54	2.46	3.11	3.09	3.10	3.16	2.28	1.80	3.03	2.67	2.80	2.81	2.04
H11	–0.66	–0.66	8.94	8.91	8.99	9.11	9.19	9.13	9.21	9.24	5.13	5.50	9.38	8.30	8.07	8.45	8.67

[a]Difference between experimentally measured solid-state and solution-state NMR chemical shifts as per equation 1 ($\Delta\delta_{Experimental} = \delta_{Solid\ exp} - \delta_{Solution\ expt}$).

[b]Difference between GIPAW-calculated solid-state and solution-state NMR chemical shifts as per equation 2 ($\Delta\delta_{Calculated} = \delta_{Solid\ calc} - \delta_{Solution\ calc}$). Here $\delta_{Solid\ calc}$ is replaced, as compared to Table 2, with values from Table S16 for the GIPAW-calculated chemical shifts after allowing the unit cell to vary during DFT-D geometry optimisation.

[c]Form I has two molecules in the asymmetric unit, which can be readily distinguished by their torsion 1 values (A ≅ 68°, B ≅ –58°).

[d]CSD FURSEM entry ID (refer to Table 1).

[e]FURSEM15 has disorder around the furan ring, occupying two sites at 75% and 25% occupancy, respectively.

[f]Errors are ^{1}H ± 0.2 ppm, ^{13}C ± 0.1 ppm.[15, 16]

[g]H4 and H5 have identical chemical shifts in solution due to the absence of a chiral centre in the molecule, manifesting as a single resonance, labelled H4*. The solid-state NMR chemical shifts are given as the mean of H4 and H5.

[h]The sulphonamide hydrogens (H9, H10) are in rapid exchange in solution and manifest in spectra as a single broadened triplet resonance, labelled H9*. The solid-state NMR chemical shifts are given as the mean of H9 and H10.

Table S18. Linear regression analysis parameters and *p*-values for chemical shift differences between the solution state and the solid state for combinations of calculated (Forms I, II, III) and experimentally measured (Form I, Molecule A and Molecule B) differences in furosemide NMR chemical shift *after allowing the unit cell to vary during DFT-D geometry optimisation.*

$\Delta\delta_{Calculated}$ for			$\Delta\delta_{Experimental}$ for Form I							
			Molecule A[a]				Molecule B[a]			
Form	CSD entry ID	Mol	$r^{2\,b}$	m^b	c^b	*p*-value	$r^{2\,b}$	m^b	c^b	*p*-value
^{13}C										
Form I	13[c]	A	**0.46**[d]	**0.83**	**1.28**	**0.0106**[e]	0.20	0.82	1.29	0.0829
	18	A	**0.46**	**0.82**	**1.28**	**0.0109**	0.22	0.84	1.25	0.0744
	03	A	**0.46**	**0.83**	**1.27**	**0.0109**	0.22	0.86	1.23	0.0717
	01	A	**0.48**	**0.85**	**1.20**	**0.0091**	0.25	0.92	1.14	0.0592
	13	B	0.02	0.17	2.06	0.3388	**0.53**	**1.34**	**1.02**	**0.0056**
	18	B	0.02	0.17	2.05	0.3407	**0.53**	**1.30**	**1.04**	**0.0053**
	03	B	0.03	0.19	1.97	0.3157	**0.52**	**1.30**	**0.98**	**0.0059**
	01	B	0.03	0.21	1.99	0.2977	**0.53**	**1.30**	**1.02**	**0.0054**
	17	A	0.29	0.71	1.06	**0.0439**	0.22	0.92	0.87	0.0738
	17	B	0.00	0.02	1.42	0.4822	0.10	0.74	0.79	**0.1678**
	02		0.07	0.30	1.71	0.2190	0.64	1.37	0.75	0.0016
Form II	14		0.04	−0.37	2.13	0.7317	0.18	1.12	0.81	0.0967
	15 (75%)[f]		0.05	−0.37	2.27	0.7429	0.15	0.97	1.07	0.1202
	15 (25%)		0.04	−0.38	2.35	0.7308	0.20	1.22	0.93	0.0838
Form III	16		0.01	0.05	2.90	0.4016	0.12	0.33	2.65	0.1466
^{1}H										
Form I	13	A	**0.82**	**0.84**	**0.20**	**0.0064**	0.41	−1.10	−0.32	0.9163
	18	A	**0.83**	**0.83**	**0.18**	**0.0055**	0.42	−1.08	−0.33	0.9175
	03	A	**0.85**	**0.85**	**0.19**	**0.0042**	0.40	−1.07	−0.33	0.9104
	01	A	**0.85**	**0.88**	**0.20**	**0.0046**	0.35	−1.04	−0.31	0.8899
	13	B	0.76	−0.89	−0.28	0.9883	**0.58**	**1.44**	**0.37**	**0.0386**
	18	B	0.74	−0.86	−0.28	0.9859	**0.61**	**1.43**	**0.35**	**0.0342**
	03	B	0.72	−0.83	−0.29	0.9836	**0.61**	**1.41**	**0.34**	**0.0340**
	01	B	0.67	−0.79	−0.29	0.9769	**0.62**	**1.41**	**0.33**	**0.0314**
	17	A	0.68	0.79	0.17	**0.0215**	0.08	−0.51	−0.14	0.7094
	17	B	0.69	−0.87	−0.28	0.9794	0.41	1.23	0.29	**0.0856**
	02		0.13	0.23	−0.08	0.2433	0.01	0.14	−0.06	0.4097
Form II	14		0.60	−1.11	−0.44	0.9654	0.23	1.25	0.18	0.1706
	15 (75%)[f]		0.47	−0.98	−0.23	0.9346	0.10	0.85	0.23	0.2668
	15 (25%)		0.58	−1.06	−0.42	0.9611	0.26	1.32	0.21	0.1492
Form III	16		0.61	−0.37	−0.05	0.9675	0.37	0.52	0.19	0.1010

[a]Form I has two molecules in the asymmetric unit, which can be readily distinguished by their torsion 1 values values (A ≅ 68°, B ≅ −58°). Fit parameters are given for $\Delta\delta_{Calculated}$ vs $\Delta\delta_{Experimental}$ data for either Molecule A or Molecule B, treated separately.

[b]Values are for the fit parameters corresponding to the measured experimental data after omitting the chemical shifts for the ^{1}H atoms in exchange (H6, H9*, and H11) and the ^{13}C atom adjacent to the chlorine (C8).

[c]CSD FURSEM entry ID (refer to Table 1).

[d]Values in **bold** indicate the fit parameters for the form corresponding to the measured experimental data, *i.e.,* the ones the approach should identify (see Figures 4 and 5).

[e]*p*-values are for the null hypothesis that $m = 0$, and the alternative hypothesis $m > 0$. Values underlined reject the null hypothesis at a one-tailed significance level of 0.050, suggesting a significant positive correlation between $\Delta\delta_{Calculated}$ and $\Delta\delta_{Experimental}$. The lower bound of the one-sided 95% confidence intervals for the correlation between $\Delta\delta_{Calculated}$ and $\Delta\delta_{Experimental}$ are given in Table S19.

[f]FURSEM15 has disorder around the furan ring, occupying two sites at 75% and 25% occupancy, respectively.

Table S19. The lower bound of the one-sided 95% confidence intervals for the correlation of $\Delta\delta_{Calculated}$ vs $\Delta\delta_{Experimental}$ *after allowing the unit cell to vary during DFT-D geometry optimisation* (see Table S18).

Form	CSD entry ID	Mol[a]	$\Delta\delta_{Experimental}$ *vs* $\Delta\delta_{Calculated}$ for Form I	
			Molecule A	Molecule B
^{13}C				
Form I	13[b]	A	**0.243**[c, d]	–0.098
	18	A	**0.240**	–0.077
	03	A	**0.240**	–0.070
	01	A	**0.265**	–0.034
	13	B	–0.413	**0.329**
	18	B	–0.414	**0.336**
	03	B	–0.394	**0.322**
	01	B	–0.379	**0.333**
	17	A	**0.020**	–0.075
	17	B	–0.513	**–0.244**
	02	-	–0.304	0.476
Form II	14		–0.661	–0.128
	15 (75%)[e]		–0.667	–0.172
	15 (25%)		–0.660	–0.100
Form III	16		–0.459	–0.214
^{1}H				
Form I	13	A	**0.505**	–0.937
	18	A	**0.535**	–0.938
	03	A	**0.582**	–0.934
	01	A	**0.566**	–0.925
	13	B	–0.980	**0.055**
	18	B	–0.978	**0.091**
	03	B	–0.976	**0.092**
	01	B	–0.971	**0.115**
	17	A	**0.221**	–0.847
	17	B	–0.972	**–0.189**
	02	-	–0.519	–0.679
Form II	14		–0.963	–0.408
	15 (75%)[e]		–0.946	–0.548
	15 (25%)		–0.961	–0.366
Form III	16		–0.964	–0.242

[a]Form I has two molecules in the asymmetric unit, which can be readily distinguished by their torsion 1 values (A ≅ 68°, B ≅ –58°). Fit parameters are given for $\Delta\delta_{Calculated}$ vs $\Delta\delta_{Experimental}$ data for either Molecule A or Molecule B treated separately.
[b]CSD FURSEM entry ID (refer to Table 1).
[c]Values are for the fit parameters corresponding to the measured experimental data *after omitting the chemical shift for the ^{1}H atoms in exchange (H6, H9*, and H11) and the ^{13}C atom adjacent to the chlorine (C8).*
[d]Values in **bold** indicate the fit parameters for the form corresponding to the measured experimental data, *i.e.,* the ones the approach should identify.
[e]FURSEM15 has disorder around the furan ring, occupying two sites at 75% and 25% occupancy, respectively.

Table S20. Linear regression analysis parameters and RMSE values for combinations of calculated (Forms I, II, III) and experimentally measured (Form I, Molecule A and Molecule B) solid-state NMR chemical shifts *after allowing the unit cell to vary during DFT-D geometry optimisation.*

$\delta_{Solid\ calc}$ for			$\delta_{Solid\ expt}$ for Form I							
			Molecule A[a]				Molecule B[a]			
Form	CSD entry ID	Mol	r^2 [b]	*m*[b]	c[b]	RMSE	r^2 [b]	*m*[b]	c[b]	RMSE
^{13}C										
Form I	13[c]	A	**1.00**[d]	**1.02**	**–2.05**	**1.80**[e]	1.00	1.03	––3.69	2.25
	18	A	**1.00**	**1.02**	**–2.19**	**1.80**	1.00	1.03	–3.84	2.21
	03	A	**1.00**	**1.02**	**–2.10**	**1.80**	1.00	1.03	–3.75	2.21
	01	A	**1.00**	**1.02**	**–2.16**	**1.75**	1.00	1.03	–3.81	2.18
	13	B	0.99	1.01	–1.17	2.92	**1.00**	**1.03**	**–3.22**	**1.78**
	18	B	0.99	1.01	–1.29	2.92	**1.00**	**1.03**	**–3.34**	**1.78**
	03	B	0.99	1.01	–1.37	2.87	**1.00**	**1.03**	**–3.41**	**1.78**
	01	B	0.99	1.01	–1.34	2.86	**1.00**	**1.03**	**–3.37**	**1.80**
	17	A	**0.99**	**1.01**	**–1.32**	**2.47**	0.99	1.02	–3.01	2.59
	17	B	0.99	0.98	2.38	2.91	**0.99**	**0.99**	**0.51**	**2.28**
	02		0.99	1.01	–0.63	2.65	1.00	1.02	–2.61	1.66
Form II	14		0.97	1.00	0.54	5.21	0.98	1.02	–1.68	4.31
	15 (75%)[f]		0.98	0.99	1.86	4.87	0.98	1.01	–0.30	4.00
	15 (25%)		0.97	0.99	1.31	5.22	0.98	1.01	–0.93	4.28
Form III	16		0.99	1.02	–1.54	3.59	0.99	1.04	–3.47	3.15
^{1}H										
Form I	13	A	**0.97**	**1.15**	**–1.31**	**0.41**	0.91	1.08	–0.70	0.76
	18	A	**0.98**	**1.15**	**–1.30**	**0.40**	0.91	1.08	–0.70	0.75
	03	A	**0.98**	**1.16**	**–1.37**	**0.40**	0.91	1.09	–0.76	0.76
	01	A	**0.98**	**1.18**	**–1.49**	**0.41**	0.91	1.11	–0.87	0.78
	13	B	0.80	1.12	–1.05	1.23	**0.96**	**1.19**	**–1.50**	**0.53**
	18	B	0.81	1.12	–1.07	1.21	**0.97**	**1.19**	**–1.50**	**0.51**
	03	B	0.81	1.13	–1.13	1.21	**0.96**	**1.20**	**–1.55**	**0.52**
	01	B	0.81	1.13	–1.14	1.21	**0.96**	**1.20**	**–1.55**	**0.53**
	17	A	**0.92**	**0.69**	**1.61**	**0.44**	0.86	0.65	1.99	0.60
	17	B	0.66	0.71	1.54	1.11	**0.86**	**0.78**	**1.04**	**0.71**
	02		0.92	1.19	–1.59	0.75	0.96	1.18	–1.41	0.55
Form II	14		0.73	0.99	–0.36	1.31	0.91	1.07	–0.90	0.74
	15 (75%)		0.74	0.96	–0.01	1.26	0.90	1.04	–0.46	0.76
	15 (25%)		0.74	1.00	–0.44	1.31	0.92	1.09	–0.97	0.74
Form III	16		0.89	1.11	–1.05	0.85	0.98	1.13	–1.12	0.34

[a]Form I has two molecules in the asymmetric unit, which can be readily distinguished by their torsion 1 values (A ≅ 68°, B ≅ –58°). Fit parameters are given for $\Delta\delta_{Calculated}$ vs $\Delta\delta_{Experimental}$ data for either Molecule A or Molecule B treated separately.
[b]Values are for the fit parameters.
[c]CSD FURSEM entry ID (refer to Table 1).
[d]Values in **bold** indicate the fit parameters for the form corresponding to the measured experimental data, *i.e.,* the ones the approach should identify.
[e]RMSE values between $\delta_{Solid\ expt}$ and $\delta_{Solid\ calc}$ as calculated according to reference.[13] Note that in this approach, no ^{1}H or ^{13}C data points are removed due to exchange with solvent or other reasons. Values underlined indicate RMSE values that are within the thresholds for identifying a correct match (^{13}C 1.9 ppm, ^{1}H 0.33 ppm).[13]
[f]FURSEM15 has disorder around the furan ring, occupying two sites at 75% and 25% occupancy, respectively.

Table S21. Comparison of approaches for identifying the correct form from solid-state NMR chemical shift data *after allowing the unit cell to vary during DFT-D geometry optimisation.*

			Form I			
			Molecule A[a]		Molecule B[a]	
Form	CSD entry ID	Mol	$\Delta\delta$ regression[b] (p-value)	RMSE[c] (ppm)	$\Delta\delta$ regression (p-value)	RMSE (ppm)
^{13}C						
Form I	13[d]	A	**0.0106**[e, f]	**1.80**[e, g]	0.0829	2.25
	18	A	**0.0109**	**1.80**	0.0744	2.21
	03	A	**0.0109**	**1.80**	0.0717	2.21
	01	A	**0.0091**	**1.75**	0.0592	2.18
	13	B	0.3388	2.92	**0.0056**	**1.78**
	18	B	0.3407	2.92	**0.0053**	**1.78**
	03	B	0.3157	2.87	**0.0059**	**1.78**
	01	B	0.2977	2.86	**0.0054**	**1.80**
	17	A	**0.0439**	**2.47**	0.0738	2.59
	17	B	0.4822	2.91	**0.1678**	**2.28**
	02		0.2190	2.65	0.0016	1.66
Form II	14		0.7317	5.21	0.0967	4.31
	15 (75%)[h]		0.7429	4.87	0.1202	4.00
	15 (25%)		0.7308	5.22	0.0838	4.28
Form III	16		0.4016	3.59	0.1466	3.15
^{1}H						
Form I	13[d]	A	**0.0064**[e, f]	**0.41**[e,g]	0.9163	0.76
	18	A	**0.0055**	**0.40**	0.9175	0.75
	03	A	**0.0042**	**0.40**	0.9104	0.76
	01	A	**0.0046**	**0.41**	0.8899	0.78
	13	B	0.9883	1.23	**0.0386**	**0.53**
	18	B	0.9859	1.21	**0.0342**	**0.51**
	03	B	0.9836	1.21	**0.0340**	**0.52**
	01	B	0.9769	1.21	**0.0314**	**0.53**
	17	A	**0.0215**	**0.44**	0.7094	0.60
	17	B	0.9794	1.11	**0.0856**	**0.71**
	02		0.2433	0.75	0.4097	0.55
Form II	14		0.9654	1.31	0.1706	0.74
	15 (75%)[h]		0.9346	1.26	0.2668	0.76
	15 (25%)		0.9611	1.31	0.1492	0.74
Form III	16		0.9675	0.85	0.1010	0.34

[a]Form I has two molecules in the asymmetric unit, which can be readily distinguished by their torsion 1 values (A ≅ 68°, B ≅ –58°). Fit parameters are given for $\Delta\delta_{\text{Calculated}}$ vs $\Delta\delta_{\text{Experimental}}$ data for either Molecule A or Molecule B, treated separately.

[b]*i.e.,* the approach in this work.

[c]*i.e.,* the RMSE approach.[13]

[d]CSD FURSEM entry ID (refer to Table 1).

[e]Values in **bold** indicate the fit parameters for the form corresponding to the measured experimental data, *i.e.,* the ones the approaches should identify.

[f]p-values are for the null hypothesis that $m = 0$, and the alternative hypothesis $m > 0$. Values underlined reject the null hypothesis at a one-tailed significance level of 0.050, suggesting a significant positive correlation between $\Delta\delta_{\text{Calculated}}$ and $\Delta\delta_{\text{Experimental}}$. Data as in Table S18.

[g]RMSE values between $\delta_{\text{Solid expt}}$ and $\delta_{\text{Solid calc}}$ as calculated according to reference.[13] Note that in this approach, no ^{1}H or ^{13}C data points are removed due to exchange with solvent or other reasons. Values underlined indicate RMSE values that are within the thresholds for identifying a correct match (^{13}C 1.90 ppm, ^{1}H 0.33 ppm).[13] Data as in Table S20, with its associated linear regression parameters.

[h]FURSEM15 has disorder around the furan ring, occupying two sites at 75% and 25% occupancy, respectively.

3.8 Analysis 3: Choice of solvent and charge-state for solution data

Table S22. Solution-state NMR chemical shifts for furosemide ($\delta_{Solution\ expt}$) under different solvent conditions.

	$\delta_{Solution\ expt}$ (ppm *vs* internal d_6-DSS)			$\delta_{Solution\ expt}$ (ppm *vs* absolute TMS in $CDCl_3$)			Change in absolute solution chemical shift with change in solvent conditions (ppm)	
Nucleus[a]	Neutral		Charged	Neutral		Charged	*Solvent*	*Charge and solvent*
	DMSO–d_6[b]	80% D_2O 20 % DMSO–d_6 pH 2.11[b]	80% D_2O 20 % DMSOd6 pH 6.77[b]	DMSO–d_6[c]	80% D_2O 20 % DMSO–d_6 pH 2.11[c]	80% D_2O 20 % DMSO–d_6 pH 6.77[c]	From DMSO-d_6 to 80% D_2O, 20 % DMSO–d_6 pH 2.11	From DMSO-d_6 to 80% D_2O, 20 % DMSO–d_6 pH 6.77
C1	145.913[d]	145.585	145.418	143.339	142.828	142.661	–0.51	–0.68
C2	113.709	113.590	113.529	111.135	110.833	110.772	–0.30	–0.36
C3	110.797	110.752	110.419	108.223	107.995	107.662	–0.23	–0.56
C4	154.490	153.876	154.426	151.916	151.119	151.669	–0.80	–0.25
C5	42.279	41.979	41.945	39.705	39.222	39.188	–0.48	–0.52
C6	155.527	155.998	155.559	152.953	153.241	152.802	0.29	–0.15
C7	116.731	117.074	116.096	114.157	114.317	113.339	0.16	–0.82
C8	139.323	139.741	137.102	136.749	136.984	134.345	0.24	–2.40
C9	129.934	127.267	126.644	127.360	124.510	123.887	–2.85	–3.47
C10	136.468	136.870	136.223	133.894	134.113	133.466	0.22	–0.43
C11	111.307	111.076	119.155	108.733	108.319	116.398	–0.41	7.67
C12	171.775	172.158	175.574	169.201	169.401	172.817	0.20	3.62
H1	7.650	7.503	7.499	7.696	7.457	7.453	–0.24	–0.24
H2	6.449	6.446	6.439	6.495	6.400	6.393	–0.09	–0.10
H3	6.396	6.423	6.403	6.442	6.377	6.357	–0.06	–0.08
H4*[e]	4.611	4.566	4.498	4.657	4.520	4.452	–0.14	–0.20
H6	8.661	n/a[f]	n/a[f]	8.707	-	-		
H7	7.085	7.183	7.038	7.131	7.137	6.992	0.01	–0.14
H8	8.421	8.489	8.375	8.467	8.443	8.329	–0.02	–0.14
H9*[g]	7.360	n/a[f]	n/a[f]	7.406	-	-	-	-
H11	13.310	n/a[f]	n/a[f]	13.356	-	-	-	-

[a]Refer to Figure 1 for atom definitions.
[b]Chemical shifts of furosemide measured at 25 ºC at 10.0 (DMSO-d_6) or 2.5 mM (80% D_2O, 20 % DMSO–d_6), referenced directly (^1H) and indirectly (^{13}C) relative to internal d_6-DSS. pH value reported is that observed in the solvent system, rather than corrected to absolute.
[c]Details of how chemical shifts measured in different solvents and referenced relative to internal d_6-DSS have been re-referenced relative to absolute (*i.e.,* TMS in $CDCl_3$) are given in section 2.3. This re-refencing to the absolute scale is necessary to allow precise comparison of chemical shift perturbations caused by the differing conditions.
[d]Measurement errors are in ppm. In solution: $^1H \pm 0.001$, $^{13}C \pm 0.020$.
[e]H4 and H5 have identical chemical shifts in solution due to the absence of a chiral centre in the molecule, manifesting as a single resonance, labelled H4*.
[f]Value not measurable due to exchange with solvent.
[g]The sulphonamide hydrogens (H9, H10) are in rapid exchange in solution and manifest in spectra as a single broadened triplet resonance, labelled H9*.

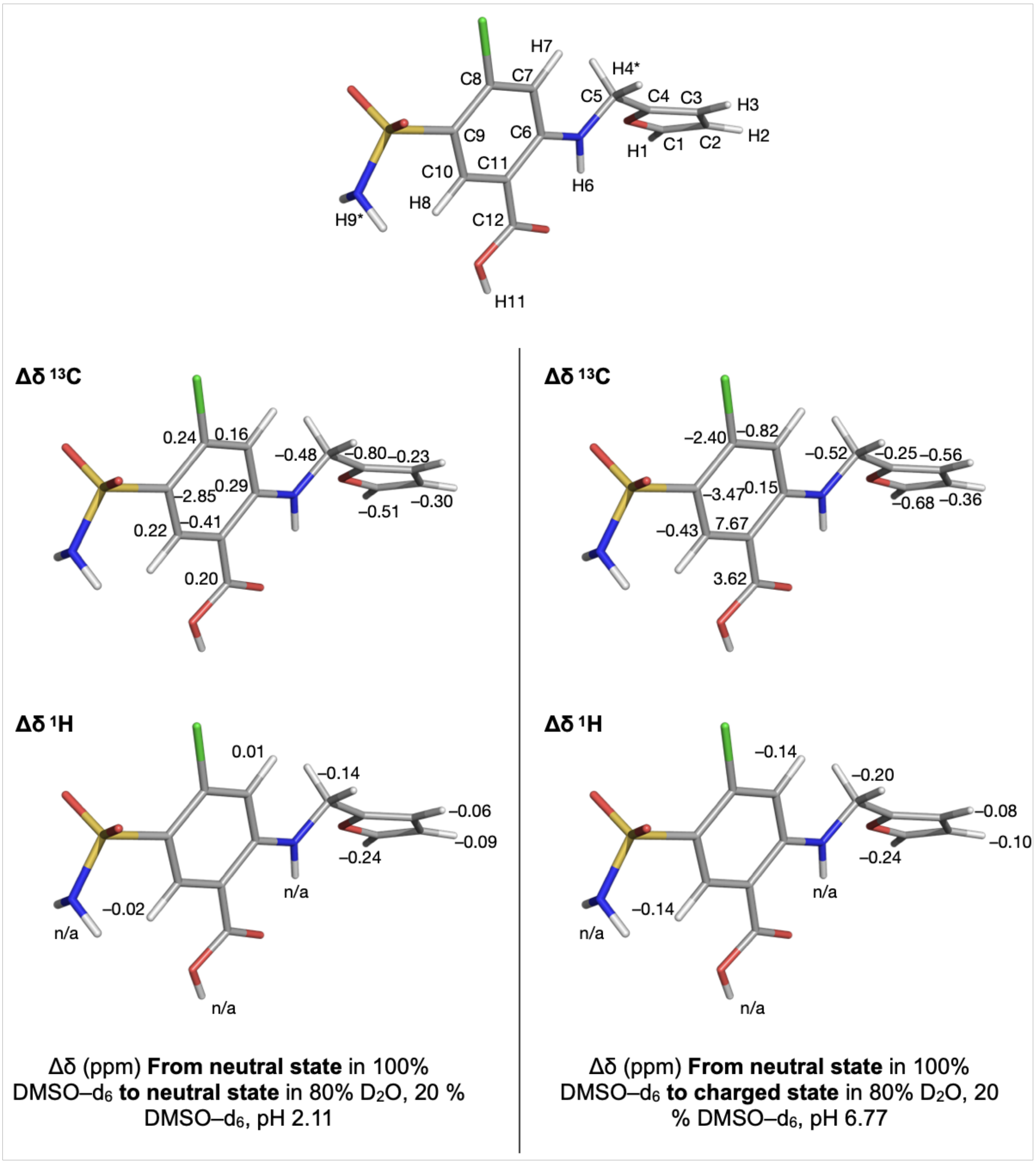

Figure S15. Change in absolute solution-state NMR chemical shift with change in solvent conditions. Data taken from Table S22.

Table S23. Comparison of the experimentally measured ($\Delta\delta_{Experimental}$) and DFT GIPAW calculated ($\Delta\delta_{Calcualted}$) differences in NMR chemical shifts for furosemide between the solution state and solid-state forms, *using solution chemical shifts measured in an aqueous environment in the neutral state (80% D_2O, 20% DMSO-d6, at pH 2.11).*

Nucleus	Experimentally measured change $\Delta\delta_{Experimental}$[a]		Calculated change $\Delta\delta_{Calculated}$[b]														
	Form I, Molecule		Form I, Molecule[c]											Form II			Form III
	A	B	13 A[d]	18 A	03 A	01 A	13 B	18 B	03 B	01 B	17 A	17 B	02	14	15 (75%)[e]	15 (25%)	16
C1	–4.05[f]	–1.36	–0.18	–0.09	–0.09	0.01	4.00	3.55	3.12	3.24	1.05	1.96	1.99	6.66	4.13	6.18	2.79
C2	–1.50	–3.06	4.52	4.51	4.07	3.85	1.37	1.53	1.39	1.79	4.28	1.93	0.13	4.65	6.52	5.27	4.66
C3	–1.38	–0.22	3.65	3.68	3.53	3.39	6.72	6.32	5.94	6.09	5.61	6.28	6.61	9.06	5.59	7.74	3.41
C4	1.32	–3.28	2.53	2.63	2.58	2.56	–0.33	–0.76	–0.76	–0.55	0.32	–1.28	–2.01	–5.52	–5.78	–5.30	3.51
C5	–2.38	–2.38	0.43	0.65	0.54	0.48	0.40	0.43	0.18	0.32	1.35	–0.02	1.46	2.66	2.82	2.20	–3.17
C6	–0.80	–2.00	1.76	1.88	1.87	1.92	0.84	1.01	0.86	0.92	1.49	1.09	1.51	–1.45	–1.09	–1.23	1.25
C7	–0.27	0.03	3.99	4.39	4.23	4.42	5.68	5.35	4.87	4.67	3.68	4.46	4.06	–0.71	–0.73	–0.31	–0.09
C8	–1.74	–1.74	–0.54	–1.13	–1.08	–1.02	–1.63	–1.79	–1.65	–1.47	1.32	–2.48	–0.49	3.34	2.94	3.31	–0.32
C9	–2.07	–0.17	–6.02	–5.90	–5.73	–5.54	–3.32	–3.40	–3.52	–3.59	–6.14	–2.96	–4.04	–1.40	–1.40	–0.81	–1.52
C10	–0.27	–1.51	3.27	3.32	3.00	3.05	1.57	1.62	1.41	1.36	1.98	–1.70	1.60	0.76	1.26	0.82	1.70
C11	–5.02	–2.47	1.77	1.75	1.63	1.42	4.63	4.51	4.10	3.52	1.62	3.96	2.52	1.47	2.31	1.33	3.33
C12	0.09	0.09	3.77	4.01	4.27	4.56	3.97	4.28	4.44	4.65	3.29	0.25	5.01	5.88	6.02	6.18	7.03
H1	–1.00	0.20	–0.70	–0.71	–0.74	–0.73	0.80	0.73	0.64	0.62	–0.71	0.19	0.07	0.70	0.74	0.79	0.08
H2	–0.05	–0.45	0.59	0.59	0.44	0.44	0.10	0.07	0.00	0.01	0.59	0.11	–0.78	0.76	1.48	0.75	0.37
H3	–0.72	–0.42	–0.59	–0.61	–0.61	–0.69	0.25	0.18	0.10	0.11	–0.48	0.04	–0.59	–0.09	–0.02	–0.02	0.41
H4*[g]	0.13	–0.27	0.22	0.25	0.24	0.32	–0.04	–0.04	–0.03	0.09	0.63	–0.16	–0.01	–0.08	–0.37	–0.14	–0.49
H6	n/a[h]	n/a[h]	–0.91	–0.94	–0.87	–0.82	–0.54	–0.52	–0.48	–0.36	–1.23	0.53	–0.22	–0.90	–0.73	–0.89	–0.47
H7	0.72	–0.98	0.90	0.87	0.83	0.83	–1.39	–1.41	–1.36	–1.35	0.26	–1.40	0.43	–1.94	–1.48	–1.87	–0.72
H8	0.21	0.11	0.07	0.07	0.05	0.13	–0.01	–0.01	–0.04	0.00	0.06	–0.31	0.11	–0.74	–0.67	–0.67	0.01
H9*[i]	n/a[h]	n/a[h]	2.39	2.48	2.51	2.42	3.04	3.07	3.08	3.08	1.48	1.41	–0.02	2.97	2.74	2.73	2.24
H11	n/a[h]	n/a[h]	8.46	8.65	8.68	9.34	8.65	8.94	8.90	9.50	3.98	5.38	9.86	8.05	8.44	7.82	8.49

[a]Difference between experimentally measured solid-state and solution-state NMR chemical shifts as per equation 1 ($\Delta\delta_{Experimental} = \delta_{Solid\ exp} - \delta_{Solution\ expt}$). Here $\delta_{Solution\ expt}$ is replaced compared to Table 2 with values from Table S22 for furosemide in an aqueous environment in the neutral state (*i.e.*, values in 80% D_2O, 20% DMSO-d_6, pH 2.11).
[b]Difference between GIPAW-calculated solid-state and solution-state NMR chemical shifts as per equation 2 ($\Delta\delta_{Calculated} = \delta_{Solid\ calc} - \delta_{Solution\ calc}$).
[c]Form I has two molecules in the asymmetric unit, which can be readily distinguished by their torsion 1 values (A ≅ 68°, B ≅ –58°).
[d]CSD FURSEM entry ID (refer to Table 1).
[e]FURSEM15 has disorder around the furan ring, occupying two sites at 75% and 25% occupancy, respectively.
[f]Errors are ^{1}H ± 0.2 ppm, ^{13}C ± 0.1 ppm.[15, 16]
[g]H4 and H5 have identical chemical shifts in solution due to the absence of a chiral centre in the molecule, manifesting as a single resonance, labelled H4*. The solid-state NMR chemical shifts are given as the mean of H4 and H5.
[h]No value measurable because these hydrogens had exchanged with D_2O.
[i]The sulphonamide hydrogens (H9, H10) are in rapid exchange in solution and manifest in spectra as a single broadened triplet resonance, labelled H9*. The solid-state NMR chemical shifts are given as the mean of H9 and H10.

Table S24. Linear regression analysis parameters and *p*-values for chemical shift differences between the solution state and the solid state for combinations of calculated (Forms I, II, III) and experimentally measured (Form I, Molecule A and Molecule B) differences in furosemide chemical shift *using solution chemical shifts measured in an aqueous environment in the neutral state (80% D_2O, 20% DMSO-d_6, at pH 2.11).*

$\Delta\delta_{Calculated}$ for			$\Delta\delta_{Experimental}$ for Form I							
			Molecule A[a]				Molecule B[a]			
Form	CSD entry ID	Mol	$r^{2\,b}$	m^b	c^b	*p*-value	$r^{2\,b}$	m^b	c^b	*p*-value
^{13}C										
Form I	13[c]	A	**0.14[d]**	**0.61**	**2.68**	**0.1274[e]**	0.03	–0.39	1.20	0.6850
	18	A	**0.15**	**0.63**	**2.83**	**0.1175**	0.02	–0.34	1.39	0.6626
	03	A	**0.16**	**0.64**	**2.76**	**0.1087**	0.01	–0.26	1.42	0.6313
	01	A	**0.19**	**0.67**	**2.82**	**0.0932**	0.01	–0.17	1.57	0.5873
	13	B	0.03	–0.29	1.90	0.6993	**0.11**	**0.79**	**3.49**	**0.1569**
	18	B	0.03	–0.27	1.83	0.6912	**0.11**	**0.78**	**3.38**	**0.1542**
	03	B	0.02	–0.20	1.70	0.6532	**0.12**	**0.76**	**3.14**	**0.1513**
	01	B	0.01	–0.14	1.84	0.6044	**0.11**	**0.74**	**3.13**	**0.1578**
	17	A	**0.02**	**0.25**	**2.05**	**0.3297**	0.00	–0.07	1.58	0.5329
	17	B	0.09	–0.46	0.58	0.8183	**0.03**	**0.40**	**1.86**	**0.3019**
	02		0.00	–0.07	1.61	0.5495	0.18	1.02	3.23	0.0963
Form II	14		0.15	–0.89	0.69	0.8769	0.11	1.14	3.70	0.1562
	15 (75%)[f]		0.15	–0.79	0.61	0.8784	0.04	0.63	2.71	0.2700
	15 (25%)		0.13	–0.79	0.84	0.8654	0.12	1.09	3.62	0.1487
Form III	16		0.03	0.26	2.47	0.3129	0.00	–0.07	1.97	0.5374
^{1}H										
Form I	13	A	**0.84**	**0.91**	**0.19**	**0.0051**	0.48	–1.02	–0.22	0.9355
	18	A	**0.84**	**0.91**	**0.18**	**0.0050**	0.46	–1.00	–0.23	0.9298
	03	A	**0.89**	**0.90**	**0.14**	**0.0025**	0.47	–0.97	–0.26	0.9343
	01	A	**0.91**	**0.95**	**0.16**	**0.0018**	0.39	–0.92	–0.23	0.9078
	13	B	0.77	–1.00	–0.17	0.9893	**0.69**	**1.40**	**0.38**	**0.0203**
	18	B	0.74	–0.96	–0.19	0.9858	**0.72**	**1.40**	**0.34**	**0.0168**
	03	B	0.72	–0.88	–0.22	0.9832	**0.74**	**1.32**	**0.29**	**0.0142**
	01	B	0.67	–0.85	–0.19	0.9771	**0.74**	**1.32**	**0.31**	**0.0144**
	17	A	**0.59**	**0.67**	**0.14**	**0.0362**	0.18	–0.55	–0.11	0.8017
	17	B	0.64	–0.74	–0.34	0.9714	**0.50**	**0.97**	**0.04**	**0.0587**
	02		0.20	0.35	–0.02	0.1858	0.00	–0.05	-0.08	0.5311
Form II	14		0.56	–1.18	–0.37	0.9563	0.34	1.37	0.18	0.1116
	15 (75%)		0.39	–1.03	–0.18	0.9078	0.15	0.95	0.23	0.2222
	15 (25%)		0.60	–1.21	–0.34	0.9647	0.36	1.39	0.23	0.1036
Form III	16		0.49	–0.50	–0.12	0.9382	0.18	0.45	0.08	0.2002

[a]Form I has two molecules in the asymmetric unit, which can be readily distinguished by their torsion 1 values (A ≅ 68°, B ≅ –58°). Fit parameters are given for $\Delta\delta_{Calculated}$ vs $\Delta\delta_{Experimental}$ data for either Molecule A or Molecule B treated separately. Here $\delta_{Solution\ expt}$ is replaced with values from Table S22 for furosemide in an aqueous environment in the neutral state (*i.e.*, values in 80% D_2O, 20% DMSO-d_6, pH 2.11).
[b]Values are for the fit parameters corresponding to the measured experimental data after omitting the chemical shifts for the ^{1}H atoms in exchange (H6, H9*, and H11) and the ^{13}C atom adjacent to the chlorine (C8).
[c]CSD FURSEM entry ID (refer to Table 1).
[d]Values in **bold** indicate the fit parameters for the form corresponding to the measured experimental data, *i.e.,* the ones the approach should identify.
[e]*p*-values are for the null hypothesis that $m = 0$, and the alternative hypothesis $m > 0$. Values underlined reject the null hypothesis at a one-tailed significance level of 0.050, suggesting a significant positive correlation between $\Delta\delta_{Calculated}$ and $\Delta\delta_{Experimenta}$. The lower bound of the one-sided 95% confidence intervals for the correlation between $\Delta\delta_{Calculated}$ and $\Delta\delta_{Experimental}$ are given in Table S25.
[f]FURSEM15 has disorder around the furan ring, occupying two sites at 75% and 25% occupancy, respectively.

Table S25. The lower bound of the one-sided 95% confidence intervals for the correlation of $\Delta\delta_{Calculated}$ vs $\Delta\delta_{Experimental}$ *using solution NMR chemical shifts measured in an aqueous environment in the neutral state (80% D_2O, 20% DMSO-d6, at pH 2.11) (see Table S24).*

Form	CSD entry ID	Mol[a]	**$\Delta\delta_{Experimental}$ *vs* $\Delta\delta_{Calculated}$ for Form I**	
			**Molecule A**	**Molecule B**
^{13}C				
Form I	13[b]	A	**–0.184**[c, d]	–0.633
	18	A	**–0.167**	–0.620
	03	A	**–0.151**	–0.602
	01	A	**–0.121**	–0.576
	13	B	–0.642	**–0.229**
	18	B	–0.637	**–0.225**
	03	B	–0.615	**–0.221**
	01	B	–0.587	**–0.230**
	17	A	**–0.406**	–0.544
	17	B	–0.714	**–0.383**
	02	-	–0.523	–0.091
Form II	14		–0.755	–0.228
	15 (75%)[e]		–0.756	–0.355
	15 (25%)		–0.746	–0.217
Form III	16		–0.392	–0.547
^{1}H				
Form I	13	A	**0.550**	–0.947
	18	A	**0.551**	–0.944
	03	A	**0.665**	–0.946
	01	A	**0.714**	–0.933
	13	B	–0.981	**0.236**
	18	B	–0.978	**0.285**
	03	B	–0.975	**0.327**
	01	B	–0.971	**0.325**
	17	A	**0.074**	–0.887
	17	B	–0.967	**–0.072**
	02	-	–0.435	–0.758
Form II	14		–0.958	–0.958
	15 (75%)[e]		–0.933	–0.933
	15 (25%)		–0.963	–0.963
Form III	16		–0.948	–0.948

[a]Form I has two molecules in the asymmetric unit, which can be readily distinguished by their torsion 1 values (A ≅ 68°, B ≅ –58°). Fit parameters are given for $\Delta\delta_{Calculated}$ vs $\Delta\delta_{Experimental}$ data for either Molecule A or Molecule B treated separately. Here $\delta_{Solution\ expt}$ is replaced with values from Table S22 for furosemide in an aqueous environment in the neutral state (*i.e.*, values in 80% D_2O, 20% DMSO-d_6, pH 2.11).
[b]CSD FURSEM entry ID (refer to Table 1).
[c]Values are for the fit parameters corresponding to the measured experimental data after omitting the chemical shifts for the ^{1}H atoms in exchange (H6, H9*, and H11) and the ^{13}C atom adjacent to the chlorine (C8).
[d]Values in **bold** indicate the fit parameters for the form corresponding to the measured experimental data, *i.e.,* the ones the approach should identify.
[e]FURSEM15 has disorder around the furan ring, occupying two sites at 75% and 25% occupancy, respectively.

Table S26. $\delta_{\text{Solution calc}}$ values for furosemide in neutral and charged states, calculated using an ensemble of 1,000 random conformations sampled from the solution dynamic 3D structure.

Nucleus[a]	$\delta_{\text{Solution calc}}$ (N = 1,000) (ppm)[b]		Calculated change from neutral to charged (ppm)
	Neutral[c]	Charged[d]	
C1	142.5 ± 0.05	139.2 ± 0.06	–3.3
C2	108.5 ± 0.03	106.6 ± 0.04	–1.9
C3	106.2 ± 0.11	102.9 ± 0.13	–3.3
C4	152.8 ± 0.09	159.3 ± 0.10	6.5
C5	36.1 ± 0.07	36.2 ± 0.07	0.1
C6	149.8 ± 0.05	152.4 ± 0.04	2.6
C7	111.9 ± 0.16	106.3 ± 0.16	–5.6
C8	143.0 ± 0.09	131.6 ± 0.09	–11.4
C9	131.5 ± 0.06	124.7 ± 0.05	–6.8
C10	133.0 ± 0.10	131.3 ± 0.08	–1.7
C11	103.5 ± 0.04	121.4 ± 0.04	17.9
C12	169.0 ± 0.02	166.3 ± 0.01	–2.7
H1	7.1 ± 0.01	7.1 ± 0.01	0.0
H2	6.0 ± 0.00	5.8 ± 0.00	–0.2
H3	5.8 ± 0.01	5.6 ± 0.01	–0.2
H4	3.9 ± 0.02	3.9 ± 0.02	0.0
H5	3.9 ± 0.02	3.9 ± 0.02	0.0
H6	8.8 ± 0.02	16.0 ± 0.02	7.2
H7	6.4 ± 0.06	6.0 ± 0.04	–0.4
H8	8.0 ± 0.00	7.9 ± 0.00	–0.1
H9	4.0 ± 0.05	3.9 ± 0.07	–0.1
H10	4.0 ± 0.04	3.7 ± 0.06	–0.3
H11	4.8 ± 0.00	n/a	n/a

[a]Refer to Figure 1 for atom definitions.
[b]Calculation of solution chemical shifts as described in sections 2.5 and 2.6.
[c]Values as in Table 2 and Table S5.
[d]Values calculated by removing the carboxylic acid hydrogen from the base conformation prior to DFT GIPAW geometry optimisation (CASTEP), and then repeating the workflow as described in sections 2.5 and 2.6.

Table S27. Comparison of the experimentally measured ($\Delta\delta_{Experimental}$) and DFT GIPAW calculated ($\Delta\delta_{Calcualted}$) differences in NMR chemical shifts for furosemide between the solution state and solid-state forms, *using solution chemical shifts measured in an aqueous environment in the charged state (80% D_2O, 20% DMSO-d6, at pH 6.77).*

Nucleus	Experimentally measured change $\Delta\delta_{Experimental}$[a]		Calculated change $\Delta\delta_{Calculated}$[b]														
	Form I[c]		Form I											Form II			Form III
	A	B	13 A[d]	18 A	03 A	01 A	13 B	18 B	03 B	01 B	17 A	17 B	02	14	15 (75%)[e]	15 (25%)	16
C1	–3.88[f]	–1.19	3.14	3.23	3.24	3.34	7.32	6.87	6.44	6.56	4.37	5.28	5.31	9.99	7.45	9.51	6.11
C2	–1.44	–3.00	6.35	6.34	5.90	5.68	3.20	3.36	3.22	3.62	6.11	3.76	1.96	6.48	8.35	7.10	6.49
C3	–1.05	0.11	6.95	6.97	6.83	6.69	10.02	9.62	9.24	9.39	8.91	9.58	9.91	12.36	8.89	11.04	6.71
C4	0.77	–3.83	–3.95	–3.84	–3.89	–3.91	–6.81	–7.24	–7.24	–7.03	–6.16	–7.76	–8.49	–12.00	–12.25	–11.78	–2.97
C5	–2.34	–2.34	0.40	0.62	0.51	0.45	0.37	0.40	0.15	0.29	1.32	–0.05	1.43	2.63	2.79	2.17	–3.20
C6	–0.36	–1.56	-0.81	–0.69	–0.70	–0.65	v1.73	–1.56	–1.71	–1.65	–1.08	–1.48	–1.06	–4.02	–3.66	–3.80	–1.32
C7	0.70	1.00	9.67	10.06	9.91	10.09	11.35	11.02	10.54	10.34	9.36	10.14	9.73	4.97	4.94	5.36	5.58
C8	0.90	0.90	10.82	10.23	10.28	10.34	9.73	9.56	9.71	9.88	12.67	8.87	10.87	14.69	14.30	14.67	11.04
C9	–1.44	0.46	0.80	0.91	1.09	1.28	3.50	3.41	3.29	3.23	0.67	3.85	2.78	5.41	5.42	6.00	5.29
C10	0.38	–0.86	4.97	5.02	4.70	4.74	3.27	3.32	3.11	3.06	3.67	0.00	3.30	2.46	2.96	2.51	3.39
C11	–13.10	–10.55	–16.10	–16.12	–16.23	–16.45	–13.24	–13.36	–13.77	–14.35	–16.25	–13.90	–15.35	–16.40	–15.55	–16.54	–14.54
C12	–3.32	–3.32	6.49	6.73	6.98	7.27	6.69	7.00	7.16	7.37	6.01	2.97	7.73	8.59	8.74	8.90	9.75
H1	–1.00	0.20	–0.62	–0.63	–0.66	–0.66	0.87	0.81	0.72	0.70	–0.63	0.27	0.15	0.77	0.81	0.86	0.16
H2	–0.04	–0.44	0.73	0.73	0.58	0.58	0.24	0.21	0.13	0.15	0.72	0.25	–0.64	0.90	1.62	0.89	0.50
H3	–0.70	–0.40	–0.43	–0.45	–0.44	–0.53	0.42	0.34	0.26	0.27	–0.32	0.20	–0.43	0.07	0.14	0.14	0.57
H4*[g]	0.20	–0.20	0.27	0.30	0.30	0.37	0.02	0.02	0.03	0.14	0.68	–0.11	0.00	–0.03	–0.32	–0.08	v0.44
H6	n/a[h]	n/a[h]	–8.06	–8.09	–8.02	–7.97	–7.69	–7.67	–7.63	–7.51	–8.38	–6.62	–7.37	–8.05	–7.88	–8.04	–7.62
H7	0.86	–0.84	1.31	1.28	1.24	1.24	–0.98	–0.99	–0.94	–0.94	0.67	–0.98	0.85	–1.52	–1.07	–1.46	–0.31
H8	0.32	0.22	0.14	0.14	0.12	0.20	0.06	0.06	0.03	0.07	0.13	–0.24	0.18	–0.67	–0.60	–0.60	0.08
H9*[i]	n/a[h]	n/a[h]	2.57	2.66	2.68	2.59	3.22	3.25	3.25	3.26	1.66	1.59	–0.08	3.14	2.91	2.90	2.42
H11	n/a[h]	n/a[h]	n/a[j]	n/a[j]	n/a[j]	n/a[j]	n/a[j]	n/a[j]	n/a[j]	n/a[j]	n/a[j]	n/a[j]	n/a[j]	n/a[j]	n/a[j]	n/a[j]	n/a[j]

[a]Difference between experimentally measured solid-state and solution-state NMR chemical shifts as per equation 1 ($\Delta\delta_{Experimental} = \delta_{Solid\ exp} - \delta_{Solution\ expt}$). Here $\delta_{Solution\ expt}$ is replaced compared to Table 2 with values from Table S22 for furosemide in an aqueous environment in the charged state (*i.e.*, values in 80% D_2O, 20% DMSO-d_6, pH 6.77).

[b]Difference between GIPAW-calculated solid-state and solution-state NMR chemical shifts as per equation 2 ($\Delta\delta_{Calculated} = \delta_{Solid\ calc} - \delta_{Solution\ calc}$). Here $\delta_{Solution\ calc}$ is replaced compared to Table 2 with values from Table S26 for furosemide in the charged state.

[c]Form I has two molecules in the asymmetric unit, which can be readily distinguished by their torsion 1 values (A ≅ 68°, B ≅ –58°).

[d]CSD FURSEM entry ID (refer to Table 1).

[e]FURSEM15 has disorder around the furan ring, occupying two sites at 75% and 25% occupancy, respectively.

[f]Errors are ^{1}H ± 0.2 ppm, ^{13}C ± 0.1 ppm.[15, 16]

[g]H4 and H5 have identical chemical shifts in solution due to the absence of a chiral centre in the molecule, manifesting as a single resonance, labelled H4*. The solid-state NMR chemical shifts are given as the mean of H4 and H5.

[h]No value measurable because these hydrogens had exchanged with D_2O.

[i]The sulphonamide hydrogens (H9, H10) are in rapid exchange in solution and manifest in spectra as a single broadened triplet resonance, labelled H9*. The solid-state NMR chemical shifts are given as the mean of H9 and H10.

[j]Atom omitted to create a charge state, see Figure 1.

Table S28. Linear regression analysis parameters and *p*-values for chemical shift differences between the solution state and the solid state for combinations of calculated (Forms I, II, III) and experimentally measured (Form I, Molecule A and Molecule B) differences in furosemide NMR chemical shift *using solution chemical shifts measured in an aqueous environment in the charged state (80% D_2O, 20% DMSO-d_6, at pH 6.77).*

$\Delta\delta_{Calculated}$ for			$\Delta\delta_{Experimental}$ for Form I							
			Molecule A[a]				Molecule B[a]			
Form	CSD entry ID	Mol	r^2 [b]	*m*[b]	c[b]	*p*-value	r^2 [b]	*m*[b]	c[b]	*p*-value
^{13}C										
Form I	13[c]	A	**0.55**[d]	**1.35**	**4.70**	**0.0047**[e]	0.70	1.88	5.92	0.0007
	18	A	**0.55**	**1.36**	**4.84**	**0.0046**	0.70	1.90	6.08	0.0006
	03	A	**0.55**	**1.35**	**4.75**	**0.0047**	0.71	1.90	6.00	0.0006
	01	A	**0.55**	**1.37**	**4.80**	**0.0047**	0.71	1.93	6.08	0.0006
	13	B	0.32	1.05	4.58	0.0354	**0.70**	**1.93**	**6.57**	**0.0007**
	18	B	0.32	1.05	4.47	0.0354	**0.69**	**1.91**	**6.44**	**0.0008**
	03	B	0.33	1.06	4.28	0.0329	**0.69**	**1.91**	**6.21**	**0.0007**
	01	B	0.34	1.11	4.41	0.0292	**0.70**	**1.95**	**6.33**	**0.0007**
	17	A	**0.46**	**1.28**	**4.47**	**0.0114**	0.70	1.96	6.01	0.0007
	17	B	0.34	1.05	3.53	0.0305	**0.74**	**1.93**	**5.52**	**0.0004**
	02		0.34	1.15	4.18	0.0297	0.70	2.03	6.20	0.0006
Form II	14		0.19	1.01	4.17	0.0905	0.55	2.12	6.70	0.0049
	15 (75%)[f]		0.20	0.97	3.85	0.0860	0.52	1.94	6.07	0.0062
	15 (25%)		0.20	1.04	4.23	0.0818	0.56	2.12	6.69	0.0042
Form III	16		0.39	1.11	4.48	0.0196	0.60	1.69	5.80	0.0027
^{1}H										
Form I	13	A	**0.80**	**0.94**	**0.29**	**0.0083**	0.51	–1.26	–0.07	0.9453
	18	A	**0.80**	**0.94**	**0.28**	**0.0078**	0.50	–1.24	–0.07	0.9418
	03	A	**0.84**	**0.92**	**0.24**	**0.0050**	0.51	–1.21	–0.10	0.9435
	01	A	**0.88**	**0.97**	**0.26**	**0.0030**	0.43	–1.14	–0.07	0.9211
	13	B	0.86	–0.83	0.06	0.9961	**0.49**	**1.05**	**0.36**	**0.0596**
	18	B	0.83	–0.79	0.03	0.9941	**0.53**	**1.06**	**0.33**	**0.0508**
	03	B	0.81	–0.72	0.00	0.9930	**0.56**	**0.99**	**0.28**	**0.0445**
	01	B	0.77	-0.69	0.03	0.9890	**0.57**	**1.00**	**0.31**	**0.0420**
	17	A	**0.69**	**0.70**	**0.25**	**0.0206**	0.33	–0.81	0.02	0.8814
	17	B	0.72	–0.59	-0.13	0.9842	**0.27**	**0.61**	**0.05**	**0.1472**
	02		0.32	0.45	0.11	0.1207	0.02	–0.20	0.04	0.6129
Form II	14		0.60	–1.03	–0.14	0.9649	0.16	0.90	0.14	0.2146
	15 (75%)[f]		0.39	–0.90	0.05	0.9084	0.03	0.44	0.20	0.3657
	15 (25%)		0.64	–1.05	–0.10	0.9724	0.18	0.92	0.18	0.2043
Form III	16		0.38	–0.37	0.07	0.9055	0.01	0.10	0.12	0.4254

[a]Form I has two molecules in the asymmetric unit, which can be readily distinguished by their torsion 1 values (A ≅ 68°, B ≅ –58°). Fit parameters are given for $\Delta\delta_{Calculated}$ vs $\Delta\delta_{Experimental}$ data for either Molecule A or Molecule B treated separately. Here $\delta_{Solution\ expt}$ is replaced with values from Table S22 for furosemide in an aqueous environment in the charged state (*i.e.*, values in 80% D_2O, 20% DMSO-d_6, pH 6.77).

[b]Values are for the fit parameters corresponding to the measured experimental data after omitting the chemical shifts for the ^{1}H atoms in exchange (H6, H9*, and H11) and the ^{13}C atom adjacent to the chlorine (C8).

[c]CSD FURSEM entry ID (refer to Table 1).

[d]Values in **bold** indicate the fit parameters for the form corresponding to the measured experimental data, *i.e.,* the ones the approach should identify.

[e]*p*-values are for the null hypothesis that *m* = 0, and the alternative hypothesis *m* > 0. Values underlined reject the null hypothesis at a one-tailed significance level of 0.050, suggesting a significant positive correlation between $\Delta\delta_{Calculated}$ and $\Delta\delta_{Experimental}$. The lower bound of the one-sided 95% confidence intervals for the correlation between $\Delta\delta_{Calculated}$ and $\Delta\delta_{Experimental}$ are given in Table S29.

[f]FURSEM15 has disorder around the furan ring, occupying two sites at 75% and 25% occupancy, respectively.

Table S29. The lower bound of the one-sided 95% confidence intervals for the correlation of $\Delta\delta_{Experimental}$ vs $\Delta\delta_{Calculated}$ *using solution NMR chemical shifts measured in an aqueous environment in the charged state (80% D_2O, 20% DMSO-d6, at pH 6.77) (see Table S28).*

Form	CSD entry ID	Mol[a]	$\Delta\delta_{Experimental}$ *vs* $\Delta\delta_{Calculated}$ for Form I	
			Molecule A	Molecule B
^{13}C				
Form I	13[b]	A	**0.351**[c, d]	0.557
	18	A	**0.353**	0.560
	03	A	**0.350**	0.567
	01	A	**0.351**	0.573
	13	B	0.057	**0.553**
	18	B	0.057	**0.545**
	03	B	0.069	**0.547**
	01	B	0.089	**0.553**
	17	A	**0.234**	0.552
	17	B	0.082	**0.610**
	02	-	0.086	0.559
Form II	14		-0.115	0.351
	15 (75%)[e]		-0.105	0.315
	15 (25%)		-0.095	0.366
Form III	16		0.153	0.418
^{1}H				
Form I	13	A	**0.451**	–0.952
	18	A	**0.464**	–0.950
	03	A	**0.552**	–0.951
	01	A	**0.639**	–0.940
	13	B	–0.989	**–0.076**
	18	B	–0.986	**–0.027**
	03	B	–0.985	**0.013**
	01	B	–0.980	**0.030**
	17	A	**0.232**	–0.921
	17	B	–0.976	**–0.361**
	02	-	–0.298	–0.801
Form II	14		–0.963	–0.480
	15 (75%)[e]		–0.934	–0.645
	15 (25%)		–0.968	–0.465
Form III	16		–0.932	–0.691

[a]Form I has two molecules in the asymmetric unit, which can be readily distinguished by their torsion 1 values (A ≅ 68°, B ≅ –58°). Fit parameters are given for $\Delta\delta_{Calculated}$ vs $\Delta\delta_{Experimental}$ data for either Molecule A or Molecule B treated separately. Here $\delta_{Solution\ expt}$ is replaced with values from Table S22 for furosemide in an aqueous environment in the charged state (*i.e.*, values in 80% D_2O, 20% DMSO-d_6, pH 6.77).
[b]CSD FURSEM entry ID (refer to Table 1).
[c]Values are for the fit parameters corresponding to the measured experimental data after omitting the chemical shifts for the ^{1}H atoms in exchange (H6, H9*, and H11) and the ^{13}C atom adjacent to the chlorine (C8).
[d]Values in **bold** indicate the fit parameters for the form corresponding to the measured experimental data, *i.e.,* the ones the approach should identify.
[e]FURSEM15 has disorder around the furan ring, occupying two sites at 75% and 25% occupancy, respectively.

3.9 Analysis 4: Approximation of the solution dynamic 3D structure using a substitute ensemble of furosemide conformations from the CSD

Table S30. Torsion angle values from all single-crystal diffraction structures in the CSD[a] containing neutral furosemide.

CSD entry ID	Torsion angle (°)					
	Tor 1[b]	Tor 2	Tor 3	Tor 4	Tor 5	Tor 6
BOKHAM	44.2	177.7	172.6	–69.3	–179.4	–90.3
BOKHAM01	–59.8	–71.3	169.5	65.1	178.0	–52.8
BOKHAM02	44.2	177.5	172.8	–69.5	–179.3	–90.4
BOKHEQ	60.3	84.8	159.5	–79.9	–177.1	–60.4
BOKHIU	62.5	74.7	176.4	55.2	173.9	–82.2
BOKHOA	–64.1	–168.4	179.9	–57.8	–177.6	–113.8
BOKHUG	75.1	178.1	–179.1	–55.0	–175.8	–88.8
BOKJAO	62.6	73.9	177.7	56.5	174.1	–83.7
ESAVIF	70.5	70.7	178.2	–63.3	–176.6	–59.7
ESAWAY	67.4	172.2	178.5	–63.0	179.4	–57.7
ESAWEC	–87.1	163.6	173.4	–64.7	–176.0	–67.9
ESAWIG	–86.7	166.9	172.9	–57.1	–178.2	–73.3
ESAWOM	87.1	–160.3	–174.8	64.2	177.1	–73.9
ESAWUS	–87.0	163.4	175.2	–63.8	–173.9	–62.7
EZIPIO	–55.3	–71.9	–178.6	–60.6	173.0	–51.9
FEFYAS	65.5	67.5	–178.2	–66.7	–179.0	–72.4
FEFYEW	69.2	75.3	–178.4	–62.8	–177.9	–57.5
FEFYIA	–93.4	89.4	176.1	68.9	169.3	–68.8
FURSEM01[c]	–53.9	–63.0	165.6	164.9	179.8	–6.9
FURSEM01	–67.2	83.5	–175.2	–166.3	–174.6	–166.6
FURSEM03	56.3	62.1	–164.2	–164.1	–179.5	–88.1
FURSEM03	67.6	–83.7	175.5	166.0	173.1	–65.4
FURSEM13	–57.6	–61.4	163.6	163.2	179.4	–56.5
FURSEM13	–68.2	84.0	–175.8	–166.0	–172.3	–76.6
FURSEM14	–78.2	–166.4	174.3	–79.9	175.2	–71.2
FURSEM16	–60.0	91.3	–168.8	55.7	176.5	–72.9
FURSEM17	–65.5	83.1	–174.8	–167.1	–174.4	–0.9
FURSEM17	52.0	64.4	–166.4	–166.3	–179.9	0.1
FURSEM18	–57.2	–62.0	163.9	164.0	179.1	–60.6
FURSEM18	68.0	–83.8	175.1	165.4	172.4	–70.5
HUQWAT	–62.5	–162.0	171.0	–76.3	178.2	–71.2
HUQWAT	–76.5	–163.1	172.3	175.6	–178.3	–113.9
IWERUY	–64.7	–173.3	176.7	–62.4	173.0	–65.4
LODFUH	59.8	–86.4	179.7	64.1	179.3	–83.4
LODGAO	–59.9	–169.0	–177.9	62.6	176.7	–77.5
LODGAO	55.2	179.7	175.9	–65.7	–179.1	–62.6
LODGAO01	–69.1	87.5	–172.9	–74.5	178.0	–70.9
LODGAO02	–56.3	–81.5	179.2	–62.8	177.0	–74.6
LODGAO02	–75.4	88.2	–176.7	60.8	–179.7	–64.1
LODGES	52.0	79.4	–172.0	61.4	178.9	–109.9
LOFLAV	–172.2	–85.1	–169.5	70.6	177.3	–81.8
LOFLID	62.8	175.7	–179.3	–66.0	175.2	–64.1
NOLBAU	–46.8	–72.2	172.5	–59.1	178.9	–103.6
NOLBEY	–67.0	–72.2	162.4	–61.2	–175.7	–54.1
NOLBIC	–54.6	–71.1	165.9	–61.2	179.7	–49.4
TARPIO	159.0	79.7	–177.7	–65.6	178.1	–59.3
TARPIO01	–84.2	86.8	–172.4	66.5	179.3	–72.1
TARPIO01	49.2	–86.2	172.4	–68.2	178.6	–54.8
TARPIO02	–72.3	–87.2	–177.2	–64.3	171.8	–138.3
TARPOU	–165.9	73.0	–174.1	–67.5	174.9	–54.9
VARGAZ	163.2	76.5	–170.6	–60.5	–174.3	–48.6
VARHII	161.0	–78.5	170.1	63.7	–178.5	–90.6
XIFRAH	–168.7	–76.3	179.2	–60.7	–179.0	–114.6
YASGOQ04	80.1	174.7	–168.6	74.4	172.4	–69.1

[a]As present in CSD version 5.42.
[b]Refer to Figure 1 for torsion defintions.
[c]Repeated entries denote structures with $Z' > 1$.

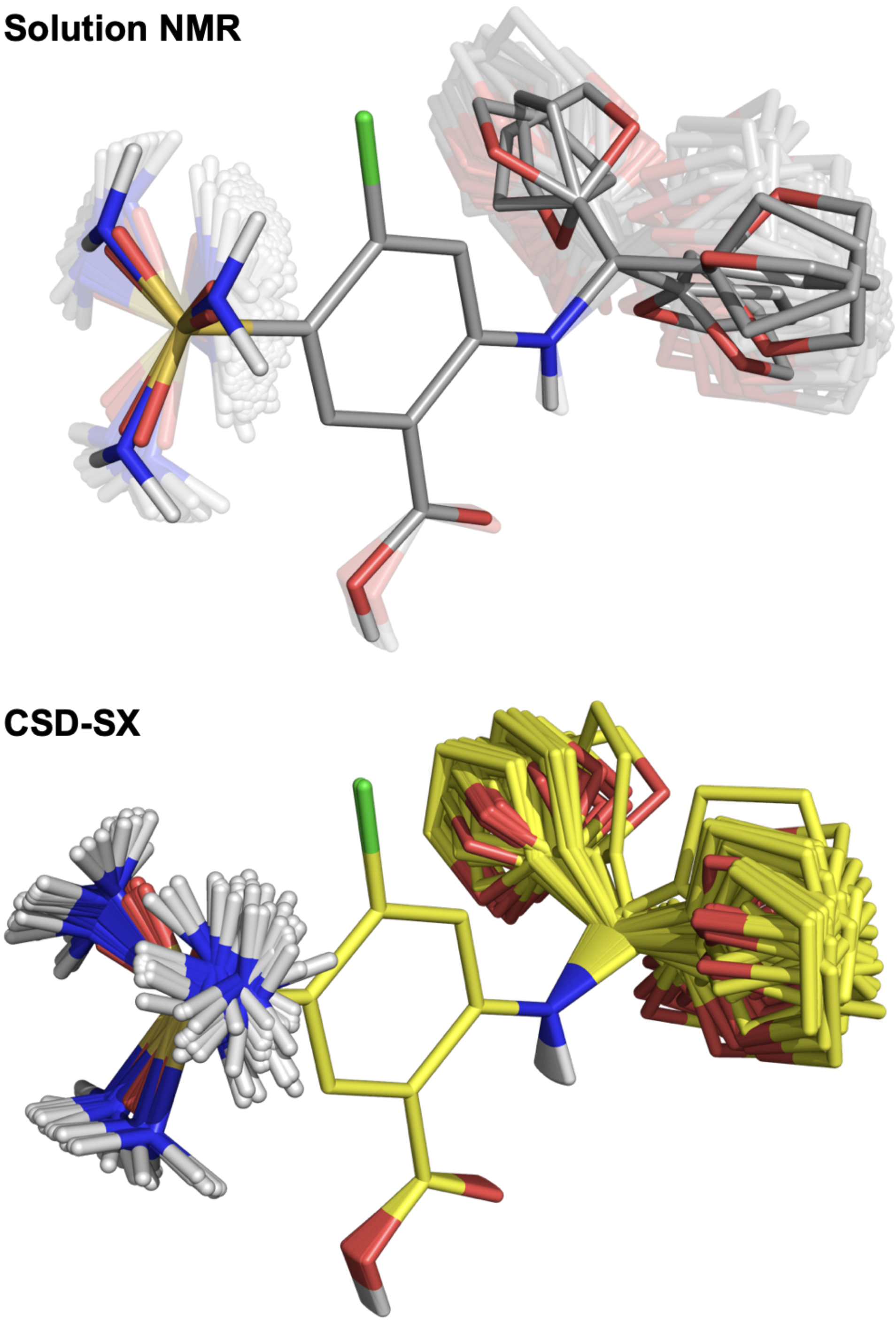

Figure S16. Comparison of furosemide solution dynamic 3D structure (grey carbons, in conformational ensemble representation) and the substitute ensemble (yellow carbons, 'CSD-SX') for estimating $\delta_{\text{Solution calc}}$ created from neutral furosemide conformations in single crystals structures in the CSD. The solution dynamic 3D structure is shown with all its conformers (bright) overlaid on an ensemble representative for the range of libration in solution (faded). The CSD-SX ensemble comprises 108 conformations from 45 single-crystal diffraction structures (see main text and Table S30). All conformations are overlaid on the central aromatic ring. Oxygen atoms are coloured red, nitrogen blue, chlorine green, hydrogen white and carbons in grey (solution). Non-polar hydrogens have been omitted for clarity.

Table S31. $\delta_{Solution\ calc}$ values for furosemide from the CSD-SX substitute ensemble (108 conformations) compared to the solution dynamic 3D structure.

Nucleus[a]	$\delta_{Solution\ calc}$ (ppm)[b]		Difference (ppm)
	Solution dynamic 3D structure (N = 1,000) (ppm)[c]	CSD-SX substitute (N = 108) (ppm)[d]	
C1	142.5 ± 0.05	143.0 ± 0.19	–0.5
C2	108.5 ± 0.03	108.5 ± 0.15	0.0
C3	106.2 ± 0.11	107.9 ± 0.47	–1.7
C4	152.8 ± 0.09	151.8 ± 0.45	1.0
C5	36.1 ± 0.07	36.5 ± 0.35	–0.4
C6	149.8 ± 0.05	149.8 ± 0.20	0.0
C7	111.9 ± 0.16	112.1 ± 0.41	–0.2
C8	143.0 ± 0.09	144.3 ± 0.48	–1.3
C9	131.5 ± 0.06	130.0 ± 0.51	1.5
C10	133.0 ± 0.10	133.4 ± 0.67	–0.4
C11	103.5 ± 0.04	103.7 ± 0.21	–0.2
C12	169.0 ± 0.02	168.9 ± 0.09	0.1
H1	7.1 ± 0.01	7.1 ± 0.03	0.0
H2	6.0 ± 0.00	6.0 ± 0.02	0.0
H3	5.8 ± 0.01	5.8 ± 0.05	0.0
H4	3.9 ± 0.02	3.8 ± 0.07	0.1
H5	3.9 ± 0.02	3.8 ± 0.06	0.1
H6	8.8 ± 0.02	8.6 ± 0.10	0.2
H7	6.4 ± 0.06	6.0 ± 0.09	0.4
H8	8.0 ± 0.00	8.0 ± 0.04	0.0
H9	4.0 ± 0.05	3.8 ± 0.15	0.2
H10	4.0 ± 0.04	3.8 ± 0.11	0.2
H11	4.8 ± 0.00	4.8 ± 0.01	0.0

[a]Refer to Figure 1 for atom definitions.
[b]Calculation of solution NMR chemical shifts as described in sections 2.5 and 2.6.
[c]Values as in Table 2 and Table S5.
[d]Values calculated from an ensemble comprising 108 conformations formed by applying extracted torsion values from neutral furosemide crystal structures to the base conformation prior to DFT GIPAW geometry optimisation (CASTEP), and then repeating the workflow as described in sections 2.5 and 2.6.

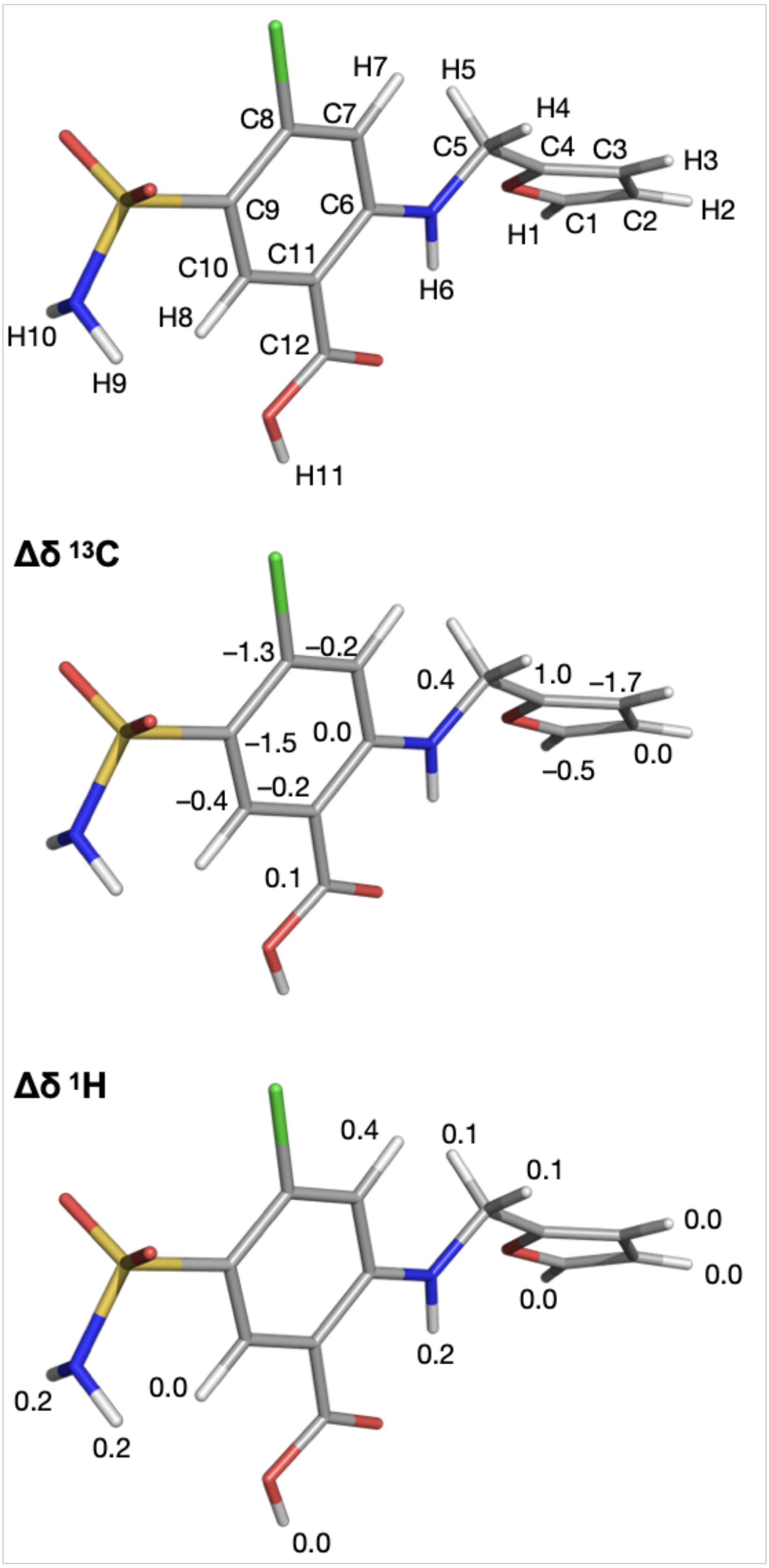

Figure S17. Change in calculated solution NMR chemical shift between solution dynamic 3D structure and the substitute ensemble created from neutral conformations of furosemide in single-crystal diffraction structures. Data taken from Table S31.

Table S32. Linear regression analysis parameters and p-values for chemical shift differences between the solution state and the solid state for combinations of calculated (Forms I, II, III) and experimentally measured (Form I, Molecule A and Molecule B) differences in furosemide chemical shift *using a substitute ensemble from CSD single-crystal diffraction structures to calculate* $\delta_{Solution\ calc}$.

$\Delta\delta_{Calculated}$ for			$\Delta\delta_{Experimental}$ for Form I							
			Molecule A[a]				Molecule A[a]			
Form	CSD entry ID	Mol	r^{2} [b]	*m*[b]	c[b]	*p*-value	r^{2} [b]	*m*[b]	c[b]	*p*-value
^{13}C										
Form I	13[c]	A	**0.44**[d]	**0.85**	**0.64**	**0.0096**[e]	0.05	0.43	1.02	0.2398
	18	A	**0.42**	**0.86**	**0.68**	**0.0110**	0.05	0.46	1.05	0.2321
	03	A	**0.44**	**0.85**	**0.62**	**0.0094**	0.07	0.49	0.95	0.2105
	01	A	**0.47**	**0.87**	**0.63**	**0.0072**	0.08	0.55	0.92	0.1807
	13	B	0.00	0.09	1.75	0.4159	**0.26**	**0.98**	**0.93**	**0.0456**
	18	B	0.01	0.11	1.63	0.3935	**0.28**	**0.99**	**0.81**	**0.0400**
	03	B	0.02	0.15	1.40	0.3478	**0.29**	**0.98**	**0.64**	**0.0346**
	01	B	0.03	0.20	1.40	0.2951	**0.30**	**0.97**	**0.69**	**0.0327**
	17	A	**0.29**	**0.61**	**0.93**	**0.0349**	0.17	0.69	0.86	0.0927
	17	B	0.01	–0.14	0.93	0.6414	**0.06**	**0.45**	**0.38**	**0.2232**
	02		0.09	0.33	1.37	0.1793	0.71	1.36	0.45	0.0005
Form II	14		0.07	–0.43	2.35	0.7900	0.16	1.00	1.03	0.0991
	15 (75%)[f]		0.06	–0.36	2.05	0.7701	0.08	0.65	1.12	0.1826
	15 (25%)		0.06	–0.38	2.31	0.7782	0.15	0.92	1.10	0.1035
Form III	16		0.06	0.34	1.41	0.2229	0.00	0.06	1.66	0.4626
^{1}H										
Form I	13	A	**0.83**	**0.96**	**0.30**	**0.0056**	0.47	–1.34	–0.32	0.9347
	18	A	**0.83**	**0.96**	**0.30**	**0.0057**	0.46	–1.32	–0.32	0.9306
	03	A	**0.86**	**0.95**	**0.26**	**0.0039**	0.46	–1.28	–0.35	0.9319
	01	A	**0.87**	**0.98**	**0.28**	**0.0031**	0.39	–1.22	–0.31	0.9085
	13	B	0.82	–0.77	–0.11	0.9933	**0.54**	**1.16**	**0.42**	**0.0472**
	18	B	0.78	–0.74	–0.14	0.9901	**0.58**	**1.17**	**0.39**	**0.0402**
	03	B	0.75	–0.67	–0.16	0.9873	**0.59**	**1.09**	**0.33**	**0.0371**
	01	B	0.70	–0.64	–0.13	0.9804	**0.59**	**1.09**	**0.36**	**0.0378**
	17	A	**0.67**	**0.73**	**0.24**	**0.0229**	0.27	–0.85	–0.17	0.8539
	17	B	0.65	–0.53	–0.28	0.9732	**0.34**	**0.71**	**0.06**	**0.1143**
	02		0.25	0.44	0.07	0.1535	0.04	–0.33	–0.12	0.6531
Form II	14		0.56	–0.96	–0.33	0.9573	0.17	0.99	0.17	0.2051
	15 (75%)[f]		0.36	–0.81	–0.12	0.8956	0.05	0.54	0.20	0.3416
	15 (25%)		0.61	–0.98	–0.29	0.9664	0.19	1.01	0.22	0.1930
Form III	16		0.36	–0.29	–0.04	0.8974	0.07	0.23	0.09	0.3073

[a]Form I has two molecules in the asymmetric unit, which can be readily distinguished by their torsion 1 values (A ≅ 68°, B ≅ –58°). Fit parameters are given for $\Delta\delta_{Calculated}$ vs $\Delta\delta_{Experimental}$ data for either Molecule A or Molecule B treated separately. Here $\delta_{Solution\ calc}$ is replaced with values from Table S31 for the ensemble of neutral furosemide conformations extracted from the CSD.

[b]Values are for the fit parameters corresponding to the measured experimental data after omitting the chemical shifts for the ^{1}H atoms in exchange (H6, H9*, and H11) and the ^{13}C atom adjacent to the chlorine (C8).

[c]CSD FURSEM entry ID (refer to Table 1).

[d]Values in **bold** indicate the fit parameters for the form corresponding to the measured experimental data, *i.e.,* the ones the approach should identify.

[e]p-values are for the null hypothesis that $m = 0$, and the alternative hypothesis $m > 0$. Values underlined reject the null hypothesis at a one-tailed significance level of 0.050, suggesting a significant positive correlation between $\Delta\delta_{Experimental}$ and $\Delta\delta_{Calculated}$. The lower bound of the one-sided 95% confidence intervals for the correlation between $\Delta\delta_{Experimental}$ and $\Delta\delta_{Calculated}$ are given in Table S33.

[f]FURSEM15 has disorder around the furan ring, occupying two sites at 75% and 25% occupancy, respectively.

Table S33. The lower bound of the one-sided 95% confidence intervals for the correlation of $\Delta\delta_{Calculated}$ vs $\Delta\delta_{Experimental}$ *using a substitute ensemble from CSD single crystal structures to calculate $\delta_{Solution\ calc}$ (see Table S32).*

Form	CSD entry ID	Mol[a]	$\Delta\delta_{Experimental}$ *vs* $\Delta\delta_{Calculated}$ for Form I	
			Molecule A	Molecule B
^{13}C				
Form I	13[b]	A	**0.351**[c, d]	–0.286
	18	A	**0.378**	–0.263
	03	A	**0.405**	–0.237
	01	A	**0.450**	–0.198
	13	B	–0.431	**0.223**
	18	B	–0.410	**0.276**
	03	B	–0.371	**0.293**
	01	B	–0.320	**0.304**
	17	A	**0.055**	–0.113
	17	B	–0.595	**–0.214**
	02	-	–0.258	0.575
Form II	14		–0.689	–0.156
	15 (75%)[e]		–0.676	–0.277
	15 (25%)		–0.682	–0.164
Form III	16		–0.283	–0.475
^{1}H				
Form I	13	A	**0.531**	–0.946
	18	A	**0.528**	–0.944
	03	A	**0.596**	–0.945
	01	A	**0.632**	–0.934
	13	B	–0.985	**–0.005**
	18	B	–0.982	**0.043**
	03	B	–0.979	**0.067**
	01	B	–0.973	**0.062**
	17	A	**0.203**	-0.909
	17	B	–0.968	**–0.281**
	02	-	–0.375	–0.821
Form II	14		–0.958	–0.466
	15 (75%)[e]		–0.928	–0.624
	15 (25%)		–0.964	–0.447
Form III	16		–0.929	–0.592

[a]Form I has two molecules in the asymmetric unit, which can be readily distinguished by their torsion 1 values (A ≅ 68°, B ≅ –58°). Fit parameters are given for $\Delta\delta_{Calculated}$ vs $\Delta\delta_{Experimental}$ data for either Molecule A or Molecule B treated separately. Here $\delta_{Solution\ calc}$ is replaced with values from Table S31 for the ensemble of neutral furosemide conformations extracted from the CSD.
[b]CSD FURSEM entry ID (refer to Table 1).
[c]Values are for the fit parameters corresponding to the measured experimental data after omitting the chemical shifts for the ^{1}H atoms in exchange (H6, H9*, and H11) and the ^{13}C atom adjacent to the chlorine (C8).
[d]Values in **bold** indicate the fit parameters for the form corresponding to the measured experimental data, *i.e.,* the ones the approach should identify.
[e]FURSEM15 has disorder around the furan ring, occupying two sites at 75% and 25% occupancy, respectively.

Supporting Information References

(1) Liu, M.; Mao, X.-a.; Ye, C.; Huang, H.; Nicholson, J. K.; Lindon, J. C. Improved WATERGATE Pulse Sequences for Solvent Suppression in NMR Spectroscopy. *J. Magn. Reson.* **1998**, *132* (1), 125-129. DOI: https://doi.org/10.1006/jmre.1998.1405.

(2) Adams, R. W.; Holroyd, C. M.; Aguilar, J. A.; Nilsson, M.; Morris, G. A. "Perfecting" WATERGATE: clean proton NMR spectra from aqueous solution. *Chem. Commun.* **2013**, *49* (4), 358-360. DOI: 10.1039/C2CC37579F.

(3) Palmer, A. G.; Cavanagh, J.; Wright, P. E.; Rance, M. Sensitivity improvement in proton-detected two-dimensional heteronuclear correlation NMR spectroscopy. *J. Magn. Reson.* **1991**, *93* (1), 151-170. DOI: https://doi.org/10.1016/0022-2364(91)90036-S.

(4) Kay, L. E.; Keifer, P.; Saarinen, T. Pure absorption gradient enhanced heteronuclear single quantum correlation spectroscopy with improved sensitivity. *J. Am. Chem. Soc.* **1992**, *114* (26), 10663-10665. DOI: 10.1021/ja00052a088.

(5) Schleucher, J.; Schwendinger, M.; Sattler, M.; Schmidt, P.; Schedletzky, O.; Glaser, S. J.; Sørensen, O. W.; Griesinger, C. A general enhancement scheme in heteronuclear multidimensional NMR employing pulsed field gradients. *J. Biomol. NMR* **1994**, *4* (2), 301-306. DOI: 10.1007/BF00175254.

(6) Kupce, Ē.; Freeman, R.; Wider, G.; Wüthrich, K. Suppression of Cycling Sidebands Using Bi-level Adiabatic Decoupling. *J. Magn. Reson., Ser. A* **1996**, *122* (1), 81-84. DOI: https://doi.org/10.1006/jmra.1996.0180.

(7) Claridge, T. D. W.; Pérez-Victoria, I. Enhanced ^{13}C resolution in semi-selective HMBC: a band-selective, constant-time HMBC for complex organic structure elucidation by NMR. *Org. Biomol. Chem.* **2003**, *1* (21), 3632-3634. DOI: 10.1039/B307122G.

(8) Furrer, J. A robust, sensitive, and versatile HMBC experiment for rapid structure elucidation by NMR: IMPACT-HMBC. *Chem. Commun.* **2010**, *46* (19), 3396-3398. DOI: 10.1039/C000964D.

(9) Schleucher, J.; Quant, J.; Glaser, S. J.; Griesinger, C. A Theorem Relating Cross-Relaxation and Hartmann-Hahn Transfer in Multiple-Pulse Sequences. Optimal Suppression of TOCSY Transfer in ROESY. *J. Magn. Reson., Ser. A* **1995**, *112* (2), 144-151. DOI: https://doi.org/10.1006/jmra.1995.1025.

(10) Thiele, C. M.; Petzold, K.; Schleucher, J. EASY ROESY: Reliable Cross-Peak Integration in Adiabatic Symmetrized ROESY. *Chem. Eur. J. A* **2009**, *15* (3), 585-588. DOI: https://doi.org/10.1002/chem.200802027.

(11) Mo, H.; Harwood, J. S.; Yang, D.; Post, C. B. A simple method for NMR t1 noise suppression. *J. Magn. Reson.* **2017**, *276*, 43-50. DOI: https://doi.org/10.1016/j.jmr.2016.12.014.

(12) Blundell, C. D.; Packer, M. J.; Almond, A. Quantification of free ligand conformational preferences by NMR and their relationship to the bioactive conformation. *Bioorg. Med. Chem.* **2013**, *21* (17), 4976-4987. DOI: https://doi.org/10.1016/j.bmc.2013.06.056.

(13) Salager, E.; Day, G. M.; Stein, R. S.; Pickard, C. J.; Elena, B.; Emsley, L. Powder crystallography by combined crystal structure prediction and high-resolution ^{1}H solid-state NMR spectroscopy. *J. Am. Chem. Soc.* **2010**, *132* (8), 2564-2566. DOI: 10.1021/ja909449k.

(14) Widdifield, C. M.; Robson, H.; Hodgkinson, P. Furosemide's one little hydrogen atom: NMR crystallography structure verification of powdered molecular organics. *Chem. Commun.* **2016**, *52* (40), 6685-6688. DOI: 10.1039/c6cc02171a PMID - 27115483.

(15) Webber, A. L.; Emsley, L.; Claramunt, R. M.; Brown, S. P. NMR Crystallography of Campho[2,3-c]pyrazole (Z′ = 6): Combining High-Resolution ^{1}H-^{13}C Solid-State MAS NMR Spectroscopy and GIPAW Chemical-Shift Calculations. *J. Phys. Chem. A* **2010**, *114* (38), 10435-10442. DOI: 10.1021/jp104901j PMID - 20815383.

(16) Corlett, E. K.; Blade, H.; Hughes, L. P.; Sidebottom, P. J.; Walker, D.; Walton, R. I.; Brown, S. P. An XRD and NMR crystallographic investigation of the structure of 2,6-lutidinium hydrogen fumarate. *Crystengcomm* **2019**, *21* (22), 3502-3516. DOI: 10.1039/C9CE00633H.

User guide for accompanying
Graphical User Interface
for data processing

Overview of Workflow

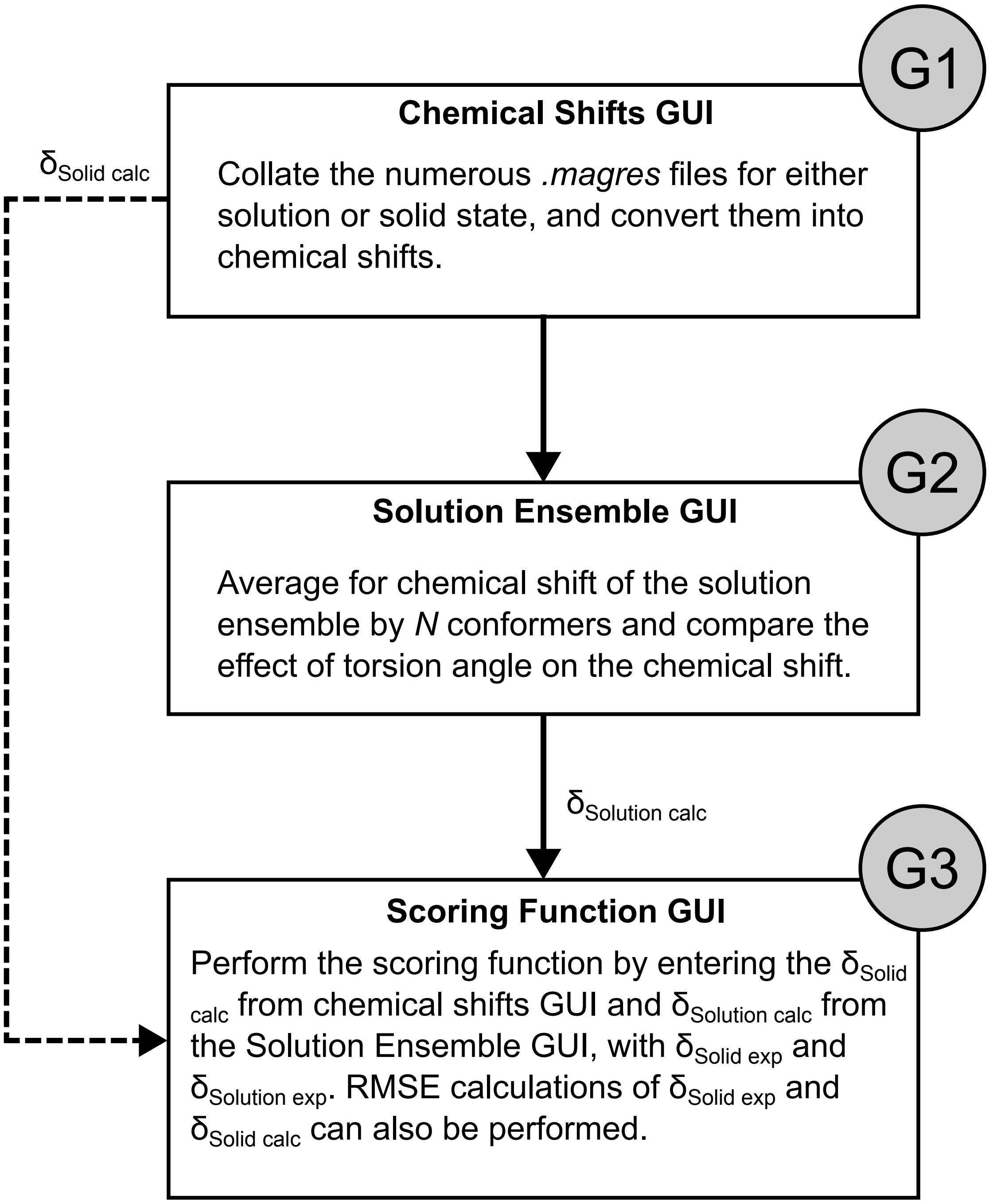

Schematic Workflow

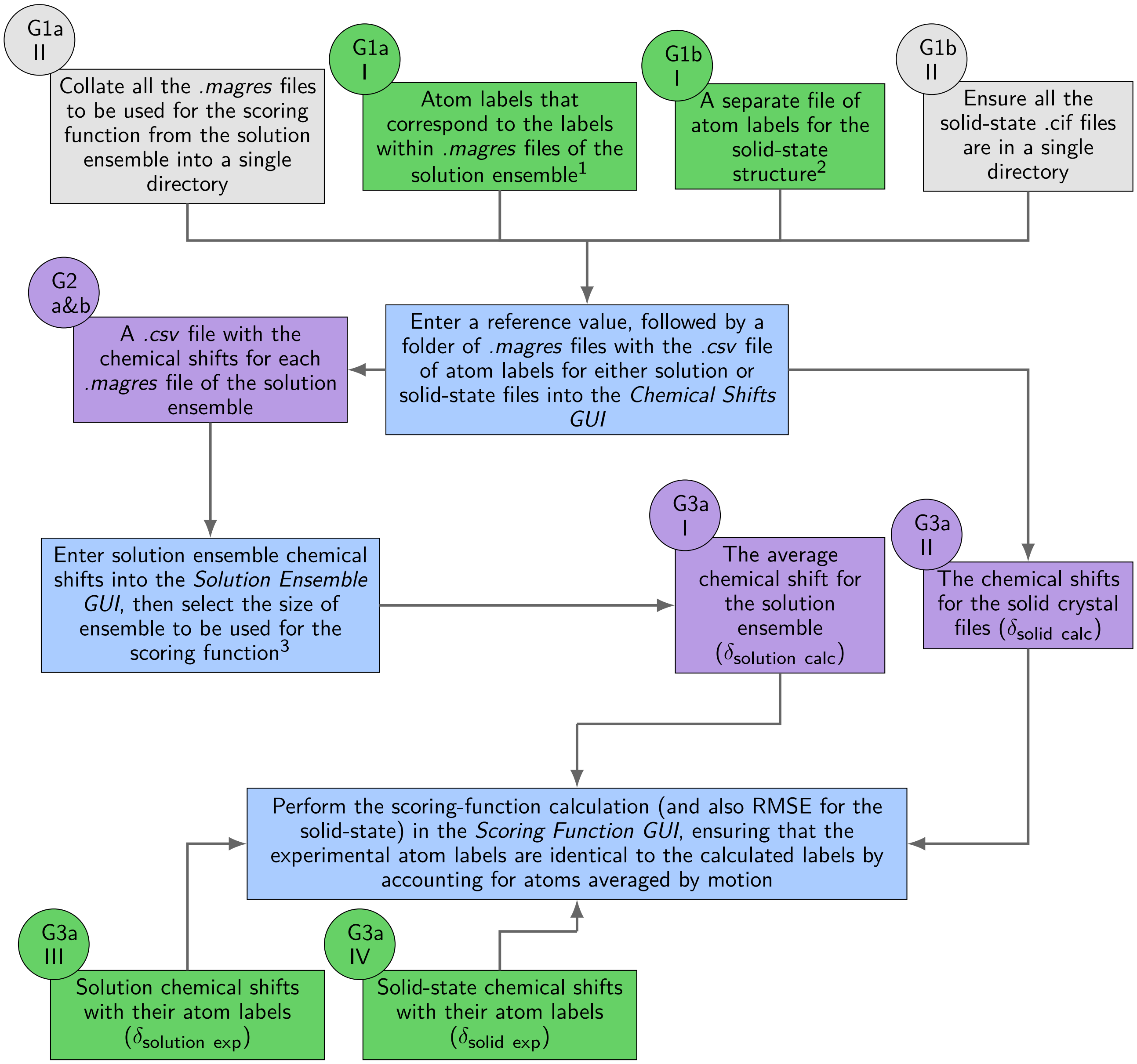

Key:
Grey - Input files.
Purple - Output files.
Blue - Graphical User Interface (GUI).
Green - A *.csv* file needs to be created.
Circle - Refers to each GUI in the documentation, where the roman numerals indicate the position of the input.

[1]Note labels will be constant across the solution ensemble *.cif* files.
[2]Noting that the labels change for each *.cif* file, and the column header must match the filename without extensions. If there are more than one molecule per unit cell, then insert *'_molA'* or *'_molB'* to the end of column name.
[3]Each atom site may converge with a different ensemble size.

Chemical shifts GUI

G1a Extracting solution chemical shifts into a single .csv file

(Note: The solution ensemble files must be separated by an '_' followed by their number e.g. 'name_1'), this can be done by softwares e.g. Babel.

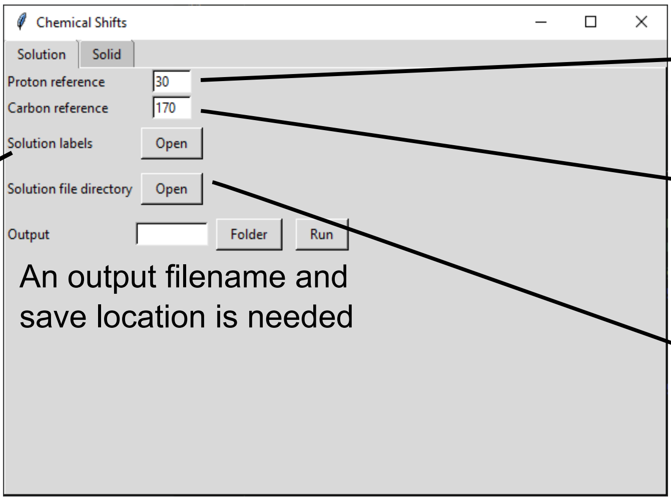

Enter ^{1}H reference value (30 by default).

Enter ^{13}C reference value (170 by default).

A directory for solution *.magres* files to be converted into chemical shifts is specified.

User Generated

My label	Solution Magres
H1	H1
H2	H2
H3	H3
H4	H4
H5	H5
H6	H6
H7	H7
H8	H8
H9	H9
H10	H10
C1	C1
C2	C2
C3	C3
C4	C4
C5	C5
C6	C6
C7	C7
C8	C8
C9	C9
C10	C10
C11	C11
C12	C12

Solution labels refer to a *.csv* file containing users custom labels e.g. *'My label'*, which correpond to the labels in the *.magres* file.

Output 1

(*)

My label	Fur_773.magres	Fur_1987.magres	Fur_295.magres
H1	7.0667	7.1157	7.0398
H2	5.9772	6.0097	5.9411
H3	5.7788	5.9946	5.7671
H4	4.7926	3.6364	3.4522
H5	3.5593	3.9559	4.2104
H6	8.6042	9.1213	8.0828
H7	6.4482	6.1864	6.504
H8	7.9956	8.0217	7.8727
H9	3.1656	4.538	3.133
H10	4.6675	3.1557	4.5375
H11	4.8188	4.8634	4.9052
C1	142.9409	143.1234	142.4584
C2	108.344	107.9127	108.1467
C3	105.9354	109.8521	105.914
C4	152.3404	151.768	154.5729
C5	35.5835	37.2505	35.265
C6	150.2973	149.8395	149.801
C7	110.6024	111.903	112.7887
C8	143.3685	142.1504	142.8847
C9	131.1223	131.3453	130.963
C10	133.7064	133.6889	132.7405
C11	103.2962	103.8551	104.5404
C12	169.1447	169.0953	169.7027

A *.csv* file containing the chemical shifts for all the input *.magres* files is produced.

G1b Extracting the calculated solid chemical shifts into a single *.csv* file

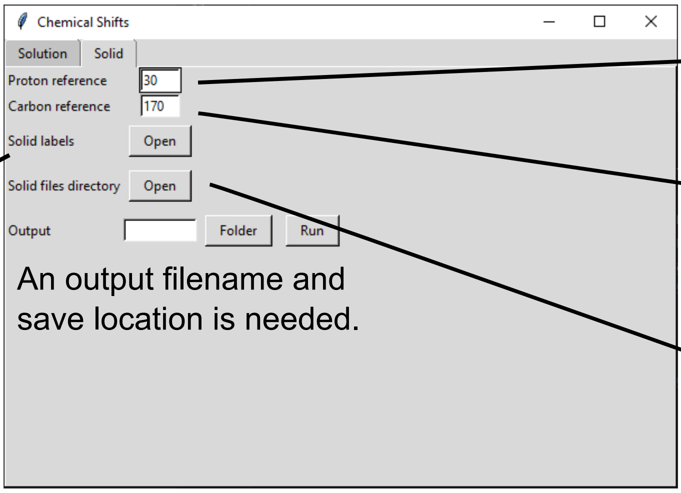

An output filename and save location is needed.

Enter ^{1}H reference value (30 by default).

Enter ^{13}C reference value (170 by default.)

A directory for solid *.magres* files to be converted into chemical shifts is selected.

User Generated

label	FURSEM13_NMR_molA	FURSEM13_NMR_molB	FURSEM14_NMR
H1	H27	H7	H17
H2	H23	H25	H21
H3	H21	H11	H25
H4	H13	H17	H9
H5	H15	H19	H13
H6	H31	H29	H29
H7	H5	H9	H5
H8	H1	H3	H1
H9	H37	H43	H37
H10	H41	H39	H41
H11	H35	H33	H33
C1	C47	C25	C37
C2	C41	C43	C41
C3	C39	C33	C45
C4	C45	C29	C17
C5	C35	C37	C33
C6	C19	C17	C21
C7	C21	C31	C5
C8	C5	C15	C13
C9	C3	C27	C29
C10	C1	C7	C1
C11	C13	C23	C25
C12	C11	C9	C9

Column header must match filenname without extension

Solid labels are different for each file, and each filename is needed without the *.magres* extenstion. If there are more than one molecule per .magres file, use *'_mol'* as a separator. Do not have any further underscores in the filename.

Output 2

label	FURSEM01_NMR_molA	FURSEM01_NMR_molB	FURSEM15_form1_NMR
H1	6.3972	7.7533	7.8685
H2	6.4241	5.9938	7.4615
H3	5.1043	5.9031	5.769
H4	4.6212	4.5214	3.6164
H5	3.8654	3.4992	3.4836
H6	7.9961	8.4557	8.0947
H7	7.198	5.0139	4.8844
H8	8.1216	7.9903	7.3252
H9	5.7912	6.8906	7.2933
H10	7.0155	7.2458	6.1508
H11	14.1813	14.3412	13.2779
C1	142.5233	145.7456	146.6416
C2	112.3228	110.2586	114.9906
C3	109.5858	112.2776	111.779
C4	155.3735	152.2619	147.0341
C5	36.6175	36.459	38.9586
C6	151.7128	150.7127	148.699
C7	116.3592	116.6053	111.2057
C8	141.9728	141.5158	145.9296
C9	126.0016	127.9526	130.1407
C10	136.0487	134.3644	134.2614
C11	104.9061	107.0035	105.7995
C12	173.5453	173.6375	175.0075

A single *.csv* file containing the chemical shifts for all the input *.magres* files is produced.

G2a Analysing dependence of the chemical shift for each atom and specific torsion angle

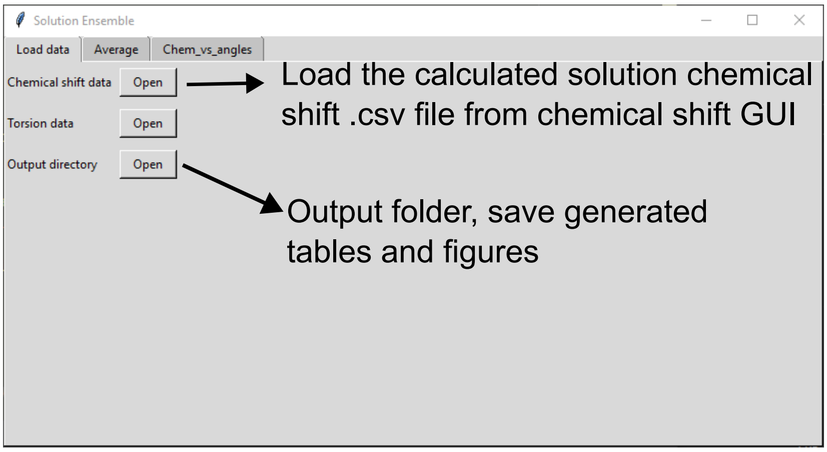

Requires Output 1 (from GUI G1a)

```
** Measure set 1: neutral_ensemble.sdf **
Torsion: mean = 181.96 sd = 113.27 range = [12.89,342.67] {O:8,C:7,C:9,N:12;}
1_ensemble4D_1 72.49
2_ensemble4D_2 36.56
3_ensemble4D_3 53.87
4_ensemble4D_4 285.99
5_ensemble4D_5 169.13
6_ensemble4D_6 298.27
7_ensemble4D_7 293.48
8_ensemble4D_8 180.35
9_ensemble4D_9 62.58
10_ensemble4D_10 58.72
11_ensemble4D_11 189.45
12_ensemble4D_12 88.48
13_ensemble4D_13 284.46
14_ensemble4D_14 36.20
15_ensemble4D_15 303.29
16_ensemble4D_16 80.74
17_ensemble4D_17 81.09
18_ensemble4D_18 52.85
19_ensemble4D_19 308.83
```

Torsion angles are generated from the dynamic solution ensemble and are passed as a '*.txt*' file. Each number corresponds to the number of the '*.magres*' file. For molecules with multiple rotatable bonds, an automatic separator is included, generating a table with the torsion angle for each rotatable bond.

G2b Determining $\delta_{solution\ calc}$

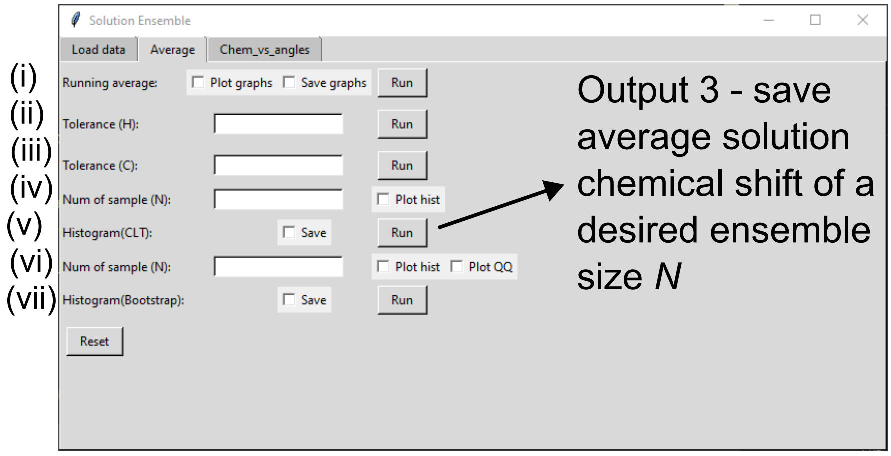

(i) A running average and running standard deviation can be viewed/saved for each of the '*.magres*' files.

(ii) & (iii) The number of samples are calculated to achieve a set tolerance e.g. a standard deviation of 0.1 ppm, which can be specified for each ^{1}H and ^{13}C.

(iv) & (v) The number of samples to be averaged and used as the calculated solution chemical shift ($\delta_{solution\ calc}$) are specified here. Further a histogram can be plotted to see the spread for each atomic shift.

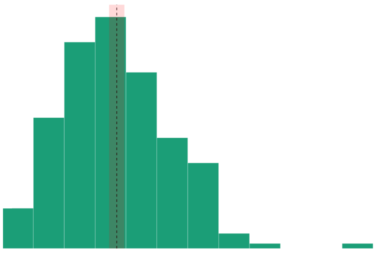

(vi) & (vii) In most cases, the spread does not appear to be normally distributed, therefore a select sample e.g. 1000 conformers are resampled (M = 1000 by default). This creates a bootstrap mean which can be used in place of the sample means (from d & e). A new histogram of the bootstrap means can be plotted, in addition to a Q-Q plot, which tests for normality.

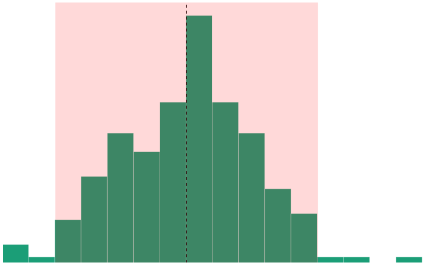

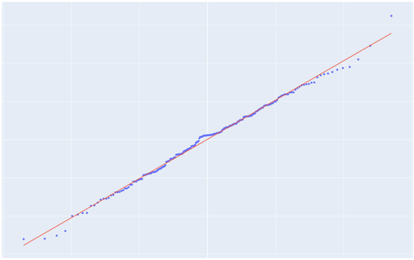

The red box in the histogram plot corresponds to the 95% confidence interval. Note the substantial increase for the bootstrap means, as compared to the plot for (iv) & (vi), which corresponds to a reduced standard error for the chemical shift.

G2c Chemical shift against torsion angle

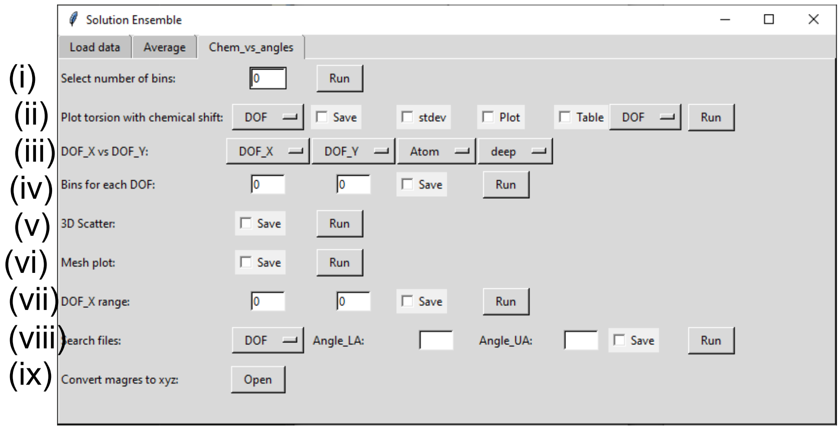

Degrees of Freedom (DOF) refers to the torsion angle for each rotatable bond

(i) Torsion angles can be grouped together by setting the number of bins, or plotted individually by setting the number of bins to zero.

(ii) The rotatable bond would need to be selected, which can either be plotted or tabulated. By default, the bins use the range but this can be changed to the st. dev. Further, the plot and or table can be saved if there is an output specified.

(iii) Comparison between two rotatable bonds can be made here for each atom. A colour scheme can be specified here *e.g.* deep.

(iv) Each rotatable bond can be grouped together in specified bins below each DOF in (j), which produces a contour plot.

(v) & (vi) A 3D scatter and mesh plot can be viewed respectively. The mesh plot uses the bins defined in (j).

(vii) Search for a range of files where one DOF is within the angles of another DOF. In this case all files of DOF_Y that are within the angles defined for DOF_X can be searched.

(viii) Search for files that are within the angles defined for each DOF (LA - lower angle, UA - upper angle)

(ix) Convert a *.magres* file into a *.xyz* file, to view the 3D conformation in other plotting programmes.

G3a Scoring Functions inputs

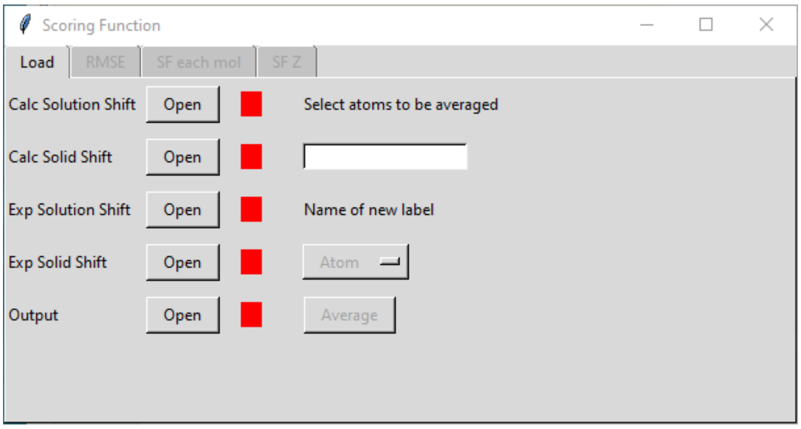

The scoring function procedure, which uses both calculated and experimental solution- and solid- state chemical shifts is performed here. The input .csv files containing calculated chemical shifts can be obtained from G1a (output 1) and G1b (output 2). Experimental solution- and solid- state chemical shifts are provided separately.

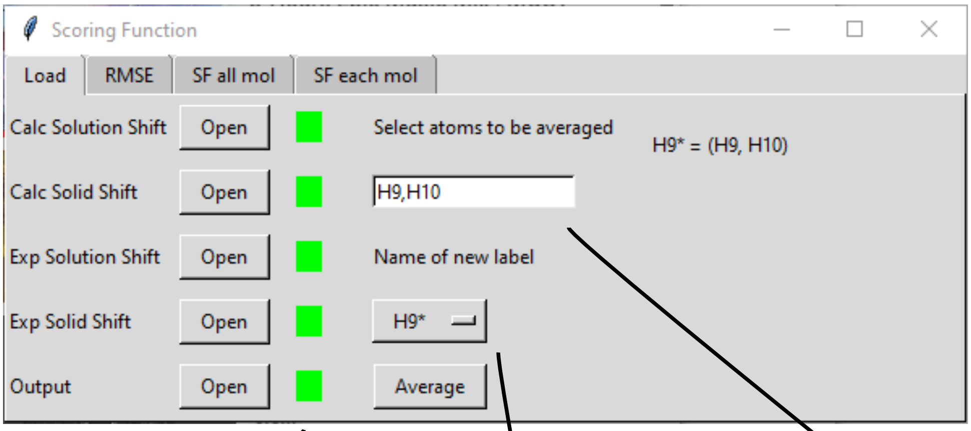

Once the files have been loaded and a folder to save the outputs, such as the scoring funciton tables, has been specified the boxes go green.

Select the experimental atom label, which is assigned to the average of the calculated chemical shifts.

Atom labels from the calculated chemical shifts that are to be averaged are written here.

G3b Calculate RMSE

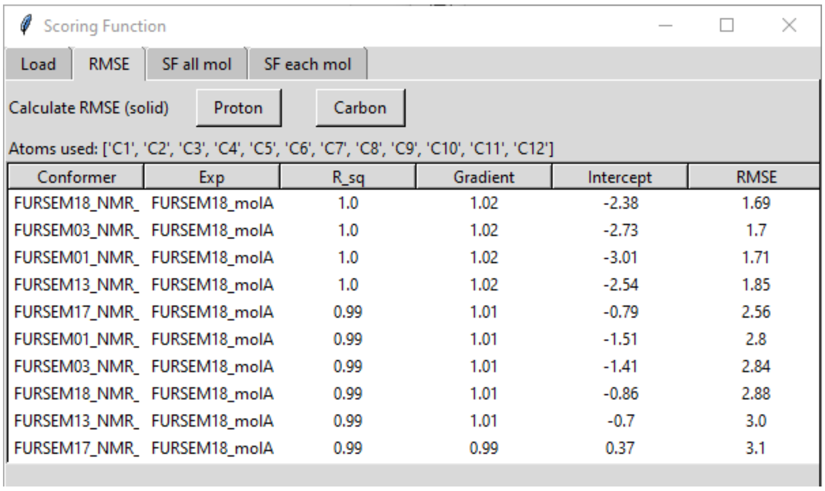

Scoring Function

Load | RMSE | SF all mol | SF each mol

Calculate RMSE (solid) Proton Carbon

Atoms used: ['C1', 'C2', 'C3', 'C4', 'C5', 'C6', 'C7', 'C8', 'C9', 'C10', 'C11', 'C12']

Conformer	Exp	R_sq	Gradient	Intercept	RMSE
FURSEM18_NMR_	FURSEM18_molA	1.0	1.02	-2.38	1.69
FURSEM03_NMR_	FURSEM18_molA	1.0	1.02	-2.73	1.7
FURSEM01_NMR_	FURSEM18_molA	1.0	1.02	-3.01	1.71
FURSEM13_NMR_	FURSEM18_molA	1.0	1.02	-2.54	1.85
FURSEM17_NMR_	FURSEM18_molA	0.99	1.01	-0.79	2.56
FURSEM01_NMR_	FURSEM18_molA	0.99	1.01	-1.51	2.8
FURSEM03_NMR_	FURSEM18_molA	0.99	1.01	-1.41	2.84
FURSEM18_NMR_	FURSEM18_molA	0.99	1.01	-0.86	2.88
FURSEM13_NMR_	FURSEM18_molA	0.99	1.01	-0.7	3.0
FURSEM17_NMR_	FURSEM18_molA	0.99	0.99	0.37	3.1

RMSE calculations using experimental and calculated solid-state chemical shifts. By default they are saved in the specified output directory. If there is a mismatch between labels i.e. a proton chemical shift is not averaged, an error will be shown.

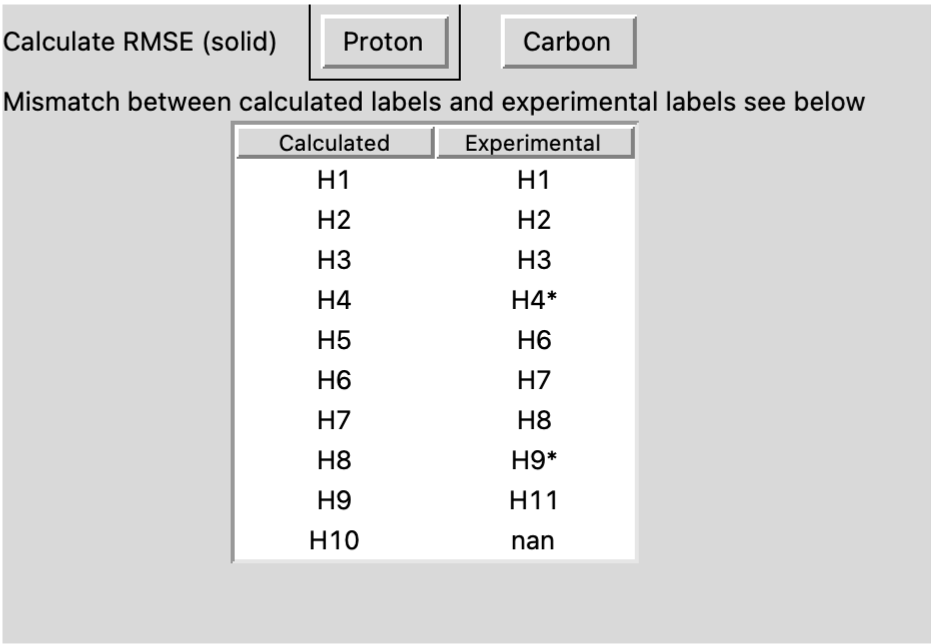

Calculate RMSE (solid) Proton Carbon

Mismatch between calculated labels and experimental labels see below

Calculated	Experimental
H1	H1
H2	H2
H3	H3
H4	H4*
H5	H6
H6	H7
H7	H8
H8	H9*
H9	H11
H10	nan

Error displaying mismatch between calculated and experimental labels. Non a number (nan) labels are ignored, however, experimental chemical shift labels that are not found for the calculated form raise an error.

G3c Scoring Function for all moleculese in the unit cell

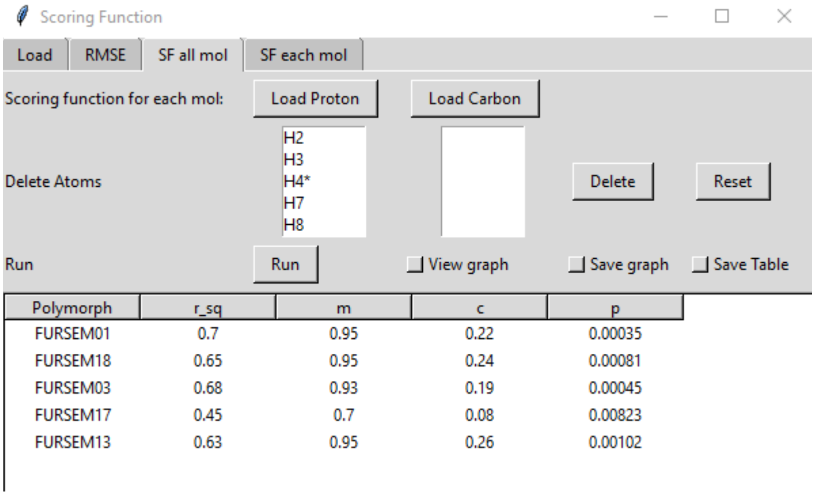

Polymorph	r_sq	m	c	p
FURSEM01	0.7	0.95	0.22	0.00035
FURSEM18	0.65	0.95	0.24	0.00081
FURSEM03	0.68	0.93	0.19	0.00045
FURSEM17	0.45	0.7	0.08	0.00823
FURSEM13	0.63	0.95	0.26	0.00102

The scoring function procedure can be performed by combining multiple molecules in the asymmetric unit cell together. This should account for any miss assignement or lack of chemical shift from either molecule.

G3d Scoring function performed for each molecule in the asymetric unit cell

Atoms in exchange or with unreliable chemical shifts can be removed here.

Select the nuclei.

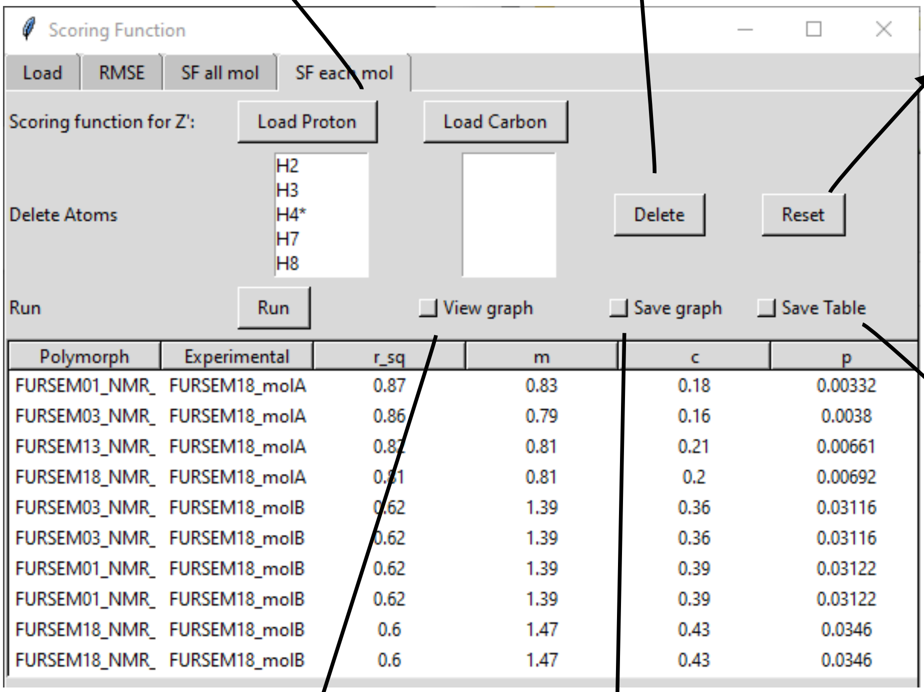

Polymorph	Experimental	r_sq	m	c	p
FURSEM01_NMR_	FURSEM18_molA	0.87	0.83	0.18	0.00332
FURSEM03_NMR_	FURSEM18_molA	0.86	0.79	0.16	0.0038
FURSEM13_NMR_	FURSEM18_molA	0.82	0.81	0.21	0.00661
FURSEM18_NMR_	FURSEM18_molA	0.81	0.81	0.2	0.00692
FURSEM03_NMR_	FURSEM18_molB	0.62	1.39	0.36	0.03116
FURSEM03_NMR_	FURSEM18_molB	0.62	1.39	0.36	0.03116
FURSEM01_NMR_	FURSEM18_molB	0.62	1.39	0.39	0.03122
FURSEM01_NMR_	FURSEM18_molB	0.62	1.39	0.39	0.03122
FURSEM18_NMR_	FURSEM18_molB	0.6	1.47	0.43	0.0346
FURSEM18_NMR_	FURSEM18_molB	0.6	1.47	0.43	0.0346

Reset will return all the atoms that were at the start of the calculation.

Save the table displayed in the image.

View the linear graphical plots of $\Delta\delta_{Calculated}$ vs $\Delta\delta_{Experimental}$.

Save graphical plots of $\Delta\delta_{Calculated}$ vs $\Delta\delta_{Experimental}$.
